# An integrative gene expression signature analysis identifies CMS4 *KRAS*-mutated colorectal cancers sensitive to combined MEK and SRC targeted therapy

**DOI:** 10.1186/s12885-022-09344-3

**Published:** 2022-03-10

**Authors:** Mingli Yang, Thomas B. Davis, Lance Pflieger, Michael V. Nebozhyn, Andrey Loboda, Heiman Wang, Michael J. Schell, Ramya Thota, W. Jack Pledger, Timothy J. Yeatman

**Affiliations:** 1 Department of Surgery & Molecular Medicine, University of South Florida, Tampa General Hospital Cancer Institute, 560 Channelside Drive, Tampa, FL 33602 USA; 2 Precision Genomics Translational Science Center, Intermountain Healthcare, 5026 South State Street, Murray, UT 84107 USA; 3 Sharp and Dohme, 770 Sumneytown Pike, Building 53, West Point, P.O. Box 4, Merck, PA 19486 USA; 4 Department of Biostatistics and Bioinformatics, Moffitt Cancer Center & Research Institute, 12902 Magnolia Drive, Tampa, FL 33612 USA; 5 Oncology Clinical Program, Intermountain Healthcare, 5026 South State Street, Murray, UT 84107 USA; 6 Huntsman Cancer Institute, University of Utah, 2000 Cir of Hope Dr, Salt Lake City, UT 84112 USA

**Keywords:** Colorectal cancer, MEK inhibitor, Targeted therapy, SRC, EMT, Cancer stem cell, CMS, Gene expression signature

## Abstract

**Background:**

Over half of colorectal cancers (CRCs) are hard-wired to RAS/RAF/MEK/ERK pathway oncogenic signaling. However, the promise of targeted therapeutic inhibitors, has been tempered by disappointing clinical activity, likely due to complex resistance mechanisms that are not well understood. This study aims to investigate MEK inhibitor-associated resistance signaling and identify subpopulation(s) of CRC patients who may be sensitive to biomarker-driven drug combination(s).

**Methods:**

We classified 2250 primary and metastatic human CRC tumors by consensus molecular subtypes (CMS). For each tumor, we generated multiple gene expression signature scores measuring MEK pathway activation, MEKi “bypass” resistance, SRC activation, dasatinib sensitivity, EMT, PC1, Hu-Lgr5-ISC, Hu-EphB2-ISC, Hu-Late TA, Hu-Proliferation, and WNT activity. We carried out correlation, survival and other bioinformatic analyses. Validation analyses were performed in two independent publicly available CRC tumor datasets (*n* = 585 and *n* = 677) and a CRC cell line dataset (*n* = 154).

**Results:**

Here we report a central role of SRC in mediating “bypass”-resistance to MEK inhibition (MEKi), primarily in cancer stem cells (CSCs). Our integrated and comprehensive gene expression signature analyses in 2250 CRC tumors reveal that MEKi-resistance is strikingly-correlated with SRC activation (Spearman *P* < 10^–320^), which is similarly associated with EMT (epithelial to mesenchymal transition), regional metastasis and disease recurrence with poor prognosis. Deeper analysis shows that both MEKi-resistance and SRC activation are preferentially associated with a mesenchymal CSC phenotype. This association is validated in additional independent CRC tumor and cell lines datasets. The CMS classification analysis demonstrates the strikingly-distinct associations of CMS1-4 subtypes with the MEKi-resistance and SRC activation. Importantly, MEKi + SRCi sensitivities are predicted to occur predominantly in the *KRAS* mutant, mesenchymal CSC-like CMS4 CRCs.

**Conclusions:**

Large human tumor gene expression datasets representing CRC heterogeneity can provide deep biological insights heretofore not possible with cell line models, suggesting novel repurposed drug combinations. We identified SRC as a common targetable node–-an Achilles’ heel–-in MEKi-targeted therapy-associated resistance in mesenchymal stem-like CRCs, which may help development of a biomarker-driven drug combination (MEKi + SRCi) to treat problematic subpopulations of CRC.

**Supplementary Information:**

The online version contains supplementary material available at 10.1186/s12885-022-09344-3.

## Background

Approximately 55% of colorectal cancers (CRCs) are driven by mutational activation of *KRAS, BRAF, NRAS*, and thus are hard-wired to oncogenic RAS/RAF/MEK/ERK pathway signaling [1–3]. CRC tumors with activated RAS/RAF pathway appear to be associated with poor outcomes [4]. Although RAS/RAF-mutated CRC has been targeted for the development of therapeutic inhibitors, they have been largely ineffective, likely due to complex resistance mechanisms such as intrinsic and adaptive resistance [5–12]. Rational combination therapies, based on a deep understanding of these resistance mechanisms, are the likely path forward.

Effective targeted therapy generally meets two necessary criteria: pathway activation and pathway addiction (dependency). Accordingly, *intrinsic resistance* is thought to be due to either absence of pathway activation or alternatively activation of signaling pathways that “bypass” targeted therapeutics. Recently, *adaptive resistance* (AR or “adaptive rewiring”) has emerged as a new drug resistance mechanism whereby normal homeostatic negative regulatory feedback of P-ERK on receptor tyrosine kinases (RTKs) is subverted, resulting in reactivation of the RAS/MEK pathway and/or activation of “bypass” signaling pathways [8–12]. Thus, AR can rapidly induce drug resistance to targeted therapy in tumors that are natively “sensitive”. For example, BRAFi (PLX4032 or vermurafenib) induces a rapid, marked feedback activation of EGFR in the *BRAF* (V600E)-mutated CRC cell lines, supporting continued proliferation in the presence of BRAF inhibition [9, 11]. Recent clinical trials report that combined BRAF and EGFR inhibition significantly improves response rates in *BRAF* (V600E) metastatic CRC patients (10% or 19%) [10, 13], and has now become the FDA-approved, standard of care. Despite progress in combination therapies in *BRAF* (V600E) mutated CRC, effective utilization of targeted therapies in the ~ 45% CRC patients who harbor *KRAS/NRAS* mutations remains a great challenge. Two well-characterized EGFRi therapies (cetuximab, panitumumab) have been FDA-approved in the first and second line, but only for wild-type *KRAS/NRAS* metastatic CRC patients [14]. Recently, a breakthrough has been made to target “undruggable” *KRAS* by taking advantage of a previously hidden groove on the protein surface in *KRAS* (G12C) [15]. However, response in metastatic CRC patients harboring *KRAS* (G12C) was not as robust. Since only ~ 3% of CRCs have mutant *KRAS* (G12C), therapeutic inhibitors of MEK (or ERK) downstream of KRAS/BRAF are essentially the only available RAS pathway targeted agents for a great majority of *KRAS*-mutated CRC patients.

Understanding the signaling biology of human cancer may be best served by directly examining a large number of genetically-diverse human tumors to develop relevant hypotheses. Here, we report a genome-wide analysis of multiple gene expression signatures of > 2000 molecularly-characterized primary and metastatic CRCs, that provides a new and deeper understanding of CRC oncogenic signaling and drug resistance mechanisms for which we have proposed SRC as a common targetable node. The SRC oncogene is a well-studied non-receptor tyrosine kinase [16–19]. Previously, our laboratory was the first to document that human CRC truncating mutations in the negative regulatory domain of SRC result in SRC activation [20]. Here, for the first time, we report a central role of SRC in mediating intrinsic and adaptive “bypass”-resistance to MEK inhibition (MEKi), primarily in cancer stem cells (CSCs), which may help development of a biomarker-driven drug combination (MEKi + SRCi) to treat problematic subpopulations of CRC.

## Methods

### Study design

The objective of our laboratory is to utilize large human tumor global molecular datasets as “best in class” direct models of human disease, containing complete tumor microenvironments, from which insightful observations may pave the way for the “fast-track” development of a biomarker-driven drug combination to treat problematic subpopulations of CRC. To investigate the MEKi-associated intrinsic and adaptive resistance, we have harnessed the power of multidimensional, quantitative gene expression signature scores in 2250 human CRC tumors hypothesis generation followed by validation analyses by two independent datasets of CRC tumors (*n* = 585 and *n* = 677, respectively) as well as the 154 CRC cell line dataset.

#### CRC human tumor and cell line datasets

(A) Moffitt CRC dataset (*n*= 2250). The cohort of 2250 colorectal adenocarcinoma tumors from distinct patients, including 1485 *primary* lesions and 764 *metastatic* lesions, with global gene expression analysis data from the surgical specimen (see Table 1), with samples obtained between October 2006 and September 2010, was used in various gene expression signature score correlation and survival analyses. All tumors were collected from curative resections followed by macrodisssection and snap freezing in liquid nitrogen within 15–20 min of extirpation. All the experiment protocol for involving human data was in accordance with the guidelines of national/international/institutional or Declaration of Helsinki. In all cases, tissue and clinical data were collected under the approval of the Institutional Review Board of Moffitt Cancer Center as part of the Total Cancer Care® (TCC) project (MCC14690) [21]. The informed written consent was obtained from participating patients [21]. The 2250 CRC tumors included a subset of 468 tumors we previously analyzed with global gene expression data, MSI status, and targeted gene sequencing of 1321 cancer-related genes [3, 4, 22]. Here we used this large, heterogeneous CRC tumor dataset in various gene expression signature score correlation and survival analyses.

**Table 1:** Summary of Metastatic/Primary Stages and CMS Classes in 2250 Moffitt Colorectal Cancers

CMS Classes^1^	N (%)	Age Median^2^	Sex^3^ N (%)	Metastatic^4^ N (%)	Primary^4^N (%)	Primary Stages^5^ N (%)				
						**I**	**II**	**III**	**IV**	**NA**
**CMS1**	305 (13.6)	68.7 yr (16.7 -87.0 yr)	**F** 182 (8.1) **M** 123 (5.5)	65 (2.9)	240 (10.7)	38 (2.6)	108 (7.3)	69 (4.6)	18 (1.2)	7 (0.5)
**CMS2**	675 (30.0)	63.0 yr (19.8 -87.5 yr)	**F** 283 (12.6) **M** 391 (17.4)	276 (12.2)	399 (17.7)	84 (5.7)	130 (8.8)	138 (9.3)	32 (2.2)	15 (1.0)
**CMS3**	347 (15.4)	66.6 yr (24.3 -87.1 yr)	**F** 176 (7.8) **M** 171 (7.6)	39 (1.7)	308 (13.7)	82 (5.5)	78 (5.3)	105 (7.1)	29 (2.0)	14 (0.9)
**CMS4**	685 (30.4)	62.0 yr (20.3 -87.6 yr)	**F** 316 (14.1) **M** 369 (16.4)	277 (12.3)	408 (18.1)	38 (2.6)	132 (8.9)	167 (11.2)	52 (3.5)	19 (1.3)
**CMS-NA**	238 (10.6)	64.4 yr (30.4 -85.9 yr)	**F** 109 (4.8) **M** 129 (5.7)	107 (4.8)	130 (5.8)	24 (1.6)	33 (2.3)	57 (3.8)	12 (0.8)	4 (0.3)
**Total**	2250 (100.0)	64.3 yr (16.7 -87.6 yr)	**F** 1066 (47.4) **M** 1183 (52.6)	764 (34.0)	1485 (66.0)	266 (17.9)	481 (32.4)	536 (36.1)	143 (9.7)	59 (4.0)

**Note:** ^1^CMS-NA tumors are those not applicable to any single CMS1-4 subtype;

^2^2147 patients had approximate age information;

^3^The percentages were calculated based on 2249 patients who had approximate sex information; F – female patients; M – male patients;

^4^The percentages were calculated based on 2249 patients who had approximate metastatic/primary tumor information;

^5^The percentages were calculated based on 1485 primary tumor patients among whom 1426 patients had approximate primary Stage I-IV information whereas 59 patients did not (NA)

(B) Marisa et al. CRC dataset (*n*= 585). To validate the findings from 2250 Moffitt CRCs, we performed analyses on the CRC patient sample dataset (*n* = 585) reported by Marisa et al. [23]. Note that we previously used Marisa CRC dataset to demonstrate that the cetuximab sensitivity signature score we developed was not prognostic [22]. Affymetrix gene expression data of these samples were downloaded via GEO with accession number GSE39582, whereas the *KRAS/BRAF* mutation status was adopted from Table S1 of Marisa et al.[23].

(C) TCGA CRC dataset (*n* = 677). We also used the TCGA CRC RNAseq dataset to validate the findings from 2250 Moffitt CRCs. As we did previously [22], TCGA COADREAD Level 3 RNAseq data (quantile normalized RSEM values) were downloaded from the Broad GDAC Firehose (https://gdac.broadinstitute.org/runs/stddata__2016_01_28/data/COAD/20160128/). The two downloaded tar file names are given as follows: gdac.broadinstitute.org_COADREAD.Merge_rnaseqv2__illuminahiseq_rnaseqv2__unc_edu__Level_3__RSEM_genes_normalized__data.Level_3.2016012800.0.0.tar; gdac.broadinstitute.org_COADREAD.Merge_rnaseqv2__illuminaga_rnaseqv2__unc_edu__Level_3__RSEM_genes_normalized__data.Level_3.2016012800.0.0.tar

(D) Medico et al. 154 CRC cell line dataset. Recently, a large number of human CRC cell lines were reported to be analyzed for genetic and transcriptional profiling of CRC cells and in vitro cetuximab sensitivity [24]. We previously used these cell line data to validate a cetuximab sensitivity signature score we developed [22]. Here we used their global gene expression data for correlation analysis. Affymetrix gene expression data of 155 cell lines were downloaded via GEO with accession number GSE59857. Note that CO115 (that was established from a tumor implanted into a nude mice) was excluded from analysis.

#### Gene expression signature scores

The gene lists of gene expression signatures including the 18-gene MEK pathway activation, 13-gene MEKi “bypass” resistance, SRC activation, 5-gene dasatinib sensitivity (Dasa-S), Hu-Lgr5-ISC, Hu-EphB2-ISC, Hu-Late TA, Hu-Proliferation, EMT, PC1 and 64-gene Wnt signatures are given in Additional File 1 (Table S1). A machine-readable GMT file of Table S1 is also provided as Additional File 2. Note that we have translated these signatures into mathematical scores (see below) that can be comparatively applied to thousands of tumors. We previously developed EMT and PC1 signature scores [25]. We also generated the 64-gene Wnt signature score [3] from a set of 64 “consensus” β-catenin (upregulated) genes adopted from a previously reported study [26]. Other signatures used including the 18-gene MEK pathway activation, 13-gene MEKi “bypass” resistance, SRC activation, 5-gene Dasa-S, Hu-Lgr5-ISC, Hu-EphB2-ISC, Hu-Late TA, Hu-Proliferation signatures were adopted from previous analyses reported from other groups [6, 27–29]. Of note, Broecker et al. reported a transcriptional signature induced by the metastasis promoting SRC-mutant that activated SRC signaling in breast cancer [27]. This 435-gene signature included (i) 61 up-regulated genes (UP), (ii) 50 down-regulated genes (DOWN), (iii) 163 “ + inf” genes that represented genes detectable in cells expressing SRC mutant but not detectable in the control cells), and (iv) 161 “-inf” genes that were detectable in the control cells but not detectable in cells expressing SRC mutant as described [27]. Our preliminary signature score correlation analysis in 2250 CRCs showed that the UP signature score was highly correlated with the (+ inf) score, the [UP – DOWN] score, the [(+ inf) – (-inf)] score or the more complex composite score [(UP + (+ inf)) – ((DOWN) + (-inf))] (All Spearman *P* < 10^–320^). For simplicity, here we elected to use the 61 UP genes for calculation of the SRC activation signature scores.

Furthermore, we generated a 5-gene dasatinib sensitivity (Dasa-S) signature *score* from the 5 up-regulated genes of a reported 6-gene dasatinib sensitivity signature (5 UP genes *EPHA2, CAV1, CAV2, ANXA1, PTRF* and 1 DOWN gene *IGFBP2*) that was developed in breast cell lines and validated in lung cell lines to predict sensitivity to dasatinib in solid tumors including breast, lung and ovary [28]. Notably, the DOWN gene *IGFBP2* was excluded here because it appeared to be not predictive of SRCi sensitivity in CRC cell lines and explant tumors [30]. All of the five up-regulated genes are targets of dasatinib, substrates for SRC family kinases, or part of signaling pathways downstream of SRC family kinases [28]. A validation analysis was conducted in multiple CRC cell lines (*n* = 50, see Additional File 3_Fig S1).

For the 2250 CRC and Marisa datasets, the signature scores were calculated for each tumor as previously described [4, 25]. Briefly, a score was computed for each of the signatures as the arithmetic mean of all probesets corresponding to gene symbols present in the corresponding gene signature. Notably, if both UP and DOWN signature genes were involved, we first calculated the UP scores and DOWN scores, respectively, and then calculated the (UP – DOWN) scores as the signature scores. Scores were standardized by subtracting the score median and dividing by the score IQR (interquartile range). The detailed data of standardized signature scores for 2250 CRC tumors are given in Additional File 4. The TCGA CRC dataset signature scores were calculated using the arithmetic mean of the RSEM values at the gene level. The signature scores generated from Marisa (*n* = 585) and TCGA (*n* = 677) CRC datasets are listed in Additional Files 5 and 6, respectively.

For the 154 CRC cell lines, we note that probe values of some signature genes appeared to differ one another in a few orders of magnitude. To avoid over-representation of only a few dominant probes or genes in calculating signature scores, individual probe values of a signature gene were normalized by the mean of all 154 cell lines prior to calculating signature scores. Scores were standardized by subtracting the score median and dividing by the score IQR (interquartile range) and are listed in Additional File 7. See Additional File 1 (Supplementary Methods) for additional supportive cell line analysis.

## Statistical methods

Correlation Analysis, The Kaplan Meier (KM) Survival Analysis, Welch’s T Test, Mann Whitney Test and Chi Square Trend Test as well as CMS classification: These statistical analyses were performed using GraphPad Prism version 8.00 (La Jolla, CA) and R version 3.6.2.

For CMS classification: Moffitt (*n* = 2250), Marisa et al. (*n* = 585) and TCGA (*n* = 677) CRC tumor samples as well as Medico CRC cell lines (*n* = 154) were classified by CMScaller (an R package for consensus molecular subtyping of colorectal cancer pre-clinical models) as described by Eide et al.[31] (see detailed CMS classification data in Additional Files 4–7).

CMS1*, CMS2*, CMS3* and CMS4* scores were generated for each of human CRC tumors and cell lines (see Additional Files 4–7). These CMS1-4* scores are designated to measure a propensity of a tumor to fall into CMS1, CMS2, CMS2 and CMS4 classes, respectively. We define: CMS1* = 1 – dCMS1; CMS2* = 1 – dCMS2; CMS3* = 1 – dCMS3; CMS4* = 1 – dCMS4, where dCMS1, dCMS2, dCMS3 and dCMS4 are the classification scores (0.00–1.00) generated by the CMScaller, which measure the distance of a tumor from the CMS1, CMS2, CMS3 and CMS4 templates, respectively [31]. For example, the higher the dCMS1 scores, the lower a propensity of a tumor to fall into the CMS1 class.

## Results

### Analysis of MEK activation *vs.* MEKi resistance signature scores in 468 human CRCs

Due to numerous genetic changes in tumors and the complexity of mechanisms underlying RAS signaling pathway, a gene expression signature-based pathway readout is thought to be more robust than a single molecular indicator in predicting RAS/MEK pathway activation, which is a pre-requisite for drug response to a MEK inhibitor [32]. Two gene expression signatures predictive of response/resistance to MEKi have been developed using large cell line panels of diverse tumor types including melanoma, lung and colon as reported by Dry et al.[6]. The first (an 18-gene signature) measures MEK pathway activation independent of the mutational status of *BRAF/RAS*, whereas the second (a 13-gene signature) predicts drug resistance caused by “bypass” proliferation/survival signaling pathways in the presence of active MEK [6]. We recently *adapted* the 18-gene MEK activation signature from use in fresh frozen CRC samples to more clinically available, archived formalin-fixed, paraffin-embedded (FFPE) tissues [33], as a means to predict RAS/MEK pathway dependence regardless of RAS/RAF mutation status. Moreover, in order to identify mutated genes beyond *KRAS*, *BRAF* and *NRAS* that might account for expanded RAS pathway activity, we also applied the 18-gene signature score to stratify 468 CRCs we previously characterized [3, 4, 34]. We identified *PTPRS*, a receptor-type protein tyrosine phosphatase, as one of the top-ranked 18-gene signature-associated mutated genes when the masking effects of mutant *KRAS*, *BRAF* and *NRAS* were iteratively removed [34].

**Fig. 1:**
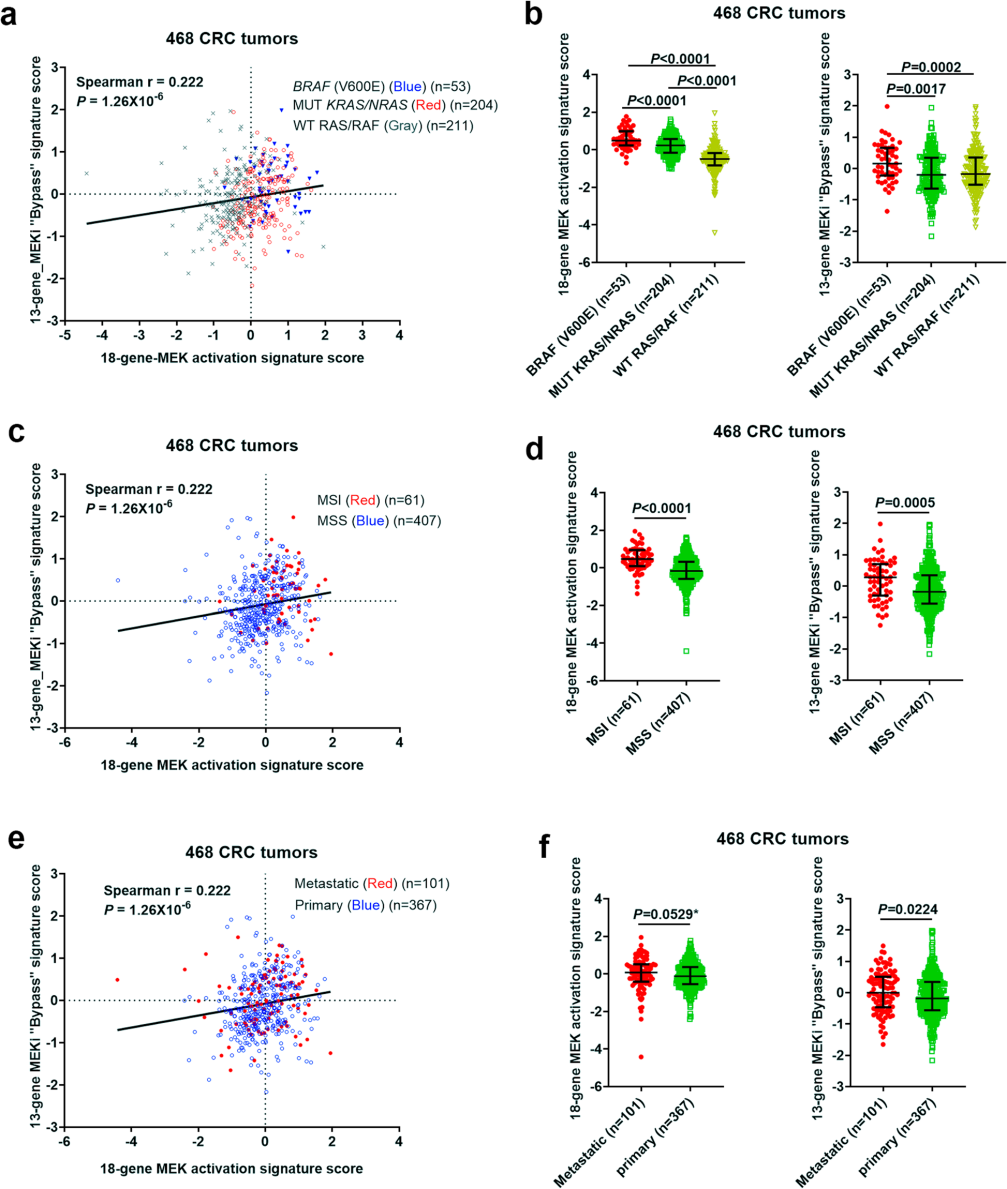
**Analysis of an 18-gene MEK pathway activation signature score *versus* a 13-gene MEKi “bypass” resistance signature score in 468 human CRCs. (a,b**) mutant vs WT RAS/*BRAF*; (**c,d**) MSI vs MSS tumors; (**e,f**) primary vs metastatic tumors. Spearman correlation of 18-gene vs 13-gene scores is shown (left panels). (Right panels) bars represent Median with interquartile range and *P* values are for two-tailed Mann Whitney test. The 18 gene MEK activation and the 13 gene MEKi bypass signature scores show a poor correlation indicating that these two scores measure independent properties

Since understanding complex resistance mechanisms to MEK inhibitors is currently an unmet need in RAS pathway targeted therapies, we carried out an analysis of the 18-gene MEK pathway activation signature score vs. the 13-gene MEKi “bypass”-resistance signature score in 468 CRC tumors (see Fig. 1). The analysis showed that the 18-gene and 13-gene signature scores had poor correlation (Fig. 1a), supporting the notion that the scores measure *independent* biology. While the 18-gene score measured MEK activation, the 13-gene signature measured “bypass” resistance, which could result from either *intrinsic* or *adaptive* mechanisms. It is noteworthy that the 13-gene signature was developed from cell lines treated with the MEKi for a 72 h period [6] during which AR could be induced [11]. As expected, tumors harboring mutant *BRAF* or *KRAS/NRAS* were shown to be preferentially associated with higher 18-gene MEK activation scores (i.e. > 0 (median) (Fig. 1a,b). Notably, a great majority of *BRAF*-mutated tumors had both higher 18-gene and higher 13-gene scores (Fig. 1a RUQ and Fig. 1b) suggesting *resistance* to MEKi. This is unexpected since a recent study using cell line panels predicted that *BRAF*-mutated tumor cells would be likely the most *sensitive* to MEKi [6]. However, this observation is actually in agreement with the recently reported adaptive resistance mechanism seen frequently occurring in *BRAF*-mutated tumors, initially sensitive to a RAS pathway targeted agent [8–12]. In support of this, a great of majority of MSI tumors, which were commonly associated with *BRAF* (V600E), had both higher 18-gene and higher 13-gene scores (Fig. 1c,d). *BRAF* mutant tumors (Fig. 1a) and MSI tumors (Fig. 1b) may represent the most likely to develop AR. Metastatic tumors (Fig. 1e) are represented in all quadrants. 18-gene and 13-gene scores differed only slightly between metastatic and primary tumors (Fig. 1f).

Of note, a significant percentage of mutant *KRAS* tumors had lower 18-gene scores (i.e. < 0 (median), Fig. 1a), supporting the notion that some *KRAS*-mutated CRC tumors are decoupled from RAS/MEK pathway activation [32], and these tumors should be excluded from MEKi therapies. On the other hand, some WT RAS/RAF tumors had higher 18-gene scores (i.e. > 0 (median)), suggesting these tumors may have *non-canonical* RAS pathway activation (e.g. through phosphatases such as PTPRS [34]) and may be candidates for MEKi treatment. This has been previously observed in lung cancer cell lines using a 147-gene RAS pathway activation score [32].

### Striking correlation of MEKi resistance with SRC activation in 2250 CRCs

**Fig. 2:**
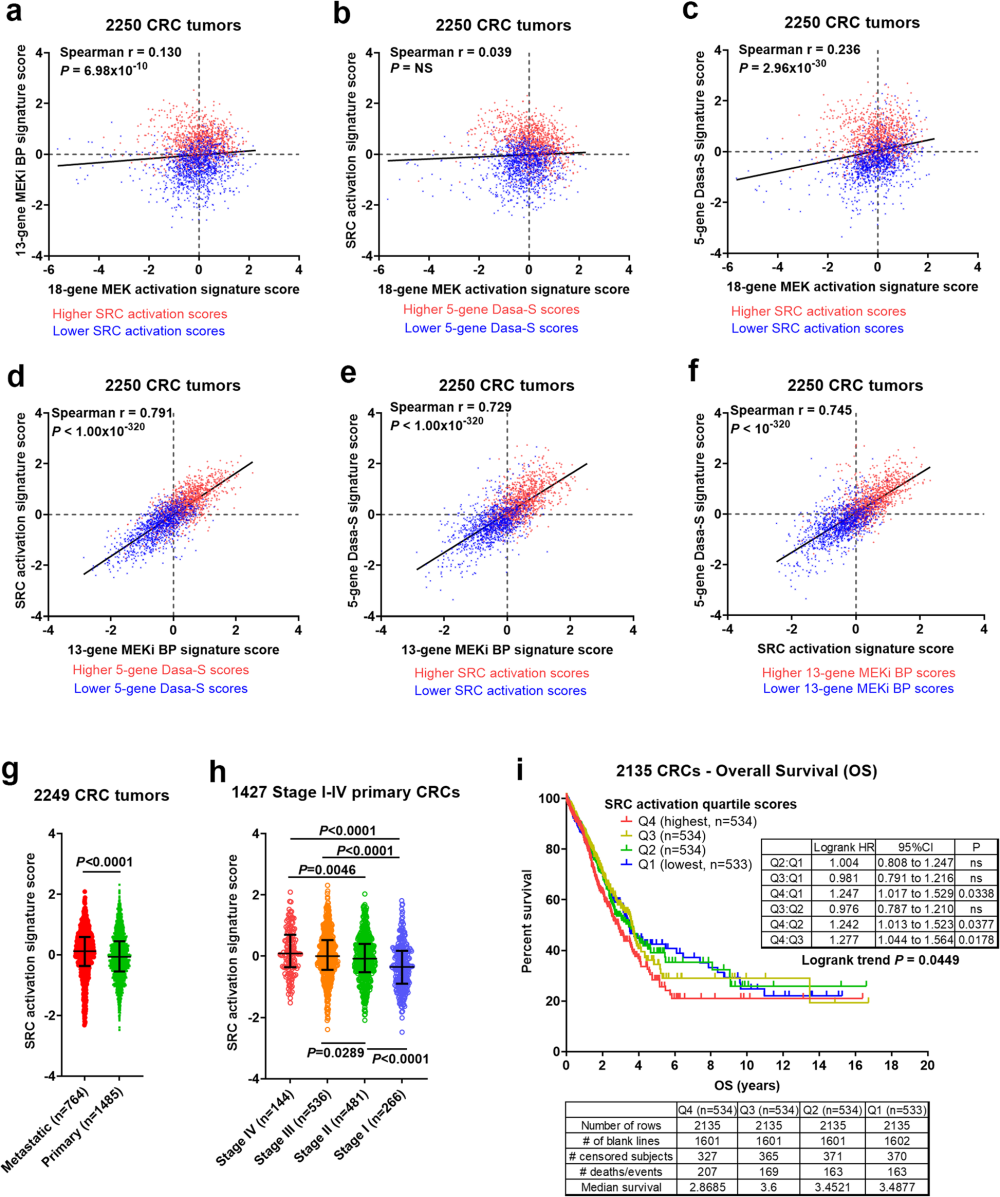
**The 13-gene MEKi “bypass” resistance score–-but not the 18-gene MEK pathway activation signature score–-was strongly correlated with SRC activation and 5-gene dasatinib sensitivity (Dasa-S) signature scores in 2250 human CRCs.** (**a-f**) Spearman correlation analyses of the 18-gene MEK activation, 13-gene MEKi bypass, SRC activation and 5-gene Dasa-S signature scores in 2250 CRCs. Higher (> 0 median) vs lower (< 0, median) scores are indicated by red vs blue colors. Comparison of SRC activation scores in 2249 primary vs metastatic tumors (**g**) and in 1427 Stage I vs II vs III vs IV primary tumors (**h**). Bars represent Median with interquartile range. *P* values are for two-tailed Mann Whitney test (right panels). (**i**) The Kaplan–Meier (KM) survival analysis of SRC activation quartile scores in 2135 CRCs which had corresponding overall survival (OS) data. SRC activation/dependency is a prominent feature of tumors expressing the 13-gene bypass-resistance pathway activities. The SRC activation signature score was significantly associated with metastasis and primary tumor stage I-IV progression and poor overall survival

We previously reported that PTPRS negatively regulated ERK activity in CRC cells [34]. We recently showed that PTPRS CRISPR-knock out sensitized CRC cells to the inhibitors of ERK in association with decreased SRC activity as measured by P-SRC Y419 [35]. Moreover, siRNA-knock down of SRC, or inhibition by dasatinib (SRCi), significantly increased ERKi-mediated apoptosis, suggesting a role of SRC in mediating resistance to RAS pathway targeted therapies [35]. This led us to investigate if SRC activation might contribute to the MEKi “bypass”-resistance mechanisms due to its known importance in advanced CRC previously reported by us and other groups [16–19]. For this purpose, we carried out gene expression signature score correlation and survival analyses in a large human CRC molecularly profiled dataset (*n* = 2250, including 468 tumors previously described [3]) (see Table 1 and Additional File 4**)**. The Spearman correlation analysis reveals that the 13-gene MEKi “bypass”-resistance score–-but not the 18-gene MEK pathway activation score–-was very strongly correlated with two independent gene expression signature scores measuring SRC activation/dependency (both *P* < 10^–320^) (Fig. 2a-e). Here, the first SRC activation score was generated from the transcriptional signature induced by a metastasis-promoting (human) SRC mutant harboring an L −  > A mutation at the C-terminal GENL motif of SRC[27, 36] (see **Methods**). Expression of this SRC mutant increased SRC activity (as measured by P-SRC Y419) and promoted migration and invasion [36], similar to a native, human truncating mutation in the negative regulatory domain of SRC (SRC 531) we previously identified in CRC [20]. The second SRC signature score was derived from 5 up-regulated genes of a 6-gene dasatinib sensitivity (Dasa-S) signature that was developed in breast cell lines and validated in lung cell lines to predict sensitivity to dasatinib in solid tumors including breast, lung and ovary [28] (see **Methods**). Note that the 5-gene Dasa-S signature score appeared to predict the trend of dasatinib sensitivity in multiple CRC cell lines (*n* = 50, Chi-square test for trend *P* = 0.0017) (see Additional File 3_Fig S1). Importantly, despite being independently developed, the SRC activation and the 5-gene Dasa-S scores were highly correlated with each other (*N* = 2250, *P* < 10^–320^, Fig. 2f), strongly supporting these scores as measures of SRC activation and dependency. Moreover, the SRC activation signature score was significantly associated with metastasis and primary tumor stage I-IV progression (Fig. 2g,h), in agreement with previous studies in CRC [37–40]. Furthermore, the Kaplan–Meier (KM) survival analysis shows that the SRC activation signature score was associated with poor overall survival (Fig. 2i).

### Strong correlation of SRC activation with EMT, regional metastasis and disease recurrence

**Fig. 3:**
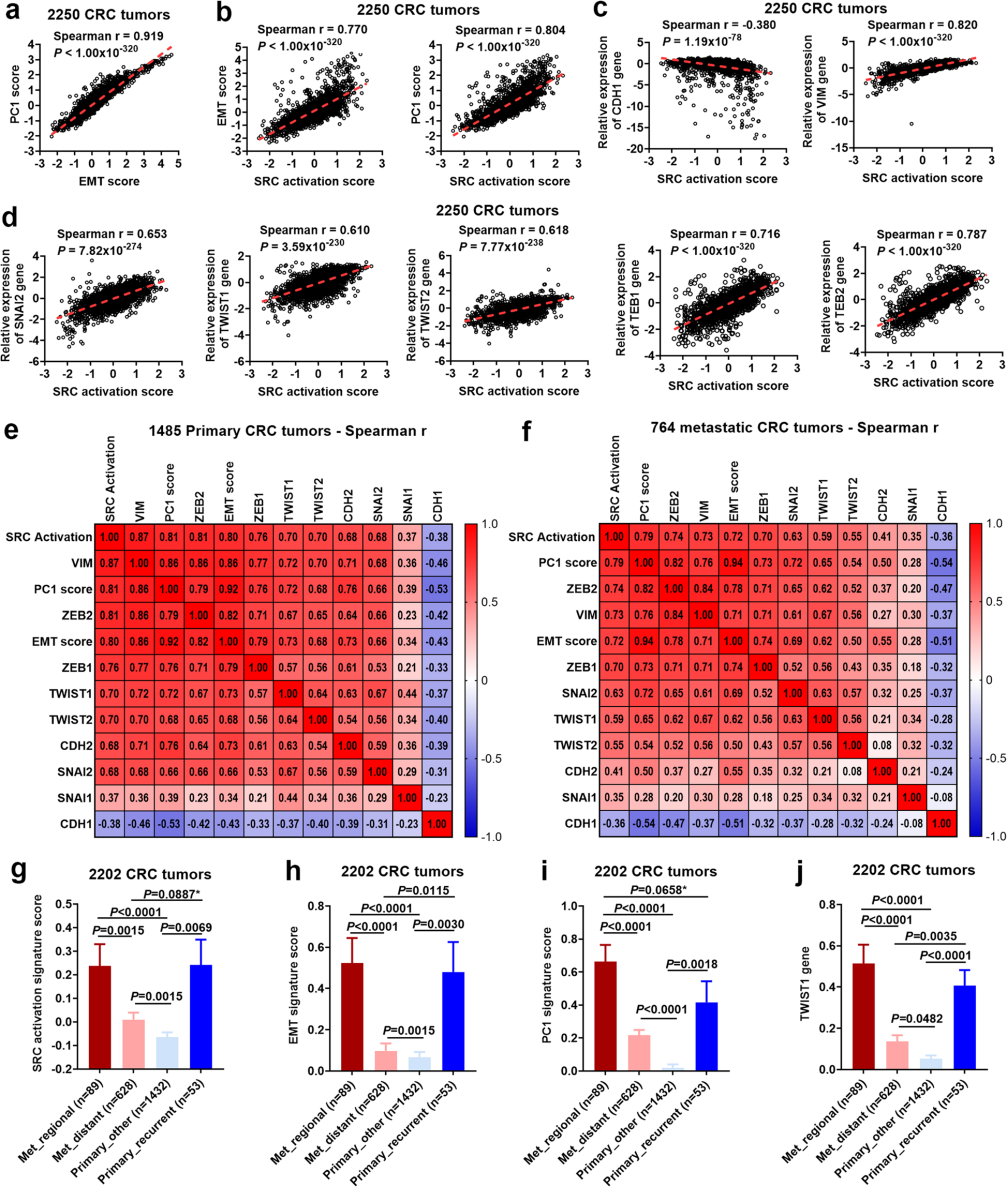
**SRC activation was highly correlated with EMT and its associated genes and was strongly associated with regional metastasis and disease recurrence.** Spearman correlation analyses in 2250 CRC tumors: (**a**) PC1 vs EMT signature scores and (**b-d**) SRC activation vs EMT, PC1, and *CDH1* (an epithelial marker), *VIM* (a mesenchymal marker), as well as EMT-genes *SMAIL2, TWIST1, TWIST2, ZEB1*, and *ZEB2*, respectively. Signature cores were standardized by subtracting the score median and dividing by the score IQR (interquartile range). The gene expression levels of individual EMT-related genes were also similarly standardized to give relative gene expression levels (median expression was set to 0). Spearman correlation heatmaps in (**e**) 1485 primary tumors and (**f**) 764 metastatic tumors. Comparison of (**g**) SRC activation, (**h**) EMT, and (**i**) PC1 signature scores as well as (**j**) *TWIST1* gene among 2202 metastatic (regional, distant) vs primary tumors (recurrent, other). Note that among 2250 tumors, 48 tumors without approximate data were excluded from analysis. Bars represent Mean with standard errors (SEM). *P* values are for two-tailed Mann Whitney test

Epithelial-to-mesenchymal transition (EMT) is a fundamental cellular program in embryonic development and tissue repair [41]. EMT promotes migration and motility and is aberrantly activated in cancer as a major mechanism promoting invasion and metastasis that induces tumor cells to acquire stem cell characteristics [41–43]. We previously developed an EMT signature that was found to be tightly-correlated (Pearson *r* = 0.92, *P* < 10^–135^) with the most dominant pattern of intrinsic gene expression in colon cancer (PC1, the first principal component)–both in gene identity and directionality in 326 colon tumors [25]. Now our new analysis in 2250 CRC tumors confirmed this tight correlation between EMT and PC1 signature scores (Spearman *r* = 0.92, *P* < 10^–320^, Fig. 3a), supporting the notion that EMT is arguably the dominant program in human CRC [25]. Importantly, we found that the SRC activation signature score was *strikingly-correlated* with both EMT and PC1 signature scores (both *P* < 10^–320^, Fig. 3b). This suggests that SRC activation may also be a dominant feature in CRC, which is supported by the observation that SRC activation was documented in > 80% of CRC [17]. The SRC-EMT association was further confirmed by the observation that SRC activation score was negatively- correlated with gene expression of *CDH1* (an epithelial marker) and positively-correlated with *VIM* (a mesenchymal marker) and other well-known EMT-genes including *SNAIL2*, *TWIST1*, *TWIST2*, *ZEB1* and *ZEB2* (Fig. 3c,d). It is noteworthy that the same strong correlations were seen between 1485 primary tumors and 764 metastatic tumors (Fig. 3e,f). Furthermore, we found that the SRC activation was significantly associated with regional metastasis and disease recurrence (Fig. 3g). Similar associations were also seen for EMT and PC1 signature scores as well as gene expression of the EMT genes (e.g. *TWIST1*) (Fig. 3h-j and Additional File 3_Fig S2). Collectively, these data suggest that SRC activation may play an important role in CRC progression, metastasis and disease recurrence by inducing EMT. Note that expression of *SNAIL2, ZEB1, ZEB2, TWIST2* was significantly lower in the distant metastatic tumors (Met_distant, *n* = 628) than other subgroups (see Fig S2).

### Preferential association of MEKi resistance and SRC activation with “mesenchymal-like” stemness

**Fig. 4:**
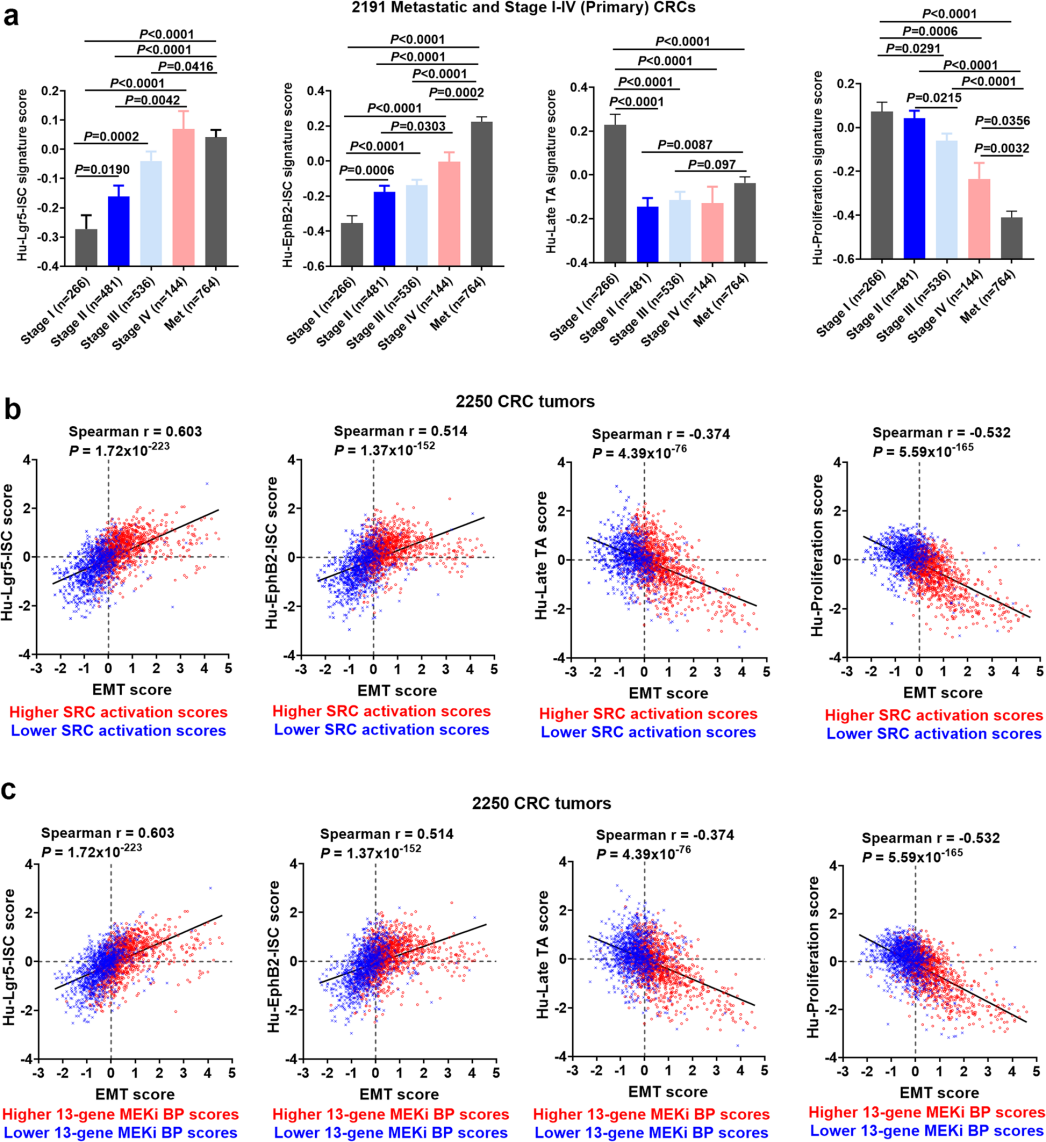
**The SRC activation and 13-gene MEKi “bypass” resistance signature scores were associated with “mesenchymal-like” stemness.** (**a**) Comparison of Hu-Lgr5-ISC, Hu-EphB2-ISC, Hu-Late TA, and Hu-Proliferation signature scores among 2191 metastatic vs primary Stage IV vs III vs II vs I tumors. Bars represent Mean with standard errors (SEM). *P* values are for two-tailed Mann Whitney test. (**b,c**) Scatter plots of Hu-Lgr5-ISC, Hu-EphB2-ISC, Hu-Late TA, and Hu-Proliferation vs EMT signature scores, respectively (n = 2250 CRCs). Higher (> 0 median) vs lower (< 0, median) scores are indicated by red vs blue colors for (**b**) SRC activation and (**c**) 13-gene MEKi bypass resistance signatures

SRC is necessary and sufficient to drive intestinal stem cell (ISC) proliferation during tissue self-renewal, regeneration and tumorigenesis in vivo [44]. Cancer stem cells (CSCs) are thought to be resistant to targeted therapies and responsible for metastasis, disease recurrence and poor outcomes [42, 45]. Intestinal stem cell (ISC) signatures derived from *normal crypts* have been reported to identify CRC CSCs and predict disease relapse [29]. The ISC-specific genes identified a stem-like cell population positioned at the bottom of tumor structures reminiscent of crypts and ISC-like tumors cells displayed robust tumor-initiating capacity in immunodeficient mice as well as long-term self-renew potential [29]. Here we show that humanized ISC signature scores (Hu-EphB2-ISC; Hu-Lgr5-ISC), but not *non-stemness* signature scores such as Hu-Late TA and Hu-Proliferation [29], were significantly associated with tumor progression in 2191 Stage I-IV (primary) and metastatic CRCs (Fig. 4a). Note that for each tumor, we generated and standardized four signature scores from the humanized signature gene lists [29] (see **Methods**), as we did previously for a composite prognostic signature [4]. We found that both the Hu-EphB2-ISC and the Hu-Lgr5-ISC signature score were positively (but modestly) correlated with the EMT signature score (see Fig. 4b,c). This is likely due to stem cell plasticity consisting of not only *mesenchymal* stemness but also *epithelial* stemness. However, higher SRC activation or higher MEKi “bypass” scores were preferentially associated with tumors with both higher “stemness” (Hu-EphB2-ISC or Hu-Lgr5-ISC) and higher EMT signature scores (Fig. 4b,c left two panels, see “right and upper” quadrants (RUQs)), suggesting that “mesenchymal-like” CSCs may primarily contribute to SRC-mediated MEKi “bypass” resistance. It is noteworthy that higher SRC activation or higher MEKi “bypass” scores were associated with “mesenchymal-like” tumors that had lower Hu-Late TA and Hu-Proliferation scores (Fig. 4b,c right two panels, see “right and lower” quadrants (RLQs)), suggesting that SRC-activated and MEKi-resistant CRCs were weakly-proliferative, CSC-like tumors.

### Strong association of SRC-mediated MEKi resistance with the mesenchymal CSC-like CMS4 subtype

**Fig. 5:**
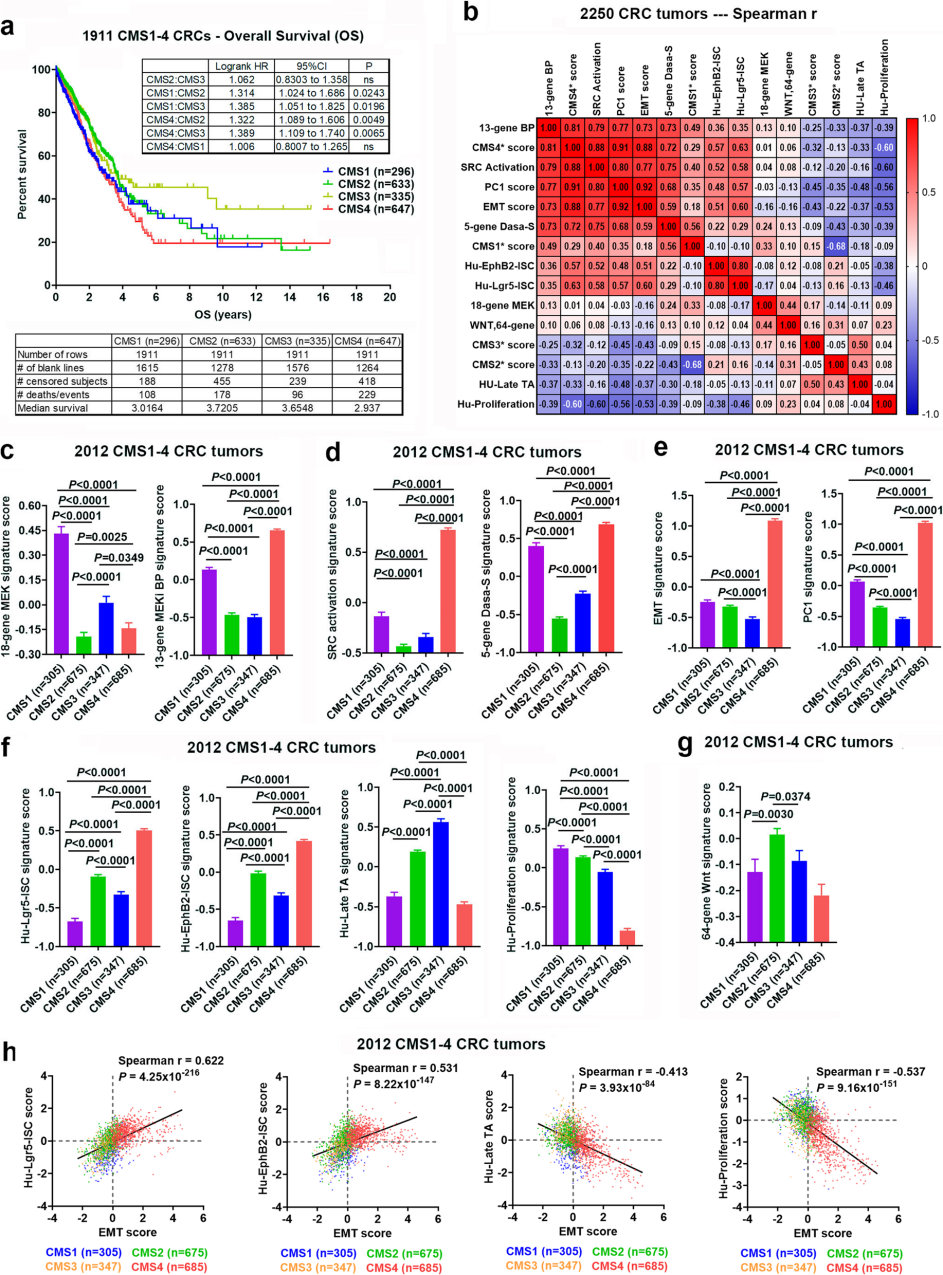
**Correlation analysis of the signature scores with the CMS1-4 subtypes.** (**a**) The CMS classification system was used to classify 2250 CRC tumors: CMS1 (*n* = 305), CMS2 (*n* = 675), CMS3 (*n* = 347) and CMS4 (*n* = 685) as well as CMS-NA (*n* = 238) (that are not applicable to any single CMS1-4 subtype). The Kaplan–Meier (KM) survival analysis shows that the CMS1 and CMS4 tumors (vs CMS2 and CMS3 tumors) were significantly associated with poor overall survival (OS). (**b**) Spearman correlation heatmap of the signature scores with CMS1*, CMS2*, CMS3* and CMS4* scores (measuring a propensity of a tumor to fall into CMS1, CMS2, CMS2 and CMS4 classes, respectively) in 2250 CRC tumors. 13-gene BP –- 13-gene MEKi bypass resistance; 5-gene Dasa-S –- 5-gene dasatinib sensitivity; 18-gene MEK –- 18-gene MEK pathway activation. (**c-g**) Comparison of the signature scores among the CMS1-4 subtypes (*n* = 2012). Bars represent Mean with standard errors (SEM). *P* values are for two-tailed Mann Whitney test. (**h**) Scatter plots of Hu-Lgr5-ISC, Hu-EphB2-ISC, Hu-Late TA, and Hu-Proliferation vs EMT signature scores. The CMS1-4 subtypes are indicated by red (CMS4) vs orange (CMS3) vs green (CMS2) vs blue (CMS1) colors

Heterogenous CRC has been recently classified into four consensus molecular subtypes (CMS1-4) with distinguishing features: **CMS1 (MSI, immune**), hypermutated, microsatellite unstable and strong immune activation; **CMS2 (canonical)**, epithelial, marked WNT and MYC signaling activation; **CMS3 (metabolic)**, epithelial and with evident metabolic dysregulation; and **CMS4 (mesenchymal)**, characterized by transforming growth factor– β activation, stromal invasion and angiogenesis [46]. While CMS1 tumors were associated with worse survival after relapse, CMS4 tumors were also enriched for *cancer stemness* and associated with worse overall survival and worse relapse-free survival [46]. Here, using global gene expression analysis, we have applied this CMS classification system to our 2250 CRC tumors. The KM survival analysis found that the CMS1 and CMS4 tumors were associated with poor OS (Fig. 5a). In addition, we have generated CMS1*, CMS2*, CMS3* and CMS4* scores for each tumor (see **Methods** and Additional File 4). These scores are measure a propensity of a tumor to fall into CMS1, CMS2, CMS2 and CMS4 classes, respectively. Strikingly, we found that the 13-gene MEKi-resistance, SRC activation, 5-gene Dasa-S, EMT, PC1 and “stemness” (Hu-EphB2-ISC, Hu-Lgr5-ISC) signature scores were all strongly correlated with the CMS4* scores but had weak or negative correlations with the CMS2* or CMS3* scores (Fig. 5b). Similar correlation patterns were also seen when separate into 1485 primary tumors and 764 metastatic tumors (see Additional File 3_Fig S3 and S4). This is supported by a comparison analysis among 2012 CMS1-4 classified tumors showing that these signature scores were all *significantly higher* (*P* < 0.0001) in CMS4 than other CMS subtypes (Fig. 5c-f). These data strongly suggest CMS4 tumors are associated with SRC-mediated MEKi-resistance. Intriguingly, the 13-gene MEKi-resistance, SRC activation and 5-gene Dasa-S scores in CMS1 were the second highest among CMS1-4 subtypes and significantly higher (*P* < 0.0001) than either CMS2 or CMS3 (Fig. 5c,d**)**. This suggests that CMS1 tumors, to a lesser extent than CMS4 tumors, were also associated with SRC-mediated MEKi-resistance. Notably, CMS3 tumors had highest Hu-Late TA scores, whereas CMS2 tumors were highest in the 64-gene Wnt scores (Fig. 5f,g).

Importantly, Spearman correlation analyses of Hu-Lgr5-ISC, Hu-EphB2-ISC, Hu-Late TA, and Hu-Proliferation vs EMT signature scores confirmed that CMS4 tumors were preferentially associated with a weak-proliferative, mesenchymal CSC phenotype (Fig. 5h).

### Validation analysis of the association of MEKi resistance with SRC activation/mesenchymal-like stemness/CMS4 by two independent CRC tumor datasets

**Fig. 6:**
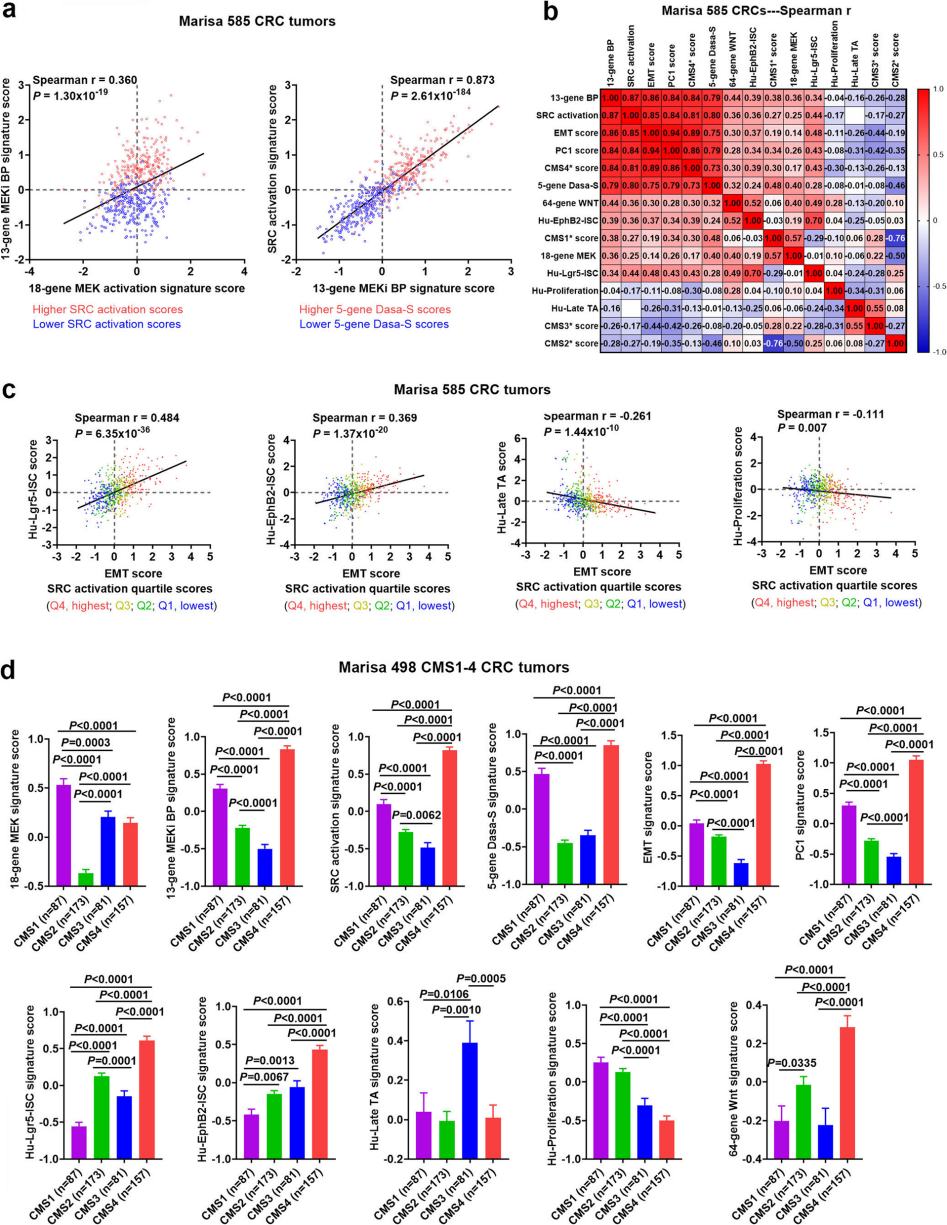
**Validation analysis using the Marisa 585 CRC tumor dataset.** (**a**) Scatter plots of the 18-gene MEK activation vs. the 13-gene MEKi bypass (BP) and the 13-gene MEKi BP vs. SRC activation signature scores in Marisa 585 CRCs. Higher (> 0 median) vs lower (< 0, median) scores are indicated by red vs blue colors. (**b**) Spearman correlation heatmap of the signature scores and CMS1*, CMS2*, CMS3* and CMS4* scores (measuring a propensity of a tumor to fall into CMS1, CMS2, CMS2 and CMS4 classes, respectively) in Marisa 585 CRC tumors. (**c**) Scatter plots of Hu-Lgr5-ISC, Hu-EphB2-ISC, Hu-Late TA, and Hu-Proliferation vs EMT signature scores, respectively (*n* = 585 CRCs). The quartile scores (Q1-Q4) of SRC activation signature are indicated by different colors (Q1, blue; Q2, green; Q3, yellow; Q4 red). (**d**) Comparison of the signature scores among the CMS1-4 subtypes (*n* = 498). Bars represent Mean with standard errors (SEM). P values are for two-tailed Mann Whitney test

**Fig. 7:**
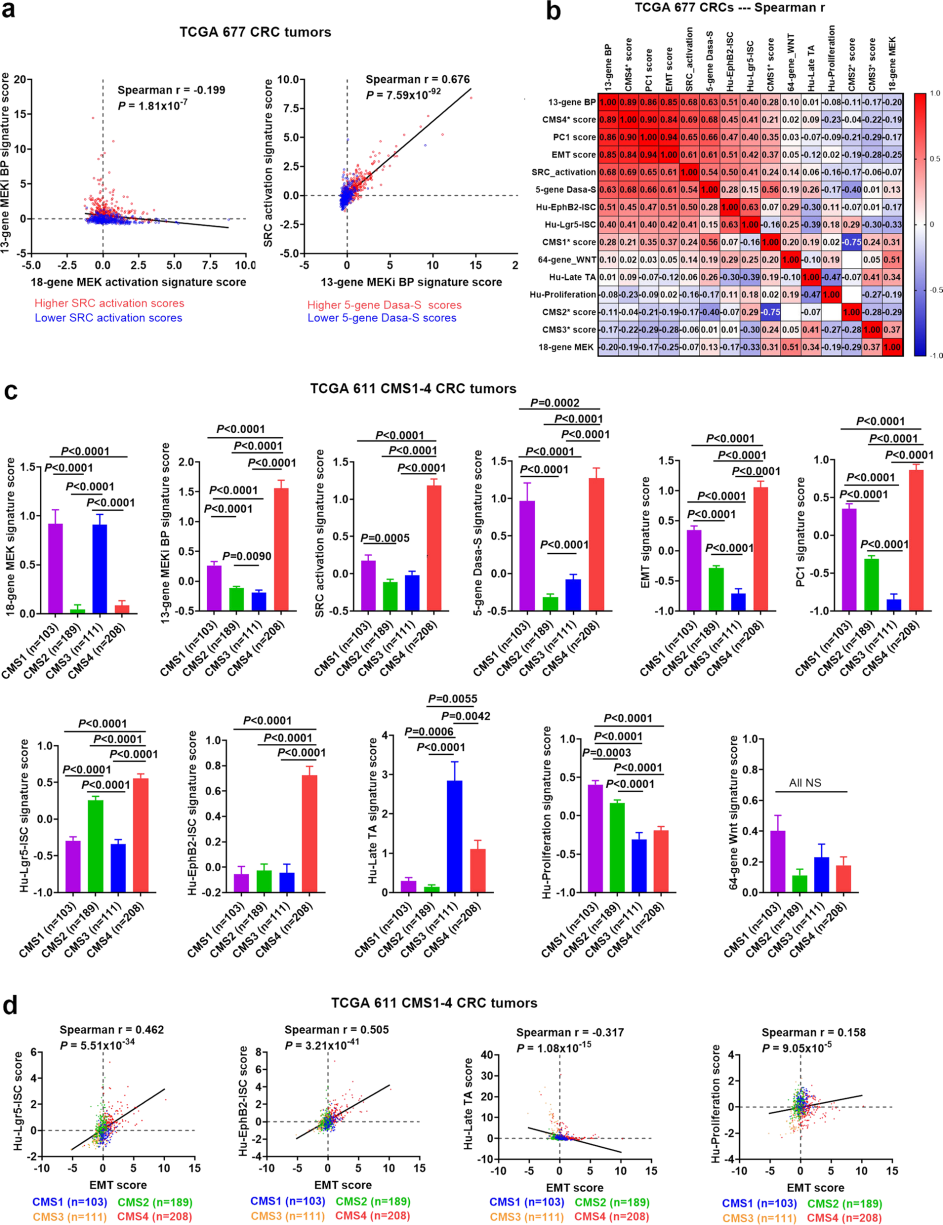
**Validation analysis using the TCGA 677 CRC tumor dataset.** (**a**) Scatter plots of the 18-gene MEK activation vs. the 13-gene MEKi bypass (BP) and the 13-gene MEKi BP vs. SRC activation signature scores in TCGA 677 CRCs. Higher (> 0 median) vs lower (< 0, median) scores are indicated by red vs blue colors. (**b**) Spearman correlation heatmap of the signature scores and CMS1*, CMS2*, CMS3* and CMS4* scores (measuring a propensity of a tumor to fall into CMS1, CMS2, CMS2 and CMS4 classes, respectively) in TCGA 677 CRC tumors. (**c**) Comparison of the signature scores among the CMS1-4 subtypes (*n* = 611). Bars represent Mean with standard errors (SEM). P values are for two-tailed Mann Whitney test. (**d**) Scatter plots of Hu-Lgr5-ISC, Hu-EphB2-ISC, Hu-Late TA, and Hu-Proliferation vs EMT signature scores. The CMS1-4 subtypes are indicated by red (CMS4) vs orange (CMS3) vs green (CMS2) vs blue (CMS1) colors

We validated the findings from > 2000 Moffitt CRC tumors using two independent, publicly-available CRC human datasets (Marisa 585 CRCs [23] and TCGA 677 CRCs [47], see **METHODS**). Again, the MEKi bypass resistance was strongly correlated with the SRC activation/dasatinib sensitivity, the EMT-like stemness and the CMS4 subtype (see Fig. 6 and Fig. 7 as well as Additional File 3_Fig S5 and Fig S6). The strong association of MEKi resistance with SRC-mediated EMT biology was also supported by its correlation with expression of the mesenchymal marker *VIM* and other known EMT-associated genes (Additional File 3_Fig S7 and Fig S8).

### MEKi + SRCi sensitivities are predicted to occur predominantly in the *KRAS*-mutant, CMS4 and *BRAF* (V600E), CMS1 CRCs

**Fig. 8:**
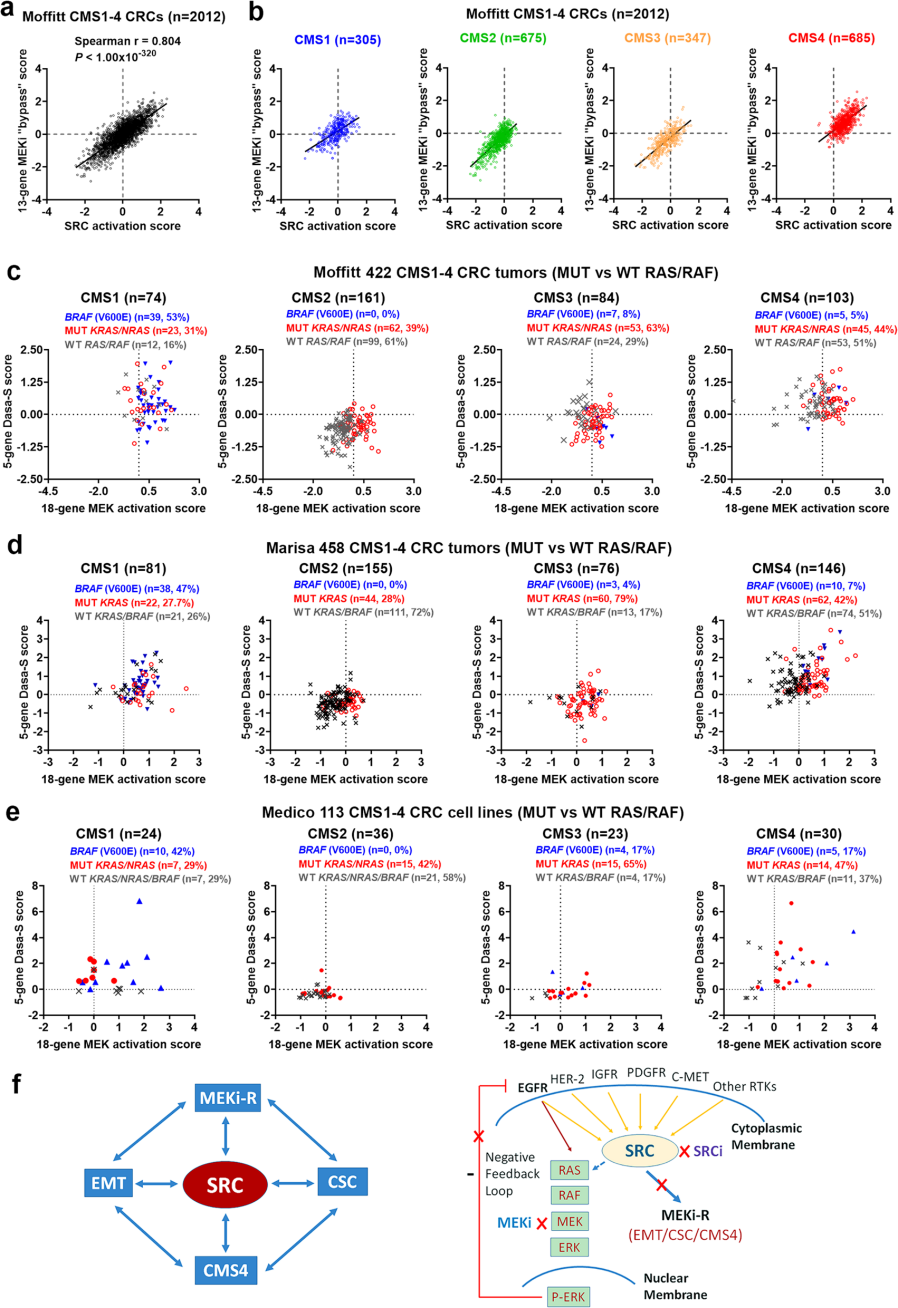
**MEKi + SRCi were predicted to occur predominantly in the *KRAS*-mutant, CMS4 CRCs.** (**a**) Spearman correlation analysis of the 13-gene MEKi bypass vs. SRC activation signature scores in Moffitt 2012 CMS1-4 CRCs. (**b**) Scatter plots of 13-gene MEKi bypass vs. SRC activation scores are shown in CMS1 (*n* = 305), CMS2 (*n* = 675), CMS3 (*n* = 347) and CMS4 (*n* = 685), respectively, which clearly illustrate that CMS4 CRCs were preferentially associated with both higher 13-gene MEKi bypass and higher SRC activation scores (see the “CMS4” panel, the “right and upper” quadrant). (**c**) The 18-gene MEK activation versus the 5-gene Dasa-S signature scores were plotted in each of the CMS1-4 subtypes (*n* = 422 Moffitt CRC tumors with the mutation status of *KRAS/NRAS/BRAF). BRAF (V600E)* (blue); MUT *KRAS/NRAS* (red*);* WT *RAS/RAF* (gray). The 18-gene MEK activation versus the 5-gene Dasa-S signature scores were plotted in each of the CMS1-4 subtypes in (**d**) Marisa 458 CMS1-4 CRC tumors with MUT and WT *KRAS/BRAF* data and (**e**) Medico 113 CMS1-4 CRC cell lines with MUT and WT *KRAS/BRAF* data. These data suggest problematic RAS-mutant CMS4 stem-like tumors may be sensitive to the novel drug combination of a SRCi + MEKi. **f.** A proposed model illustrates a central role of SRC in mediating resistance to MEK inhibition in mesenchymal-like cancer stem cells. SRC may serve as a common targetable node, suggesting potential for a new biomarker-driven (MEKi + SRCi) drug combination targeting problematic SRC-mediated, mesenchymal CSCs, especially *KRAS*-mutant CMS4 CRCs

The strong association of the 13-gene MEKi-resistance score with the SRC activation score was shown in Moffitt 2012 CMS1-4 CRC tumors (Fig. 8a). When scatter plots of 13-gene MEKi resistance vs SRC activation scores were made in each of individual CMS1-4 classes, it is clearly seen that CMS4 tumors were associated with the highest 13-gene resistance and SRC activation scores **(**see Fig. 8b “CMS4” panel, RUQ), suggesting strong association of CMS4 tumors with SRC activation and MEKi resistance. In addition, the 18-gene MEK activation versus the 5-gene Dasa-S signature scores were plotted in 422 CMS1-4 tumors with the mutation status of *KRAS/NRAS/BRAF/APC/TP53* (from 468 CRCs with both targeted sequencing and global gene expression data [3]). The remarkably-distinct associations of MUT vs WT RAS/RAF tumors with the CMS1-4 subtypes were observed (Fig. 8c). Note that no distinct association of MUT vs WT *APC*/*TP53* tumors with the CMS subtypes was seen (see Additional File 3_Fig S9). These data reveal that CMS1 tumors with *BRAF* (V600E) and CMS4 tumors with *KRAS/NRAS* mutations were preferentially associated with both higher 18-gene MEK activation (potential MEKi sensitivity) and higher 5-gene dasatinib sensitivity scores (see Fig. 8c “CMS1” and “CMS4” panels, **“**right and upper” quadrants (RUQs)), suggesting these tumors may be likely responders to a combination therapy of MEKi + SRCi in CRC. Similar results were seen in Marisa 458 CMS1-4 CRC tumors (Fig. 8d). Notably, the finding is also supported by the analysis of Medico 113 CMS1-4 CRC cell lines (Fig. 8e) among which HCT116 (CMS4, *KRAS* G13D) and LIM2405 (CMS1, *BRAF* (V600E) (both within Fig. 8e RUQs) were shown to be sensitive to MEKi + SRCi in vitro (Additional File 3_Fig S10 and S11). HCT116, LIM2405 or HT29 cells grown in CSC media (vs non-CSC media) showed morphologically more mesenchymal-like or large colony sizes and displayed greater levels of MEKi (trametinib)-resistance, which was attenuated by SRCi (dasatinib) (Fig S10 and S11). In agreeing with this, a gene expression signature correlation analysis in Medico CRC cell lines (*n* = 154) shows a strong correlation of MEKi-resistance with EMT, SRC activation and dasatinib sensitivity (see Fig S10a).

## Discussion

MEK is a canonical member of the RAS pathway and has been targeted for the development of therapeutic inhibitors. However, MEK inhibitors, used alone, have been largely ineffective, likely due to intrinsic and adaptive resistance mechanisms [5–7]. These complex resistance mechanisms are poorly understood. Here, our extensive gene expression signature analyses in human CRCs have revealed a novel, dominant biological feature in the MEKi-resistance mechanisms where SRC plays a central role and serves as a potential “Achilles’ heel”. Importantly, our data have identified subpopulations of RAS-activated CRC tumors that may be sensitive to combination of MEKi + SRCi, which has yet to be clinically investigated in CRC.

Our gene expression signature analyses in 2250 Moffitt CRC tumors, and in two independent CRC tumor datasets (Marisa (*n* = 585) [23], TCGA (*n* = 677) [47]) as well as in Medico CRC cell lines (*n* = 154) [24], revealed that the 13-gene MEKi “bypass”-resistance signature score was strikingly-correlated with SRC activation, as measured by two independently reported signatures measuring SRC activation/dependency [27, 28] (Figs. 2,6,7,S10a). SRC activation was associated *clinically* with stage I-IV progression and metastasis, especially regional metastasis, primary disease recurrence and poor overall survival (Fig. 2). In support of this, SRC activation was strongly correlated with the PC1 and EMT signatures and the EMT genes (e.g. *ZEB2*, *TWIST1*) (Figs. 3,S7,S8) that are known to promote migration, invasion and metastasis and to induce tumor cells to acquire stem cell characteristics [41–43]. Note that we previously reported the PC1 signature predicted disease progression and recurrence in CRC [25] whereas *TWIST1* overexpression was reported to be associated with nodal invasion (regional metastasis) in primary colorectal cancer [48]. At the same time, it was intriguing to see that a subset of EMT genes (*SNAIL2, ZEB1, ZEB2, TWIST2*) were specifically repressed in distant metastases, and thus may be linked to MET (the reverse process of EMT) (Fig S2). While EMT promotes cancer cell motility and dissemination, MET is thought to enhance metastatic colonization at distant sites [41–43]. Moreover, both MEKi resistance and SRC activation signatures were only modestly correlated with the humanized ISC signatures that reported to identify CRC stem cells and predict disease relapse [29] (Fig. 4). However, further analyses indicate that both MEKi-resistance and SRC activation were preferentially associated with tumors characterized by mesenchymal CSC (the EMT-like stemness) (Fig. 4). These data support a central role of SRC in mediating *intrinsic* and *adaptive* “bypass”-resistance to MEK inhibition in mesenchymal CSC-like CRCs.

RAS pathway AR has been a recent major focus in targeted therapies [8–12]. While great progress has been made in understanding the AR in response to BRAFi [8–11], much less is known about MEKi-induced AR mechanisms which appear to act through more diverse “bypass” signaling pathways. For example, while MEKi (selumetinib, AZD6244) may mediate feedback activation of EGFR in *BRAF* (V600E) CRC cells [9], no clinical responses were observed in CRC patients treated with combination of MEKi with EGFRi [10]. This suggested that, in contrast to that observed with BRAFi treatments [9, 11], MEKi may mediate AR by a mechanism independent of EGFR. This notion is supported by the observation that MEKi (selumtinib) induced MYC-dependent transcriptional upregulation of the ERBB3 receptor tyrosine kinase in *KRAS*-mutated CRC cell lines [7]. It is anticipated that MEKi may also induce feedback activation of additional RTKs in CRCs due to their associated genetic heterogeneity. In addition, it is formally possible that these RTKs may also directly contribute to intrinsic resistance mechanisms to MEK inhibitors [5]. Since it is practically difficult to develop and deliver multiple, customized therapeutic inhibitors for a large variety of RTKs that could be involved in AR, we have hypothesized that targeting a common signaling node channeling multiple paths to MEKi resistance could be effective. The SRC oncogene is a well-studied non-receptor tyrosine kinase [16–19]. In response signaling from a variety of RTKs (including EGFR, HER2, HER4, PDGFR, VEGFR, FGFR4, GPCR, C-MET, IL-4/IL13/IL-13Rα2, IL-6ST/IL-6R/IL-11R), SRC has been reported to mediate diverse cell signaling pathways including RAS/MAPK (proliferation), PI3K/AKT (survival), FAK (adhesion/migration/EMT), and STAT3 (angiogenesis) [17, 49, 50]. These RTKs and signaling pathways may all possibly contribute to the development of adaptive resistance to MEKi. We have hypothesized that inhibition of SRC may block the induction of AR in a significant subpopulation of CRC with activated SRC, that may represent the drug-resistant CSC. In addition, SRC was shown to be activated downstream of Wnt signaling and was required for tumorigenesis after *APC* loss [44]. Recently, MEK inhibitors were reported to activate Wnt signaling and induce stem cell plasticity in CRC in vitro [51]. Therefore, for the first time, we propose a novel role for SRC as a common targetable node in MEKi resistance mechanisms of CRC, permitting effective cancer stem cell targeting (Fig. 8f).

Since its first description in 2015, CMS classification [46] has been extensively explored for its clinical significance in predicting CRC prognosis and treatment outcomes [52]. We have applied CMS classification system to over 2000 Moffitt CRC tumors, as well as two independent CRC tumors datasets and one CRC cell line dataset, for subsequent signature correlation analyses (Figs. 5–8,S7,S8). We found that both poorly-differentiated CMS4 (mesenchymal, stem cell subtype) and CMS1 (MSI, immune subtype) tumors [52] were associated with SRC-mediated MEKi resistance. Notably, CMS4 tumors were strongly associated with SRC-mediated mesenchymal CSCs. Intriguingly, CMS1 tumors had the highest 18-gene MEK pathway activation scores and the second highest scores in 13-gene MEKi bypass-resistance, SRC activation, 5-gene Dasa-S and PC1 signatures but had the lowest Hu-Lgr5-ISC, and Hu-EphB2-ISC scores (Figs. 5–7), suggesting the association of CMS1 tumors with MEKi-resistance via a distinct mechanism from CMS4 tumors.

Notably, exploration of drug combinations of MEK and SRC inhibitors have been carried out in vitro using various types of cancer cell lines and to a less degree in vivo using mouse models, where enhanced anti-cancer effects have been reported [53–57]. However, it is known that the promise of targeted therapeutic agents supported by various cell line and/or animal studies has been often tempered by disappointing clinical activity, as shown in many clinical trials with unselected patients. Thus, development of rational combination therapies based on a deep understanding of targetable subpopulations of heterogeneous human tumors is still an unmet need. Our data suggest that *KRAS/NRAS*-mutated CMS4 tumors, more so than *BRAF-mutated* (typically CMS1) tumors, were preferentially associated with the highest 18-gene MEK activation and 5-gene Dasa-S scores (Fig. 8c-e, **RUQs**). This suggests that these tumor subpopulations may be most sensitive to a combination therapy of trametinib (MEKi) and dasatinib (SRCi), two FDA-approved targeted agents for other cancer treatments. While neither drug has been very effective as a single agent, their combinatorial effect on biomarker-selected subpopulations has yet to be explored in CRC. Currently, no good therapeutic option is available for CRC patients harboring *RAS* mutations.

The limitations of our study are the retrospective nature of primarily in silico bioinformatic analysis with the use of gene expression signature scores as a surrogate for MEKi or SRCi sensitivity due to the paucity of available human tumor data from CRC patients treated with MEKi or SRCi therapy. Moreover, the combination of MEKi + SRCi has never been tested in CRC patients. While our cell line data (Fig S10 and S11) are limited, they are supportive. To justify a future prospective clinical trial, more extensive experimental studies using heterogenous CRC cell lines and PDX models may be needed to preclinically validate our analysis suggesting that problematic KRAS/NRAS-mutated CMS4 CRC tumors may be sensitive to combined MEKi + SRCi therapy.

## Conclusions

Our compelling findings, derived directly from a comprehensive, multi-signature pathway signaling analysis of thousands of CRC tumors, have suggested a mechanism and means to subvert AR (or intrinsic resistance) in the highly drug-resistant CSC, by inhibiting a common SRC signaling node. Here, for the first time, these robust human data support a scientific rationale for the “fast-track” development of a novel biomarker-driven drug combination (MEKi + SRCi) to treat drug-resistant subpopulations of CMS4 patients harboring *KRAS/NRAS* mutations (42–44%, Fig. 8c,d).

## Supplementary Information

**Additional file1.** Supplementary Methods_Table S1 Gene Lists of 11Signatures.pdf.

**Additional file2.** Table S1 Gene Lists of 11 Signatures.gmt.

**Additional file3.** Fig S1-S11.pdf. **Fig. S1.**The 5-gene dasatinib sensitivity (Dasa-S) signature score predicted the trendof dasatinib sensitivity in multiple CRC cell lines (*n*=50). **Fig.S2.** The EMT genes were strongly associated with regionalmetastasis and disease recurrence in 2202 CRC tumors. **Fig. S3.**The CMS4 subtype was strongly correlated with the 13-gene MEKi“bypass”-resistance (13-gene BP), PC1, EMT, SRC activation and 5-gene Dasa-Ssignature scores in 1485 primary CRC tumors. **Fig.S4.** The CMS4 subtype was strongly correlated with the 13-geneMEKi “bypass”-resistance (13-gene BP), PC1, EMT, SRC activation and 5-geneDasa-S signature scores in 764 metastatic CRC tumors. **Fig. S5.**Scatter plots of Hu-Lgr5-ISC, Hu-EphB2-ISC, Hu-Late TA, and Hu-Proliferation vsEMT signature scores, respectively in Marisa 585 CRCs. **Fig. S6.**Scatter plots of Hu-Lgr5-ISC, Hu-EphB2-ISC, Hu-Late TA, and Hu-Proliferation vsEMT signature scores in TCGA 677 CRCs. **Fig. S7.**Spearman correlation heatmap of CMS1-4* scores, signature scores andEMT-associated genes in Marisa 585 CRC tumors. **Fig.S8.** Spearman correlation heatmap of CMS1-4* scores, signaturescores and EMT-associated genes in TCGA 677 CRC tumors. **Fig. S9.** Nodistinct association of MUT vs WT APC/TP53 tumors with the CMS subtypes. **Fig. S10**. Correlationanalysis of 154 CRC cell lines and in vitro drug treatment of HCT116 cells withMEKi + SRCi in CSC vs. non-CSC media. **Fig.S11**. In vitro drug treatment of LIM2405and HT29 cells with MEKi + SRCi in CSC vs. non-CSC media.

**Additional file4.** Moffitt 2250 CRC_CMS_signature scores.xls.

**Additional file5.** Marisa 585 CRC_CMS_signature scores.xls.

**Additional file6.** TCGA 677 CRC_CMS_signature scores.xls.

**Additional file7.** 154 CRC cell lines_CMS_signaturescores.xls.

**Additional file8.** Source data for Fig S10_S11 blots.docx.

## Data Availability

The complete data of the CMS subtypes and 11 signature scores of Moffitt 2250 CRC tumors, Marisa 585 CRC tumors and TCGA 677 CRC tumors and Medico 154 CRC cell lines supporting the findings of this paper are available as supplementary EXCEL files (Additional Files 4–7). Public access is open for the databases of Marisa CRCs, TCGA CRCs and Medico cell lines via the GEO accession numbers and the Broad GDAC Firehose link as described below. Note that due to the policy restriction of the co-owner institution (Merck), the raw gene expression data of Moffitt 2250 CRC tumors are not available for deposition. However, the precise same methodologies/algorithms used to assess the Moffitt 2250 CRCs were used to assess three independent datasets that are publicly-available and can be reproduced. We have successfully validated the findings from Moffitt 2250 CRCs using these three independent CRC datasets: **(1)**Marisa CRC tumors (*n* = 585)–-Affymetrix gene expression data of these samples are available via GEO with accession number GSE39582;(2) TCGA CRC dataset (*n* = 677).the TCGA COADREAD RNAseq Level 3 RSEM genes normalized data are available from the Broad GDAC Firehose (https://gdac.broadinstitute.org/runs/stddata__2016_01_28/data/COAD/20160128/). Note that from the parental directory on the link, select and download two tar.gz files with names (1.gdac.broadinstitute.org_COAD.Merge_rnaseqv2__illuminahiseq_rnaseqv2__unc_edu__Level_3__RSEM_genes__data.Level_3.2016012800.0.0.tar.gzand 2.gdac.broadinstitute.org_COAD.Merge_rnaseqv2__illuminaga_rnaseqv2__unc_edu__Level_3__RSEM_genes_normalized__data.Level_3.2016012800.0.0.tar.gz). **(3) Medico et al. 154 CRC cell line dataset. Affymetrix gene expression data of these cell lines are available via GEO with accession number GSE59857. Thus, in lieu of full availability of Moffitt data, readers can check the methods used in this paper via these publicly available datasets.**

## Abbreviations

CRC: Colorectal cancer
CMS: Consensus molecular subtype
EMT: Epithelial to mesenchymal transition
PC1: The first principal component
CSC: Cancer stem cell
Moffitt: Moffitt Cancer Center
TCGA: The cancer genome atlas
MEKi: MEK inhibition/inhibitor
SRCi: SRC inhibition/inhibitor
BRAFi: BRAF inhibition/inhibitor
Dasa-S: Dasatinib sensitivity
AR: Adaptive resistance
BP: Bypass
KM: Kaplan Meier
OS: Overall survival
FFPE: Formalin-fixed, paraffin-embedded
Hu: Humanized

## References

[1] Schubbert S, Shannon K, Bollag G (2007). Hyperactive Ras in developmental disorders and cancer. Nat Rev Cancer.

[2] Cancer Genome Atlas N (2012). Comprehensive molecular characterization of human colon and rectal cancer. Nature.

[3] Schell MJ, Yang M, Teer JK, Lo FY, Madan A, Coppola D (2016). A multigene mutation classification of 468 colorectal cancers reveals a prognostic role for APC. Nat Commun.

[4] Schell MJ, Yang M, Missiaglia E, Delorenzi M, Soneson C, Yue B (2016). A Composite Gene Expression Signature Optimizes Prediction of Colorectal Cancer Metastasis and Outcome. Clin Cancer Res.

[5] Caunt CJ, Sale MJ, Smith PD, Cook SJ (2015). MEK1 and MEK2 inhibitors and cancer therapy: the long and winding road. Nat Rev Cancer.

[6] Dry JR, Pavey S, Pratilas CA, Harbron C, Runswick S, Hodgson D (2010). Transcriptional pathway signatures predict MEK addiction and response to selumetinib (AZD6244). Cancer Res.

[7] Sun C, Hobor S, Bertotti A, Zecchin D, Huang S, Galimi F (2014). Intrinsic resistance to MEK inhibition in KRAS mutant lung and colon cancer through transcriptional induction of ERBB3. Cell Rep.

[8] Yaeger R, Yao Z, Hyman DM, Hechtman JF, Vakiani E, Zhao H (2017). Mechanisms of Acquired Resistance to BRAF V600E Inhibition in Colon Cancers Converge on RAF Dimerization and Are Sensitive to Its Inhibition. Cancer Res.

[9] Prahallad A, Sun C, Huang S, Di Nicolantonio F, Salazar R, Zecchin D (2012). Unresponsiveness of colon cancer to BRAF(V600E) inhibition through feedback activation of EGFR. Nature.

[10] Corcoran RB, Andre T, Atreya CE, Schellens JHM, Yoshino T, Bendell JC (2018). Combined BRAF, EGFR, and MEK Inhibition in Patients with BRAF(V600E)-Mutant Colorectal Cancer. Cancer Discov.

[11] Corcoran RB, Ebi H, Turke AB, Coffee EM, Nishino M, Cogdill AP (2012). EGFR-mediated re-activation of MAPK signaling contributes to insensitivity of BRAF mutant colorectal cancers to RAF inhibition with vemurafenib. Cancer Discov.

[12] Chen G, Gao C, Gao X, Zhang DH, Kuan SF, Burns TF (2018). Wnt/beta-Catenin Pathway Activation Mediates Adaptive Resistance to BRAF Inhibition in Colorectal Cancer. Mol Cancer Ther.

[13] van Geel R, Tabernero J, Elez E, Bendell JC, Spreafico A, Schuler M (2017). A Phase Ib Dose-Escalation Study of Encorafenib and Cetuximab with or without Alpelisib in Metastatic BRAF-Mutant Colorectal Cancer. Cancer Discov.

[14] Bardelli A, Siena S (2010). Molecular mechanisms of resistance to cetuximab and panitumumab in colorectal cancer. J Clin Oncol.

[15] Canon J, Rex K, Saiki AY, Mohr C, Cooke K, Bagal D (2019). The clinical KRAS(G12C) inhibitor AMG 510 drives anti-tumour immunity. Nature.

[16] Courtneidge SA, Fumagalli S, Koegl M, Superti-Furga G, Twamley-Stein GM. The Src family of protein tyrosine kinases: regulation and functions. Dev Suppl. 1993:57–64 8049488

[17] Yeatman TJ (2004). A renaissance for SRC. Nat Rev Cancer.

[18] Bjorge JD, O’Connor TJ, Fujita DJ. Activation of human pp60c-src. Biochem Cell Biol. 1996;74(4):477–84. 10.1139/o96-052 8960354

[19] Ishizawar R, Parsons SJ (2004). c-Src and cooperating partners in human cancer. Cancer Cell.

[20] Irby RB, Mao W, Coppola D, Kang J, Loubeau JM, Trudeau W (1999). Activating SRC mutation in a subset of advanced human colon cancers. Nat Genet.

[21] Fenstermacher DA, Wenham RM, Rollison DE, Dalton WS (2011). Implementing personalized medicine in a cancer center. Cancer J.

[22] Yang M, Schell MJ, Loboda A, Nebozhyn M, Li J, Teer JK (2019). Repurposing EGFR Inhibitor Utility in Colorectal Cancer in Mutant APC and TP53 Subpopulations. Cancer Epidemiol Biomarkers Prev.

[23] Marisa L, de Reynies A, Duval A, Selves J, Gaub MP, Vescovo L (2013). Gene expression classification of colon cancer into molecular subtypes: characterization, validation, and prognostic value. PLoS Medicine.

[24] Medico E, Russo M, Picco G, Cancelliere C, Valtorta E, Corti G (2015). The molecular landscape of colorectal cancer cell lines unveils clinically actionable kinase targets. Nat Commun.

[25] Loboda A, Nebozhyn MV, Watters JW, Buser CA, Shaw PM, Huang PS (2011). EMT is the dominant program in human colon cancer. BMC Med Genomics.

[26] Herbst A, Jurinovic V, Krebs S, Thieme SE, Blum H, Goke B (2014). Comprehensive analysis of beta-catenin target genes in colorectal carcinoma cell lines with deregulated Wnt/beta-catenin signaling. BMC Genomics.

[27] Broecker F, Hardt C, Herwig R, Timmermann B, Kerick M, Wunderlich A (2016). Transcriptional signature induced by a metastasis-promoting c-Src mutant in a human breast cell line. FEBS J.

[28] Huang F, Reeves K, Han X, Fairchild C, Platero S, Wong TW (2007). Identification of candidate molecular markers predicting sensitivity in solid tumors to dasatinib: rationale for patient selection. Cancer Res.

[29] Merlos-Suarez A, Barriga FM, Jung P, Iglesias M, Cespedes MV, Rossell D (2011). The intestinal stem cell signature identifies colorectal cancer stem cells and predicts disease relapse. Cell Stem Cell.

[30] Arcaroli JJ, Touban BM, Tan AC, Varella-Garcia M, Powell RW, Eckhardt SG (2010). Gene array and fluorescence in situ hybridization biomarkers of activity of saracatinib (AZD0530), a Src inhibitor, in a preclinical model of colorectal cancer. Clin Cancer Res.

[31] Eide PW, Bruun J, Lothe RA, Sveen A (2017). CMScaller: an R package for consensus molecular subtyping of colorectal cancer pre-clinical models. Sci Rep.

[32] Loboda A, Nebozhyn M, Klinghoffer R, Frazier J, Chastain M, Arthur W (2010). A gene expression signature of RAS pathway dependence predicts response to PI3K and RAS pathway inhibitors and expands the population of RAS pathway activated tumors. BMC Med Genomics.

[33] Omolo B, Yang M, Lo FY, Schell MJ, Austin S, Howard K (2016). Adaptation of a RAS pathway activation signature from FF to FFPE tissues in colorectal cancer. BMC Med Genomics.

[34] Davis TB, Yang M, Schell MJ, Wang H, Ma L, Pledger WJ (2018). PTPRS Regulates Colorectal Cancer RAS Pathway Activity by Inactivating Erk and Preventing Its Nuclear Translocation. Sci Rep.

[35] Davis TB, Yang M, Wang H, Lee C, Yeatman TJ, Pledger WJ (2019). PTPRS drives adaptive resistance to MEK/ERK inhibitors through SRC. Oncotarget.

[36] Baumgartner M, Radziwill G, Lorger M, Weiss A, Moelling K (2008). c-Src-mediated epithelial cell migration and invasion regulated by PDZ binding site. Mol Cell Biol.

[37] Bolen JB, Veillette A, Schwartz AM, Deseau V, Rosen N (1987). Analysis of pp60c-src in human colon carcinoma and normal human colon mucosal cells. Oncogene Res.

[38] Cartwright CA, Meisler AI, Eckhart W (1990). Activation of the pp60c-src protein kinase is an early event in colonic carcinogenesis. Proc Natl Acad Sci U S A.

[39] Mao W, Irby R, Coppola D, Fu L, Wloch M, Turner J (1997). Activation of c-Src by receptor tyrosine kinases in human colon cancer cells with high metastatic potential. Oncogene.

[40] Talamonti MS, Roh MS, Curley SA, Gallick GE (1993). Increase in activity and level of pp60c-src in progressive stages of human colorectal cancer. J Clin Invest.

[41] Nieto MA, Huang RY, Jackson RA, Thiery JP (2016). Emt: 2016. Cell.

[42] Vanharanta S, Massague J (2013). Origins of metastatic traits. Cancer Cell.

[43] Shibue T, Weinberg RA (2017). EMT, CSCs, and drug resistance: the mechanistic link and clinical implications. Nat Rev Clin Oncol.

[44] Cordero JB, Ridgway RA, Valeri N, Nixon C, Frame MC, Muller WJ (2014). c-Src drives intestinal regeneration and transformation. EMBO J.

[45] Batlle E, Clevers H (2017). Cancer stem cells revisited. Nat Med.

[46] Guinney J, Dienstmann R, Wang X, de Reynies A, Schlicker A, Soneson C (2015). The consensus molecular subtypes of colorectal cancer. Nat Med.

[47] Grossman RL, Heath AP, Ferretti V, Varmus HE, Lowy DR, Kibbe WA (2016). Toward a Shared Vision for Cancer Genomic Data. N Engl J Med.

[48] Valdes-Mora F, Gomez del Pulgar T, Bandres E, Cejas P, Ramirez de Molina A, Perez-Palacios R (2009). TWIST1 overexpression is associated with nodal invasion and male sex in primary colorectal cancer. Ann Surg Oncol.

[49] Elsberger B (2014). Translational evidence on the role of Src kinase and activated Src kinase in invasive breast cancer. Crit Rev Oncol Hematol.

[50] Jin W (2020). Regulation of Src Family Kinases during Colorectal Cancer Development and Its Clinical Implications. Cancers (Basel).

[51] Zhan T, Ambrosi G, Wandmacher AM, Rauscher B, Betge J, Rindtorff N (2019). MEK inhibitors activate Wnt signalling and induce stem cell plasticity in colorectal cancer. Nat Commun.

[52] Sawayama H, Miyamoto Y, Ogawa K, Yoshida N, Baba H (2020). Investigation of colorectal cancer in accordance with consensus molecular subtype classification. Ann Gastroenterol Surg.

[53] Anderson GR, Winter PS, Lin KH, Nussbaum DP, Cakir M, Stein EM (2017). A Landscape of Therapeutic Cooperativity in KRAS Mutant Cancers Reveals Principles for Controlling Tumor Evolution. Cell Rep.

[54] Rao G, Kim IK, Conforti F, Liu J, Zhang YW, Giaccone G (2018). Dasatinib sensitises KRAS-mutant cancer cells to mitogen-activated protein kinase kinase inhibitor via inhibition of TAZ activity. Eur J Cancer.

[55] Yuan M, Xu LF, Zhang J, Kong SY, Wu M, Lao YZ (2019). SRC and MEK Co-inhibition Synergistically Enhances the Anti-tumor Effect in Both Non-small-cell Lung Cancer (NSCLC) and Erlotinib-Resistant NSCLC. Front Oncol.

[56] Ferguson J, Arozarena I, Ehrhardt M, Wellbrock C (2013). Combination of MEK and SRC inhibition suppresses melanoma cell growth and invasion. Oncogene.

[57] Simpkins F, Jang K, Yoon H, Hew KE, Kim M, Azzam DJ (2018). Dual Src and MEK Inhibition Decreases Ovarian Cancer Growth and Targets Tumor Initiating Stem-Like Cells. Clin Cancer Res.

