# Supplementary material for: An integrative gene expression signature analysis identifies CMS4 KRAS-mutated colorectal cancers sensitive to combined MEK and SRC targeted therapy
Source: BMC Cancer. 2022 Mar 10;22:256. doi: 10.1186/s12885-022-09344-3 (PMC8908604; doi:10.1186/s12885-022-09344-3)
Supplement: Supplementary file 1 — Additional file1. Supplementary Methods_Table S1 Gene Lists of 11Signatures.pdf. [file 12885_2022_9344_MOESM1_ESM.pdf]

Supplementary Methods:

In Vitro CRC Cell Line Analyses

Cell Culture**:** HCT116, LIM2405 and HT29 CRC cell lines were obtained from ATCC and tested monthly for mycoplasma contamination with Sigma LookOut® Mycoplasma qPCR Detection Kit (Cat No. MP0040A-1KT). Cells were maintained in RPMI 1640 (Gibco) supplemented with 10% FBS and 1% penicillin and streptomycin.

The Cancer Stem Cell (CSC) Medium Versus the Non-CSC, Regular Control Medium (FBS): The CSC medium was composed as follows: DMEM/F12 (11320033 Thermo Fisher Scientific, Inc.) supplemented with 1X B27 (17504044 Thermo Fisher Scientific, Inc.), 50 ng/ml recombinant human EGF (11376454001 Sigma-Aldrich), and 20 ng/ml basic fibroblast growth factor (11123149001 Sigma-Aldrich). Regular FBS medium was composed of DMEM/F12 (Spheroids) or RMPI (11875085 Thermo Fisher) (Matrigel) and 10% FBS (A4766801 Thermo Fisher).

Spheroid Assay: To form spheroids in CSC vs non-CSC (FBS) media, HCT116 cells were seeded in 6-well plates with 5 x10^4 cells/well and grown for 7 days in DMEM/F12-methylcelluose medium contains 0.9% methyl cellulose (36718, Alfa Aesar), 29 mM $NaHCO_3$ (Sigma), 2 mM L-Glutamine (25030-081 Gibco), 1% MEM NEAA (11140-050 Gibco) and 1 mM Sodium Pyruvate (11360-070 Gibco) (modified from suppl ref (1)) plus (1) the CSC supplements (CSC medium) or (2) 10% FBS (non-CSC (FBS) medium). The spheroid culture was then treated with Trametinib (A3018 ApexBio) or Dasatinib (A3017 ApexBio). Cells were monitored and photographed for morphology. After 48 hr, cells were washed with PBS to collect cell pellets for further analyses.

Matrigel Assay: HCT116, LIM2405 and HT29 CRC cells were implanted in Matrigel growth factor reduced (354230 Corning) and seeded into 12-well culture plates at 6,000 cells/300 µl Matrigel per well. Following Matrigel polymerization, the cells were overlaid with CSC medium vs non-CSC, FBS medium. Every 3 days the medium was changed. The Matrigel culture was then treated with Trametinib (A3018 ApexBio) or Dasatinib (A3017 ApexBio). Cells were monitored and photographed for morphology. Cell Recovery Solution (354253 Corning) was used to dissolve cells from the Matrigel for harvesting to perform further analyses.

Western Blot Analysis: Cells were lysed in 1x RIPA buffer (9806 Cell Signaling) containing 10 mM PMSF, Protease Inhibitor Cocktail (M250 Amresco), Phosphatase Inhibitor Cocktail 2 (P5726 Millipore), and Phosphatase Inhibitor Cocktail 3 (P0044 Millipore). The LI-COR Odyssey® CLx Imaging System was used to image all immunoblots. Li-Cor secondary antibodies, Goat anti-Rabbit IRDye 680RD and Goat anti-Mouse IRDye 800CW, were used with the duplexed primary antibodies. Primary antibodies were obtained from Cell Signaling: Cleaved PARP (Rabbit 5625), PARP (Rabbit 9532), phospho-ERK1/2 T202/Y204 (4370), phospho-SRC Y419 (2101) and SRC (Mouse 2105), ERK (Rabbit Ab 4695) and β-ACTIN (Mouse 58169). Rabbit monoclonal to SOX9 [EPR14335] from Abcam (ab185230) was also used. Of note, Spheroid cultures or Matrigel cultures were grown in CSC vs non-CSC media for ~7 days followed by various drug treatment combinations for 2 or 3 days. In order to compare induction of apoptosis (by cleaved PARP/total PARP) and changes in cell signaling (by P-SRC/total SRC and P-ERK/total ERK) between CSC vs non-CSC media under the same experimental setting, we cut some of the blots according to anticipated protein sizes (kDa) prior to hybridization with different antibodies for Western blot analysis (see Additional File 8 for source data of blots).

CellTiter-Glo® 3D Cell Viability Assay (G9682 Promega) was performed on cells seeded at 4 $x10^3$ on a 96-well plate with either CSC or FBS medium on Matrigel. Cells were then treated with the various combinations of Trametinib (A3018 ApexBio) or Dasatinib (A3017 ApexBio). Luminescence was then measured using a Varioskan Lux (VL0000D0 Thermo Fisher Scientific).

Annexin V Apoptosis Assay was performed as described previously(2, 3).

Aldefluor Assay from Stemcell Technologies (01700) was accomplished following the protocol from Mele et al.(4). Cells were analyzed using a BD FACSAria II Cell Sorter. PI was used as previously stated to sort out dead cells. Gating was performed using a negative control sample for each condition with DEAB added. Each sample was gated individually according to its negative DEAB control.

Soft Agar Assay was used for analysis of anchorage independent growth. The assay was carried out in six-well TC plates with each well containing a 0.3% (w/v) agarose top layer and a 0.6% (w/v) agarose bottom layer. The

agarose layers were prepared with culture media (i.e. CSC vs non-CSC (FBS) media). 2000 HT29 cells were seeded in top layer. After two weeks, colonies were stained with by adding 200 μl of 10mg/ml Nitro blue tetrazolium chloride (VWR) to each well. After staining for overnight, colonies were machine-counted by the Odyssey® DLx Imaging System (Li-COR).

Supplementary References

1. Hattermann K, Held-Feindt J, Mentlein R. Spheroid confrontation assay: a simple method to monitor the three-dimensional migration of different cell types in vitro. Ann Anat. 2011;193(3):181-4.
2. Davis TB, Yang M, Wang H, Lee C, Yeatman TJ, Pledger WJ. PTPRS drives adaptive resistance to MEK/ERK inhibitors through SRC. Oncotarget. 2019;10(63):6768-80.
3. Fu W, Sharma SS, Ma L, Chu B, Bui MM, Reed D, et al. Apoptosis of osteosarcoma cultures by the combination of the cyclin-dependent kinase inhibitor SCH727965 and a heat shock protein 90 inhibitor. Cell Death Dis. 2013;4:e566.
4. Mele L, Liccardo D, Tirino V. Evaluation and Isolation of Cancer Stem Cells Using ALDH Activity Assay. Methods Mol Biol. 2018;1692:43-8.

Table S1. Gene Lists of 11 Signatures Used in This Study

**1. 18-gene MEK pathway activation signature (adopted from ref 6)**
ANKRD15 DUSP4 DUSP6 ELF1 ETV4 ETV5 FXYD5 LGALS3 LZTS1 MAP2K3 PHLDA1 PROS1 S100A6 SERPINB1 SLCO4A1 SPRY2 TRIB2 ZFP106
**2. 13-gene MEKi bypass resistance signature (adopted from ref 6)**
BASP1 CD274 CLU COL12A1 COL5A1 CRIM1 FZD2 G0S2 GPR176 IL6 LOX SERPINE1 STAC
**3. SRC activation signature (61 UP-regulated genes adopted from ref 27)**
ABLIM3 ADD1 ADH1B AKR1C1 AKR1C2 ALPP ANKHD1 APP ATOH8 ATP1A1 ATP2C1 ATP6V1D BEST2 C5orf42 CADM3 CCDC80 CFB CLASP2 COL12A1 COL28A1 COL8A1 CXCR4 CYP4B1 DCN DKK1 EIF4A2 FBN1 FBXO32 FLVCR2 FN1 FPR1 GALNTL2 GRB10 HAS2 HSPA8 KANK2 MAP1B MAP2 MME MMP2 MYBPC1 NAPRT1 NEDD9 NUMA1 PAPPA PAPPA-AS1 PARP9 PHF17 PLEC PSAP RPS24 RTN1 SERPINE2 SETD2 SETD5 SMARCA2 SRGN STAU1 TFPI WNT5A ZCCHC6
**4. 5-gene dasatinib sensitivity signature (adopted from ref 28)**
5 UP genes: EPHA2, CAV1, CAV2, ANXA1 and PTRF
**5. Hu-Lgr5-ISC signature (64 genes adopted from ref 29)**
ABTB2 AFAP1L1 APCDD1 ARHGEF4 ARNT2 AXIN2 BCL2 BEX1 BEX2 CAP2 CCDC46 CYP2E1 DGKG DLGAP1 DTL DYNC2H1 EPHA4 FAM64A FGFR4 FMNL2 FSTL1 GRAMD1A GRK4 IGF1R IGFBP4 IL17RD KIF12 KIF26B KLHL13 LDHB LGR5 LIFR LOC285141 MDFIC MPP3 NPNT PITPNC1 PLP1 RASSF4 RNF157 SCN2B SEPT6 SERTAD4 SLC1A2 SLC38A4 SLCO3A1 SLIT2 SOAT1 SORBS2 SOX4 TACC1 TMEM182 TNFRSF19 UTRN ZNF141 ZNF273 ZNF493 ZNF626 ZNF678 ZNF680 ZNF714 ZNF85 ZNF92 ZNF93
**6. Hu-EphB2-ISC signature (29 genes adopted from ref 29)**
ASRGL1 BCL2 BEX1 BEX2 CD44 CENPF CYP2E1 EPHB3 FMNL FSTL1 IGFBP4 KIF26B LGR5 MPP3 PRELP PSRC1 PTHLH PTPRO PVT1 SCN2B SLC1A2 SLC38A4 SLCO3A1 SOAT1 SORBS2 ST3GAL3 TACC1 TEAD2 TNFRSF19
**7. Hu-Late TA signature (51 genes adopted from ref 29)**
ACE2 AKR1B10 ALPI ANPEP CD36 CLIC5 CYP2C8 EGLN3 EMP1 ENPP3 F3 FMO5 FRMD3 GDA GDPD2 HKDC1 IGSF9 IL18 KRT20 LRRC19 MAFB MALL MGAT4C MME OAS3 OSGIN1 OSTalpha PAPSS2 PCK1 PFKP PLEKHG6 PTK6 RAB30 RHOC SLC13A1 SLC15A1 SLC23A1 SLC26A3 SLC26A6 SLC2A2 SLC2A5 SLC30A10 SLC5A12 SLC6A19 SLC9A3 SLITRK6 ST3GAL4 SYNPO TREH TRIM15 VWA1
**8. Hu-Proliferation signature (192 genes adopted from ref 29)**
ANLN AQP4 ASF1B ATAD2 ATIC ATR AURKA AURKB BAG2 BARD1 BBS7 BCL7A BLM BRCA1 BUB1 BUB1B C12orf48 C14orf106 C15orf23 C17orf53 C1orf112 C3orf26 C8orf79 CCDC14 CCDC99 CCNA2 CCNE1 CCNF CD55 CDC45L CDC6 CDC7 CDCA2 CDCA5 CELSR2 CENPE CENPF CENPI CENPN CEP192 CEP55 CHAF1B CHEK1 CHTF18 CKAP2L CLSPN CRTAC1 CTPS CYP39A1 DACH1 DCK DCTD DEPDC1 DIAPH3 DKC1 DNAH11 DNAJC18 DOCK4 DTL DUS4L ESCO2 EXO1 EXOSC2 EXOSC8 FBXL7 FEN1 FIGNL1 FOXM1 GEMIN4 GEMIN5 GINS1 GINS2 GINS3 GSTCD HELLS HEMGN HMGA2 HMMR IGF1R ILF3 INTU IQGAP3 KBTBD6 KCNN4 KIF11 KIF22 KIF4A KIFC1 LAMC1 LIG1 LPHN1 LYAR MBD4 MCM2 MCM3 MCM4 MCM5 MCM7 MCM8 MDN1 MELK MITF MLF1IP MPHOSPH9 MTHFD2 MYB MYBL2 MYC MYO5A NAP1L1 NCAPD2 NCAPG2 NCAPH NDE1 NEK2 NETO2 NEXN NUF2 NUP133 NUP85 NUSAP1 PAICS PALB2 PBK PBX3 PFAS PGM2L1 PLAGL1 PLK1 POLA1 POLD1 POLE2 POLI POLR1E PRKD3 PRR11 PSIP1 PUS7 QTRT1 RAD18 RAD51 RAD51AP1 RAD51C RAD54B RAD54L RASA3 RCC2 RFC3 RPA2 RPP40 RPUSD2 RRM1 RRM2 SACS SCML2 SGOL1 SGOL2 SIVA1 SLC12A2 SLC39A8 SLFN13 SMC2 SPAG5 TACC3 TBC1D19 TBC1D4 TCF19 TIMELESS TIMP3 TK1 TMEM107 TOPBP1 TPX2 TRAIP TSGA14 TSPAN12 TUBB TUBE1 UBE2T UHRF1 UNG UTP15 VRK1 WDHD1 WDR35 XRCC2 ZC3H7B ZNF275 ZNF367 ZNF473 ZNF704 ZNRF3
**9a. EMT UP signature (150 UP genes adopted from ref 22, 25)**
ADAM23 ADAMTS1 AFF3 AK5 AKAP12 ALPK2 ANGPTL2 ANKRD1 ANTXR1 ANXA6 AOX1 AP1S2 ARMCX1 ATP8B2 ATP8B3 AXL BDNF BICC1 BNC2 BVES C10orf38 C10orf56 C16orf45 C1S C9orf19 CAP2 CCL2 CDH11 CDH2 CDH4 CHN1 CLDN11 CLIP3 CMTM3 COL12A1 COL1A2 COL3A1 COL4A1 COL5A1 COL5A2 COL6A1 CPA4 CTGF CYBRD1 DAB2 DFNA5 DIO2 DKK3 DLC1 DOCK10 DPYSL3 EDIL3 ELOVL2 EML1 EMP3 EPB41L5 EPDR1 EVI2A F2R FAM101B FAT4 FBN1 FGF2 FGF5 FGFR1 FHL1 FLRT2 FSTL1 GFPT2 GLIPR1 GLT25D2 GNB4 GNG11 GPC6 GPR176 GREM1 HAS2 HEG1 HS3ST3A1 HTRA1 IGFBP7 IL13RA2 JAM3 KIRREL LAMA4 LEPREL1 LGALS1 LHFP LIX1L LOX MAP1B MMP2 MRAS MSRB3 NAP1L3 NAV3 NDN NEGR1 NEXN NID1 NRG1 NUDT11 PAPPA PDE7B PLAGL1 PMP22 PNMA2 POPDC3 POSTN PRKD1 PRR16 PTGIS PTRF PTX3 RBM24 RBMS3 RBPMS2 RECK RFTN1 SERPINE1 SIRPA SLC2A3 SLC47A1 SPARC SRGN SRPX ST3GAL2 SUSD5 SYDE1 TBXA2R TCF4 TGFB2 TMEM158 TMEM47 TMSL8 TNFRSF19 TRPA1 TTC28 TTLL7 TUB TUBA1A UCHL1 VIM WIPF1 WNT5B ZEB1 ZEB2 ZFPM2 ZNF788

**9b. EMT DOWN signature (162 DOWN genes adopted from ref 22, 25)**
ACPP AGR3 ALDH3B2 ANK3 ANKRD22 ANXA9 AP1M2 AQP3 ARHGAP8 ARHGDIB ATAD4 ATP2C2 B3GNT3 BLNK BSPRY C11orf52 C19orf21 C1orf106 C1orf116 C1orf210 C1orf34 CCDC64B CD24 CDH1 CDH3 CDS1 CEACAM5 CEACAM6 CGN CKMT1B CLDN4 CLDN7 CNKSR1 CNTNAP2 CTAGE4 DAPP1 DENND2D DMKN DSC2 DSP EHF ELF3 ELF5 EPN3 EPPK1 ERBB3 ERP27 FA2H FAAH2 FAM110C FAM83A FAM84A FAM84B FBP1 FGD2 FGFBP1 FUT1 FUT3 FXYD3 GALNT3 GCNT3 GJB6 GOLT1A GPR110 GPR87 GPX2 GRAMD2 GRHL1 GRHL2 HOOK1 HS3ST1 HS6ST2 IL1RN ILDR1 INPP4B IRF6 ITGB6 KLK10 KLK5 KLK6 KLK8 KRT16 KRT19 KRT5 KRTCAP3 LAMA3 LAMB3 LAMC2 LCN2 LCP1 LIPG MAL2 MAP7 MAPK13 MARVELD2 MARVELD3 MB MBNL3 MPP7 MUC20 MYH14 MYO5B NPNT OR2A4 OVOL2 PAK6 PKP3 PLA2G10 POU2F3 PPL PPP1R14C PROM2 PRR15 PRSS8 PTAFR PVRL4 RAB25 RAPGEF5 RASEF RASGEF1B RBM35A RBM35B S100A14 S100A8 S100A9 S100P SCEL SCNN1A SERPINB5 SH2D3A SH3YL1 SLC6A14 SLPI SORBS2 SPINK5 SPINT1 SPINT2 SPRR1A SPRR1B SPRR3 ST14 STEAP4 STX19 SYK SYT7 TMC4 TMC5 TMEM125 TMEM30B TMEM45B TMPRSS11E TMPRSS13 TMPRSS4 TOX3 TRIM29 TSPAN1 TTC22 TTC9 VTCN1 WDR72 WFDC2
**10a. PC1 UP signature (125 UP genes adopted from ref 22, 25)**
AEBP1 AKAP12 AKT3 AMOTL1 ANKRD6 AP1S2 ARMCX1 ARMCX2 ATP8B2 BASP1 BGN C1orf54 C20orf194 CALD1 CAP2 CCDC80 CEP170 CFH CFL2 CMTM3 COX7A1 CRYAB DCN DFNA5 DNAJB4 DPYSL3 DZIP1 ECM2 EFHA2 EFS EHD3 FAM20C FBXL7 FEZ1 FLRT2 FRMD6 GFPT2 GLIS2 GNG11 HECTD2 HTRA1 IL1R1 JAM3 KCNE4 KIAA1462 KLHL5 LAYN LDB2 LGALS1 LHFP LIX1L LMCD1 LPHN2 LZTS1 MAF MAGEH1 MAP1B MAP9 MCC MGP MLLT11 MPDZ MRAS MSN MSRB3 MXRA7 MYH10 MYO5A NAP1L3 NDN NNMT NR3C1 NRP1 NRP2 PEA15 PFTK1 PHLDB2 PKD2 PRICKLE1 PRKD1 PTPRM PTRF QKI RAB31 RAB34 RAI14 RASSF8 RECK RGS4 RNF180 SCHIP1 SDC2 SERPINF1 SGCE SGTB SLIT2 SMARCA1 SNAI2 SPARC SPG20 SRGAP2 SRPX STON1 SYT11 TCEA2 TCEAL3 TIMP2 TNS1 TPST1 TRPA1 TRPC1 TRPS1 TSPYL5 TTC7B TUBB6 TUSC3 UBE2E2 UCHL1 VIM WWTR1 ZFPM2 ZNF25 ZNF532 ZNF677
**10b. PC1 DOWN signature (120 DOWN genes adopted from ref 22, 25)**
ACOT11 AGMAT ANKS4B AP1M2 ATP10B AXIN2 BCAR3 BCL2L14 BDH1 BRI3BP C10orf99 C4orf19 C9orf152 C9orf75 C9orf82 CALML4 CAPN5 CASP5 CASP6 CBLC CC2D1A CCL28 CDC42EP5 CDS1 CDX1 CKMT1B CLDN3 CMTM4 CORO2A COX10 CYP2J2 DAZAP2 DDAH1 DTX2 DUOX2 DUOXA2 ENTPD5 EPB41L4B EPHB2 EPN3 EPS8L3 ESRRA ETHE1 EXPH5 F2RL1 FA2H FAM3D FAM83F FAM84A FRAT2 FUT2 FUT3 FUT4 FUT6 GALNT7 GMDS GPA33 GPR35 GPX2 HDHD3 HMGA1 HNF4A HOXB9 HSD11B2 KALRN KCNE3 KCNQ1 KIAA0152 LENG9 LGALS4 LRRC31 MAP7 MARVELD3 MCCC2 MPST MRPS35 MUC3B MYB MYH14 MYO5B MYO7B NAT2 NOB1 NOX1 NR1I2 PAQR8 PI4K2B PKP2 PLA2G12A PLEKHA6 PLS1 PMM2 POF1B PPP1R1B PREP RAB25 RNF186 SELENBP1 SH3RF2 SHH SLC12A2 SLC27A2 SLC29A2 SLC35A3 SLC37A1 SLC44A4 SLC5A1 SLC9A2 ST14 STRBP SUCLG2 SULT1B1 TJP3 TMC5 TMEM54 TMPRSS2 TST USP54,XK
**11. 64-gene WNT signature (64 UP β-catenin target genes adopted from ref 3, 26)**
ABCB1 ADAM10 ALEX1 ASCL2 AXIN2 BAMBI BCL2L2 BIRC5 BMI1 BMP4 CCND1 CD44 CDKN2A CDX1 CLDN1 COX2 DKK1 DKK4 DNMT1 EDN1 ENC1 EPHB2 EPHB3 FGF18 FGFBP FRA1 FSCN1 GAST HEF1(NEDD9) HES1 ID2 ITF2(TCF4) JAG1 JUN L1CAM LAMC2 LEF1 LGR5 MENA MET MMP14 MMP7 MYB MYC MYCBP NOS2 NOTCH2 NRCAM PLAU PLAUR PPARD S100A4 S100A6 SGK1 SMC3 SOX9 SP5 SRSF3(SRp20) SUZ12 TCF1 TIAM1 TN-C VEGF YAP

Note: all refs in Table S1 are from the main text references.
