# Supplementary material for: An integrative gene expression signature analysis identifies CMS4 KRAS-mutated colorectal cancers sensitive to combined MEK and SRC targeted therapy
Source: BMC Cancer. 2022 Mar 10;22:256. doi: 10.1186/s12885-022-09344-3 (PMC8908604; doi:10.1186/s12885-022-09344-3)
Supplement: Supplementary file 2 — Additional file2. Table S1 Gene Lists of 11 Signatures.gmt. [file 12885_2022_9344_MOESM2_ESM.pdf]

Supplementary Figures S1-S11

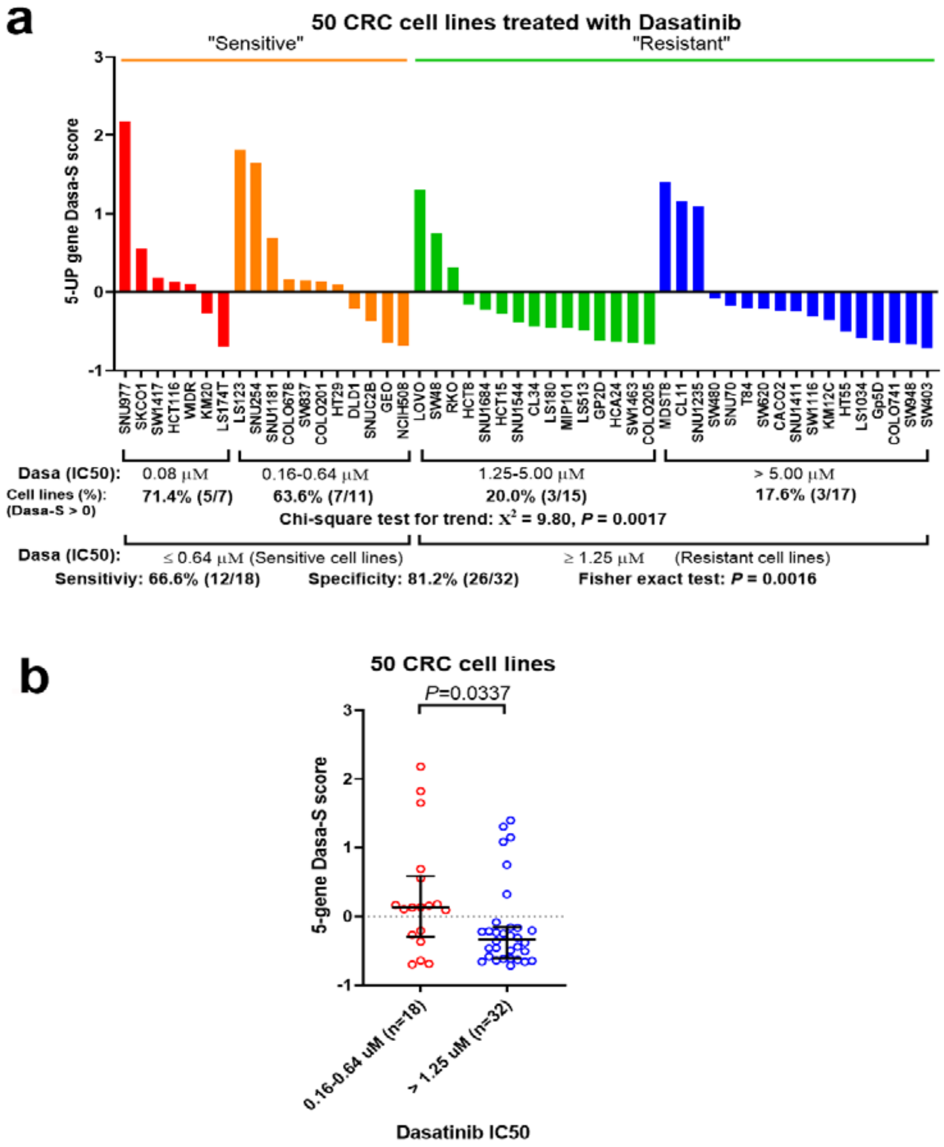

Fig S1. The 5-gene dasatinib sensitivity (Dasa-S) signature score predicted the trend of dasatinib sensitivity in multiple CRC cell lines (n=50). The 6-gene Dasa-S signature was developed in breast cell lines and validated in lung cell lines to predict sensitivity to dasatinib in solid tumors including breast, lung and ovary as reported by Huang et al.(1). Based on the 5 up-regulated genes of the 6-gene signature, we generated the 5-gene Dasa-S signature scores in 50 CRC cell lines (see **Methods**). For these cell lines we adopted the *in vitro* dasatinib treatment data from the analysis reported by Scott et al.(2) and the gene expression microarray data from the study of Medico et al.(3). (**a**) A waterfall plot of 5-gene Dasa-S score versus dasatinib IC50s in 50 CRC cell lines. Note that Huang et al(1) set 0.65 mM of dasatinib as the cut-off concentration for sensitive breast cancer cell lines (24 hr drug treatment) in the development of the gene classifiers of dasatinib sensitivity. Here the 5-gene Dasa-S scores are normalized by the median score that is set as 0. Chi-square test for trend

was performed for the 5-gene Dasa-S scores as to dasatinib IC50s: 0.08 vs 0.16-0.64 vs 1.25-5.00 vs >5.00 μM. Fisher exact test was also performed as to dasatinib IC50s ≤0.64 μM vs ≥1.25 μM. (**b**) Comparison of 5-gene Dasa-S signature scores between ≤0.64 μM vs ≥1.25 μM. Bars represent Median with interquartile range. *P* value is for two-tailed Mann Whitney test.

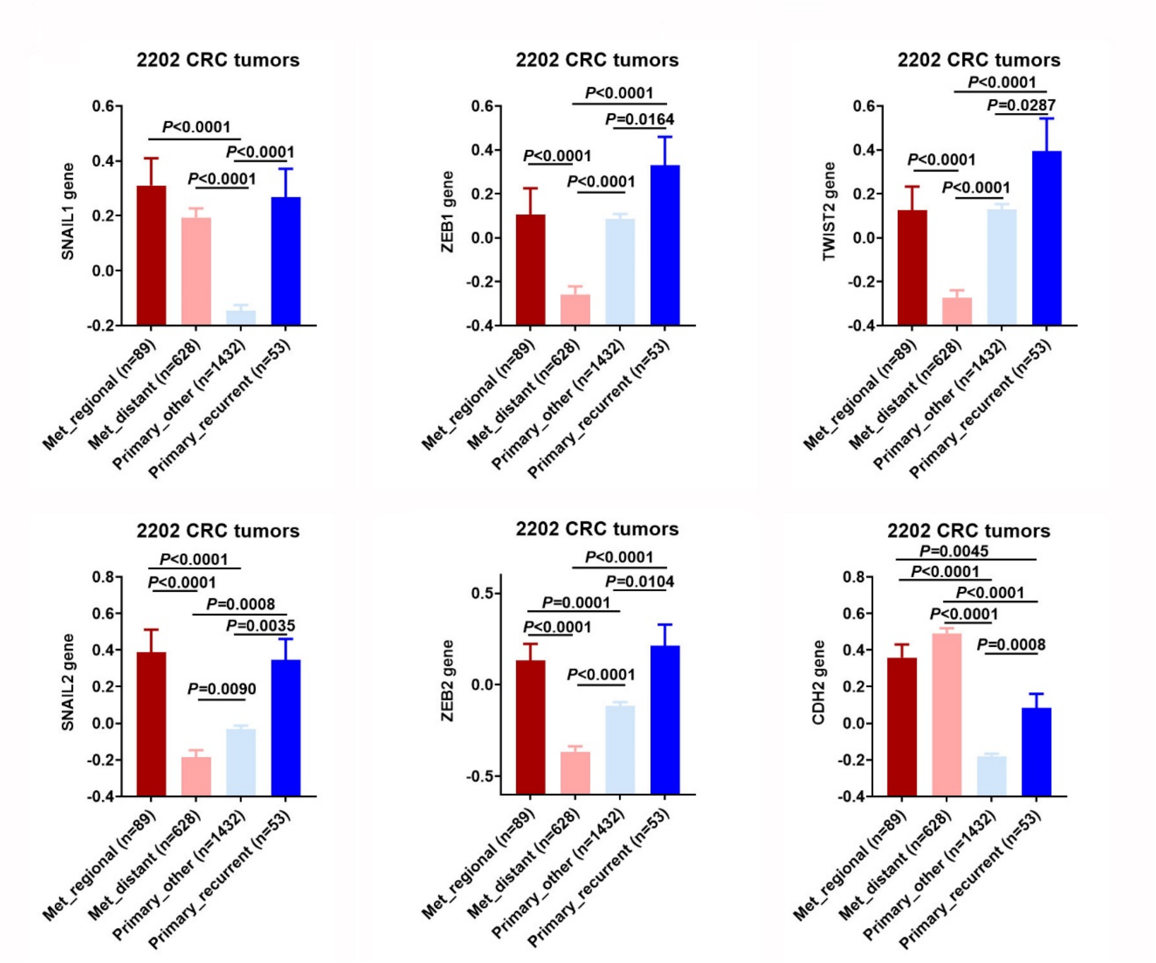

Fig S2. The EMT genes were strongly associated with regional metastasis and disease recurrence in 2202 CRC tumors. Comparison of expression of a set of well-known EMT genes among 2202 metastatic (regional, distant) vs primary tumors (recurrent, other). *TWIST1* gene is shown in **Fig 3j**. Note that among 2250 tumors, 48 tumors without approximate data were excluded from analysis. Bars represent Mean with standard errors (SEM). *P* values are for two-tailed Mann Whitney test. Note that the expression of *SNAIL2, ZEB1, ZEB2, TWIST2* was lower in in distant metastatic tumors (Met_distant, n=628) than other subgroups. EMT is known to promote cancer cell motility and dissemination and to induce tumor cells to acquire stem cell characteristics, whereas mesenchymal-to-epithelial transition (MET), the reverse process of EMT, is thought to enhance metastatic colonization at distant sites(4-8). These data suggest that suppression of the expression of these EMT genes may play a potential role in promoting MET in the distant metastasis.

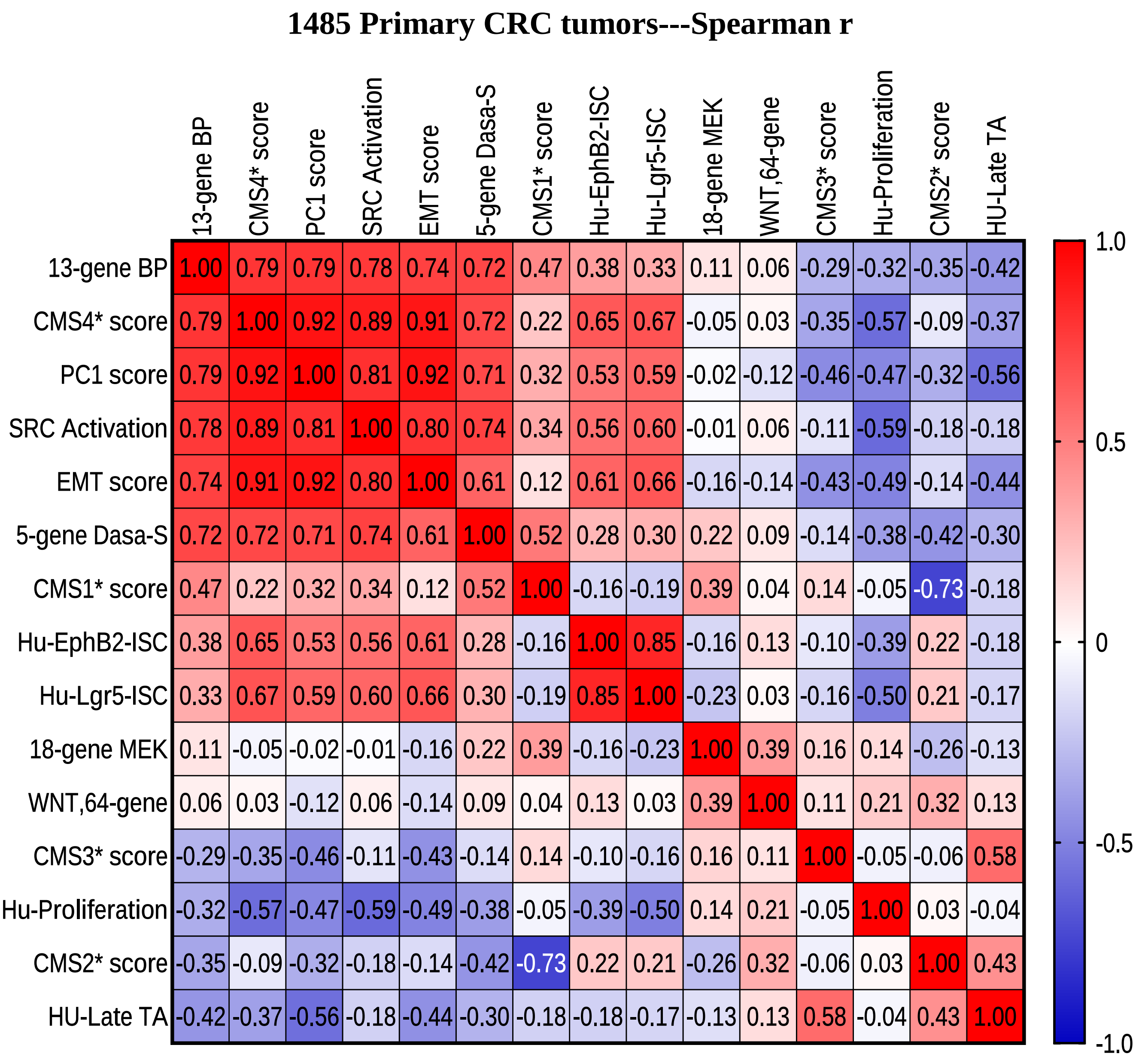

Fig S3. The CMS4 subtype was strongly correlated with the 13-gene MEKi "bypass"-resistance (13-gene BP), PC1, EMT, SRC activation and 5-gene Dasa-S signature scores in 1485 primary CRC tumors. Spearman correlation heatmap of the signature scores with CMS1-4* scores is shown, where CMS1*, CMS2*, CMS3* and CMS4* scores are designated to measure a propensity of a tumor to fall into CMS1, CMS2, CMS2 and CMS4 classes, respectively. 5-gene Dasa-S --- 5-gene dasatinib sensitivity; 18-gene MEK --- 18-gene MEK pathway activation.

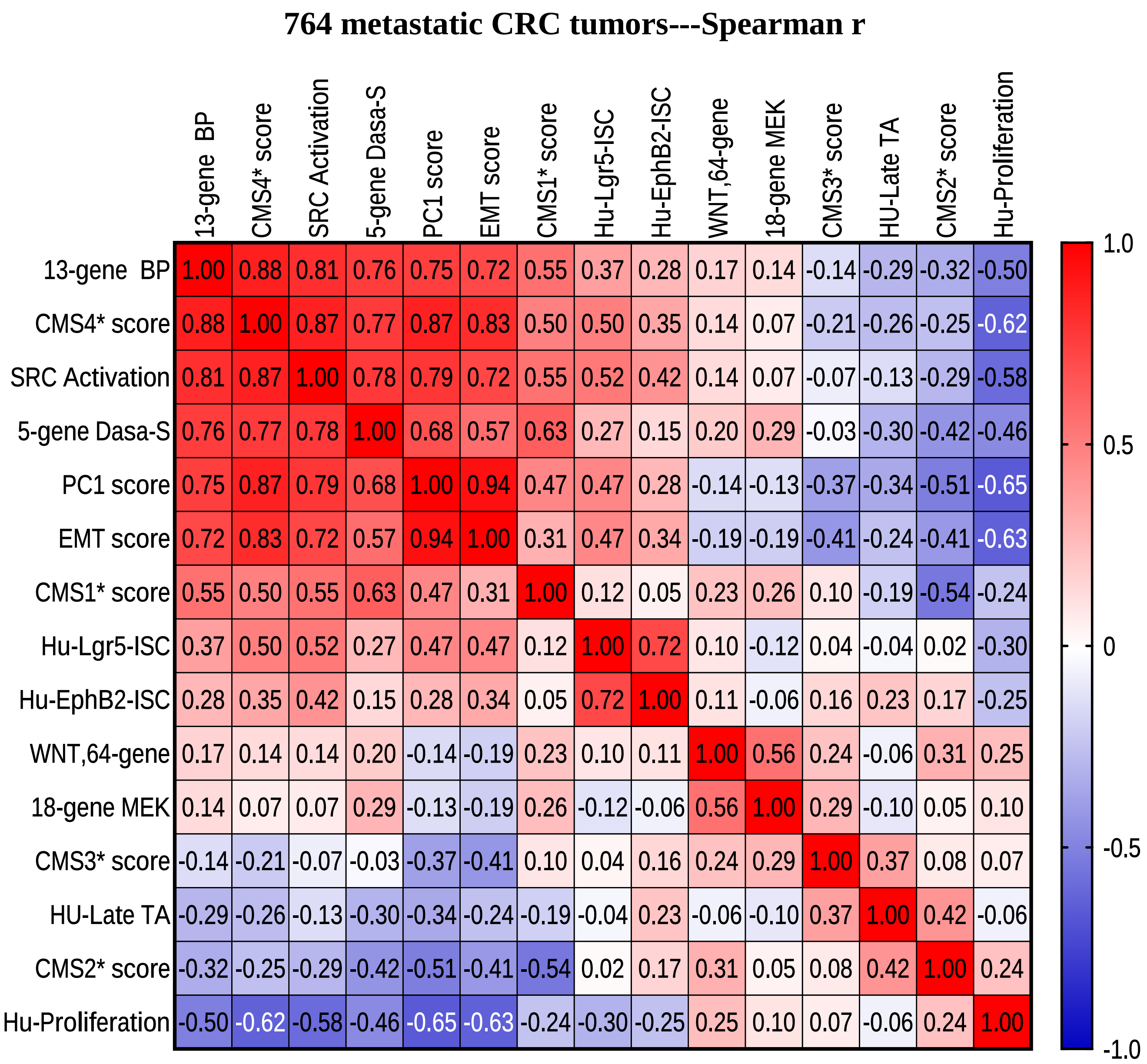

Fig S4. The CMS4 subtype was strongly correlated with the 13-gene MEKi "bypass"-resistance (13-gene BP), PC1, EMT, SRC activation and 5-gene Dasa-S signature scores in 764 metastatic CRC tumors. Spearman correlation heatmap of the signature scores with CMS1-4* scores is shown, where CMS1*, CMS2*, CMS3* and CMS4* scores are designated to measure a propensity of a tumor to fall into CMS1, CMS2, CMS2 and CMS4 classes, respectively. 5-gene Dasa-S --- 5-gene dasatinib sensitivity; 18-gene MEK --- 18-gene MEK pathway activation.

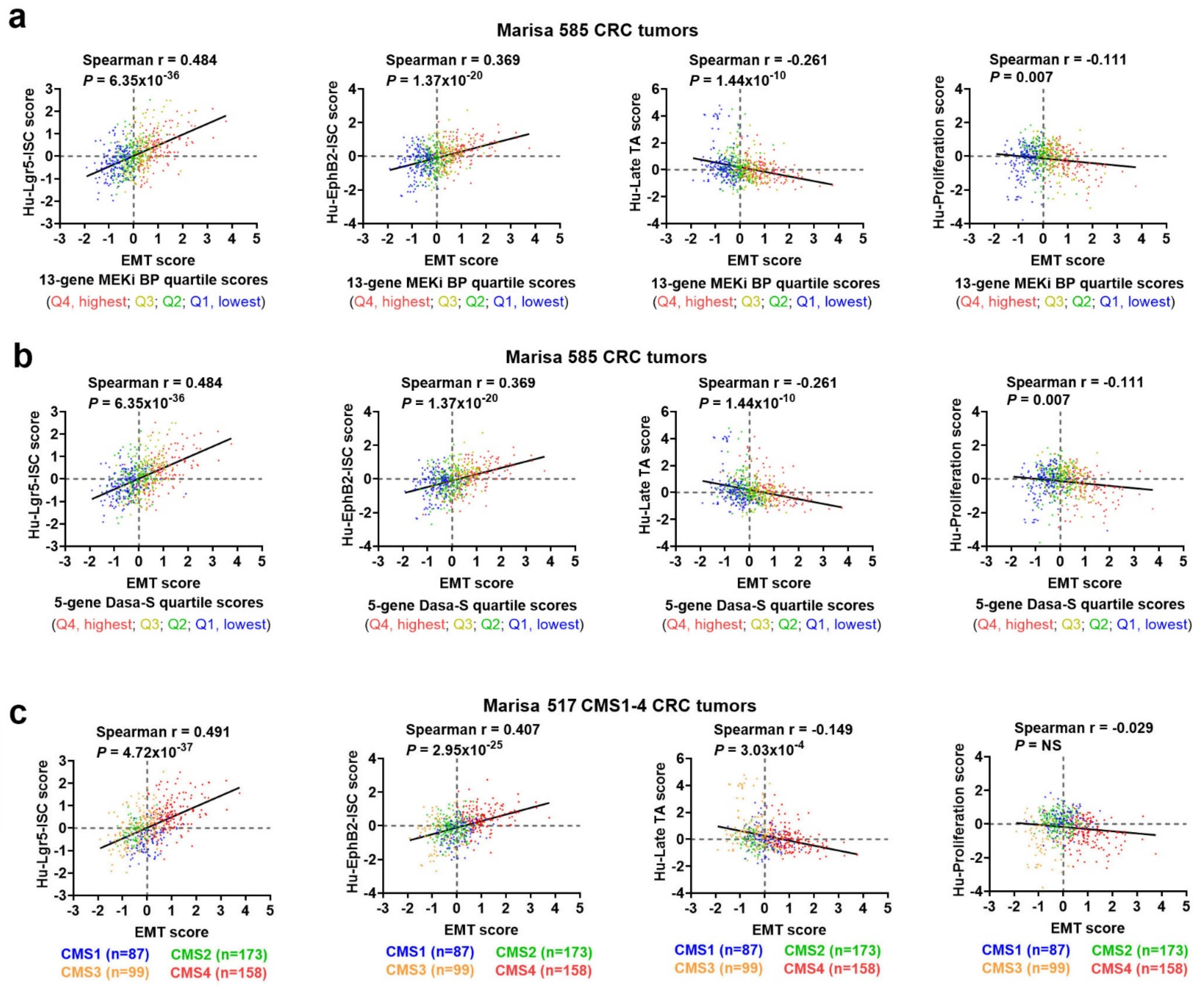

Fig S5. Scatter plots of Hu-Lgr5-ISC, Hu-EphB2-ISC, Hu-Late TA, and Hu-Proliferation vs EMT signature scores, respectively in Marisa 585 CRCs. The quartile scores (Q1-Q4) of (**a**) 13-gene MEKi BP and (**b**) 5-gene Dasa-S signatures are indicated by different colors (Q1, blue; Q2, green; Q3, yellow; Q4 red). (**c**) **Scatter plots of Hu-Lgr5-ISC, Hu-EphB2-ISC, Hu-Late TA, and Hu-Proliferation vs EMT signature scores in Marisa 517 CMS-14 CRCs**. The CMS1-4 subtypes are indicated by red (CMS4) vs orange (CMS3) vs green (CMS2) vs blue (CMS1) colors.

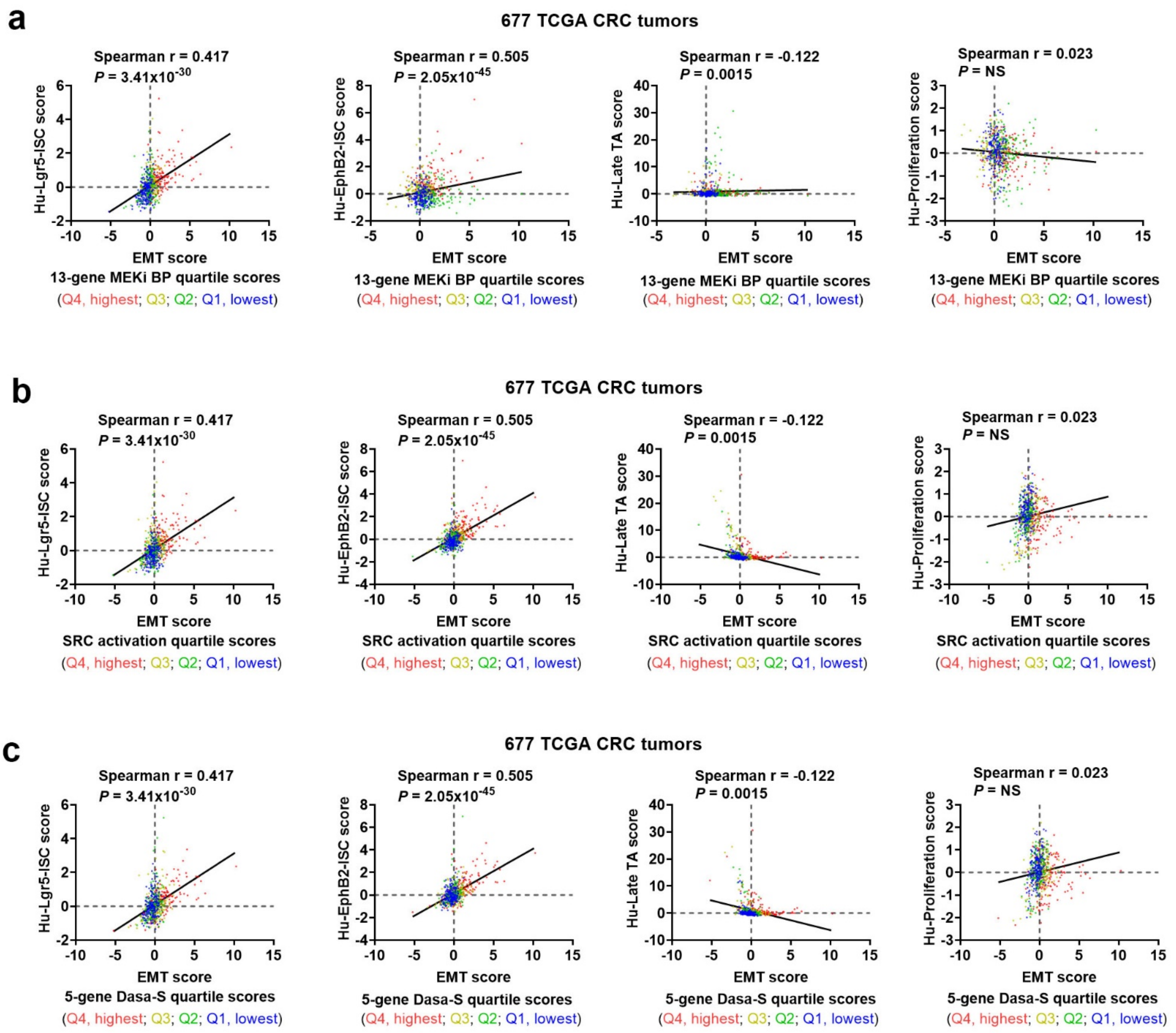

Fig S6. Scatter plots of Hu-Lgr5-ISC, Hu-EphB2-ISC, Hu-Late TA, and Hu-Proliferation vs EMT signature scores in TCGA 677 CRCs. The quartile scores (Q1-Q4) of (**a**) 13-gene MEKi BP, (**b**) SRC activation and (**c**) 5-gene Dasa-S signatures are indicated by different colors (Q1, blue; Q2, green; Q3, yellow; Q4 red).

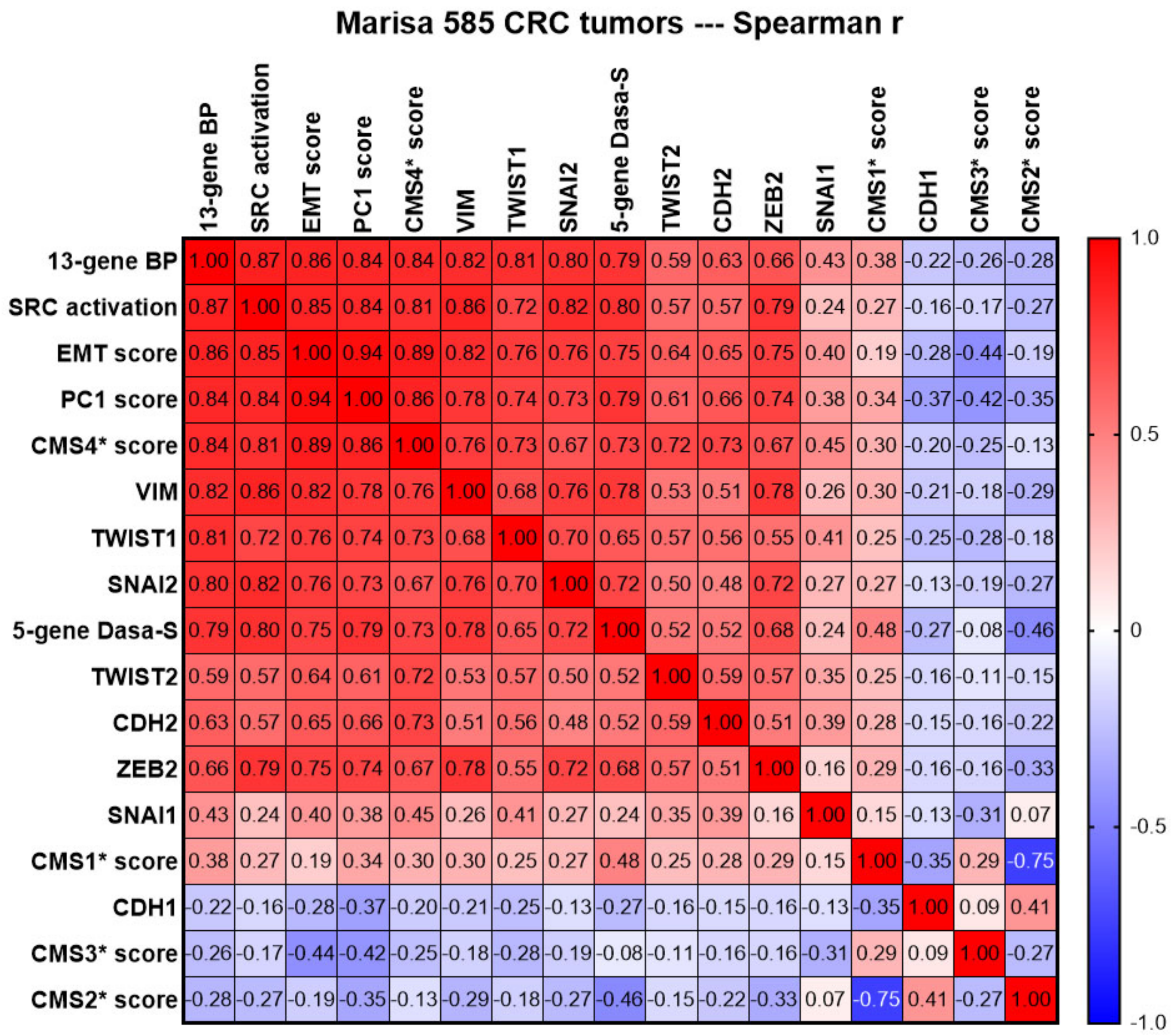

Fig S7. Spearman correlation heatmap of CMS1-4* scores, signature scores and EMT-associated genes in Marisa 585 CRC tumors. The 13-gene MEKi “bypass”-resistance (13-gene BP), PC1, EMT, SRC activation and 5-gene Dasa-S signature scores as well as CMS4 were correlated positively with expression of the mesenchymal marker *VIM* and other known EMT-associated genes including *TWIST1*, *SNAI2*, *TWIST2*, *CDH2*, *ZEB2*, *SNAI1* but negatively with the epithelial marker *CDH1*. Note that the Marisa dataset did not have approximate probe values of the ZEB1 gene. Here CMS1*, CMS2*, CMS3* and CMS4* scores are designated to measure a propensity of a tumor to fall into CMS1, CMS2, CMS2 and CMS4 classes, respectively. 5-gene Dasa-S --- 5-gene dasatinib sensitivity signature score.

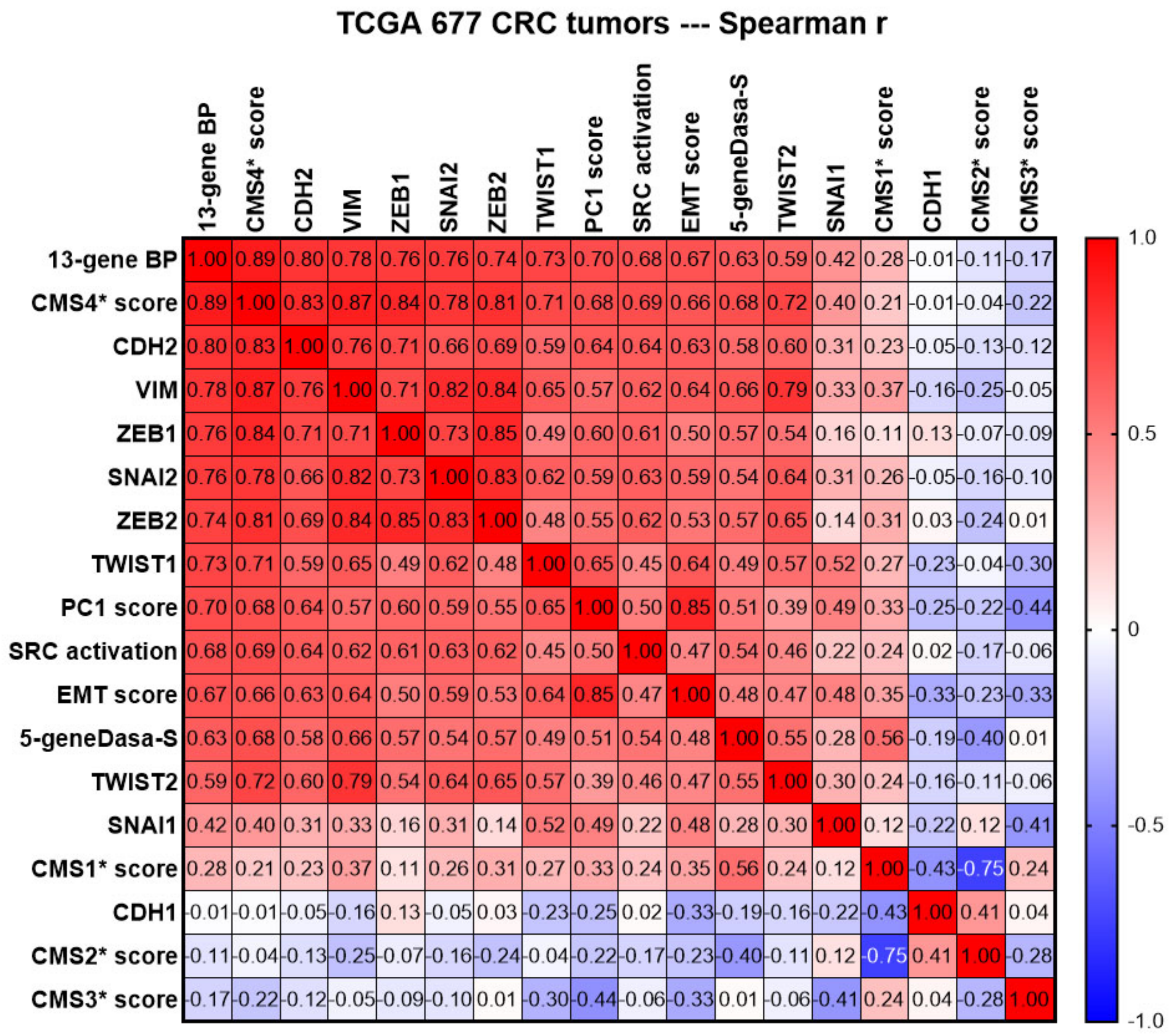

Fig S8. Spearman correlation heatmap of CMS1-4* scores, signature scores and EMT-associated genes in TCGA 677 CRC tumors. The 13-gene MEKi "bypass"-resistance (13-gene BP), PC1, EMT, SRC activation and 5-gene Dasa-S signature scores as well as CMS4 were correlated with expression of the mesenchymal marker *VIM* and other known EMT-associated genes including *CDH2*, *ZEB1*, *SNAI2*, *ZEB2*, *TWIST1*, *TWIST2*, and *SNAI1*. Here CMS1*, CMS2*, CMS3* and CMS4* scores are designated to measure a propensity of a tumor to fall into CMS1, CMS2, CMS2 and CMS4 classes, respectively. 5-gene Dasa-S --- 5-gene dasatinib sensitivity signature score.

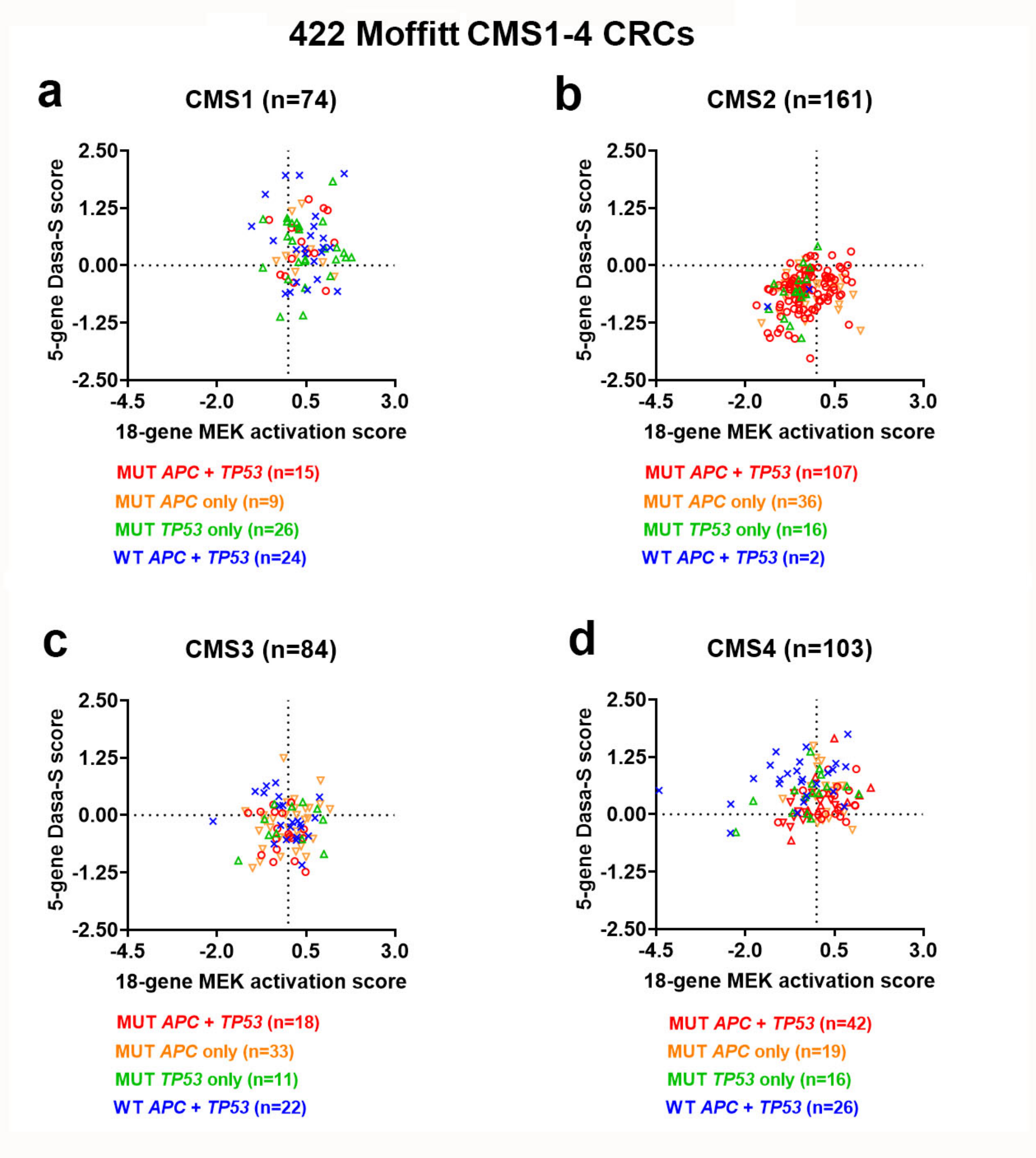

Fig S9. No distinct association of MUT vs WT *APC*/*TP53* tumors with the CMS subtypes. The 18-gene MEK activation versus the 5-gene Dasa-S signature scores were plotted in each of the CMS1-4 subtypes (n=422 Moffitt CRC tumors with the mutation status of *APC* and *TP53*. Four quadrants are defined by higher (>0 median) vs lower (<0, median) scores of the 18-gene and 5-gene signatures. MUT *APC* + *TP53* --- Both *APC* and *TP53* are mutated; MUT *APC* only--- *APC* mutated/*TP53* WT; MUT *TP53* only--- *APC* WT/*TP53* mutated; WT *APC* + *TP53* --- Both *APC* and *TP53* are wild-type (WT).

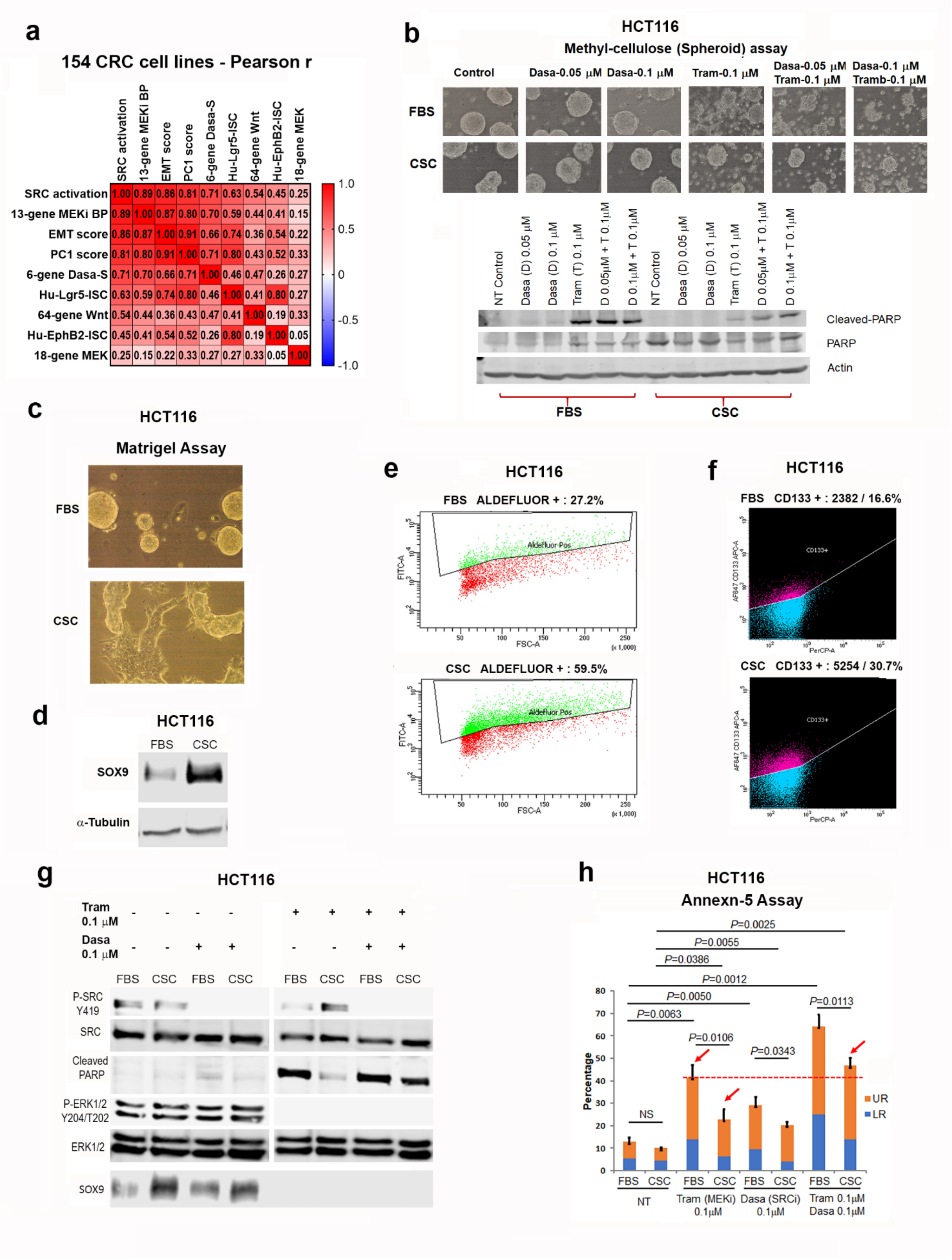

Fig S10. Correlation analysis of 154 CRC cell lines and in vitro drug treatment of HCT116 cells with MEKi + SRCi in CSC vs. non-CSC media. (**a**) Pearson correlation analysis of the 13-gene MEKi bypass

(BP) resistance, SRC activation, 5-gene dasatinib sensitivity (Dasa-S), EMT, PC1, Hu-Lgr5-ISC, Hu-EphB2-ISC, and 18-gene MEK pathway activation signature scores in Medico 154 CRC cell lines. (**b**) The methyl-cellulose (spheroid-enhancing) assay in HCT116 CRC cells. Cell cultures were grown in CSC medium (versus the non-CSC, regular control medium (FBS)) and were then treated with trametinib (MEKi) -/+ dasatinib (SRCi) for 72 hr. The top panel shows morphological changes, whereas the bottom panel shows Western blot analysis of cleaved PARP. The Matrigel assay. HCT116 cells that were grown in Matrigel with the CSC (vs non-CSC (FBS) media) display more mesenchymal-like morphology (**c**) and express higher SOX9, a CSC marker in CRC(9) as shown by Western analysis (**d**). Flow cytometry analysis shows that HCT116 cells grown in Matrigel with the CSC vs non-CSC (FBS) media significantly increased percentages of cells gated by ALDH1 expression (27.2% to 59.5%) (**e**) and by CD133 expression (16.6% to 30.7%) (**f**), two common CSC-surface markers in CRC(10). Next, HCT116 cells grown in Matrigel with the CSC vs non-CSC (FBS) media were treated by trametinib (MEKi) -/+ dasatinib (SRCi) for 48 hr followed by Western blot analysis (**g**) and Annexin-5 apoptosis assay (**h**). LR (blue) − apoptotic cells; UR (orange) − post-apoptotic dead cells. Bars represent standard deviation (n=3). *P* values are for two-tailed Welch's t test. The *in vitro* cell line analysis shows that MEKi-mediated apoptosis was markedly decreased for CRC cells grown in the CSC *vs.* non-CSC media (FBS). Drug resistance was accompanied by increased SRC activation (*P*-SRC Y419), supporting the notion that MEKi may induce *enhanced* AR in CSC *mediated by SRC*. Notably, inhibition of SRC by dasatinib in combination with the MEKi in the CSC medium was shown to increase apoptosis levels close to those induced by MEKi alone in the non-CSC (FBS) medium, as indicated by cleaved PARP and the Annexin-5 assay (**g,h**). Of note, Spheroid cultures or Matrigel cultures were grown in CSC vs non-CSC media for ~7 days followed by various drug treatment combinations for 2 or 3 days. In order to compare induction of apoptosis (by cleaved PARP/total PARP) and changes in cell signaling (by P-SRC/total SRC and P-ERK/total ERK) between CSC vs non-CSC media under the same experimental setting, we cut some of the blots according to anticipated protein sizes (kDa) prior to hybridization with different antibodies for Western blot analysis (see Additional File 8 for source data of blots).

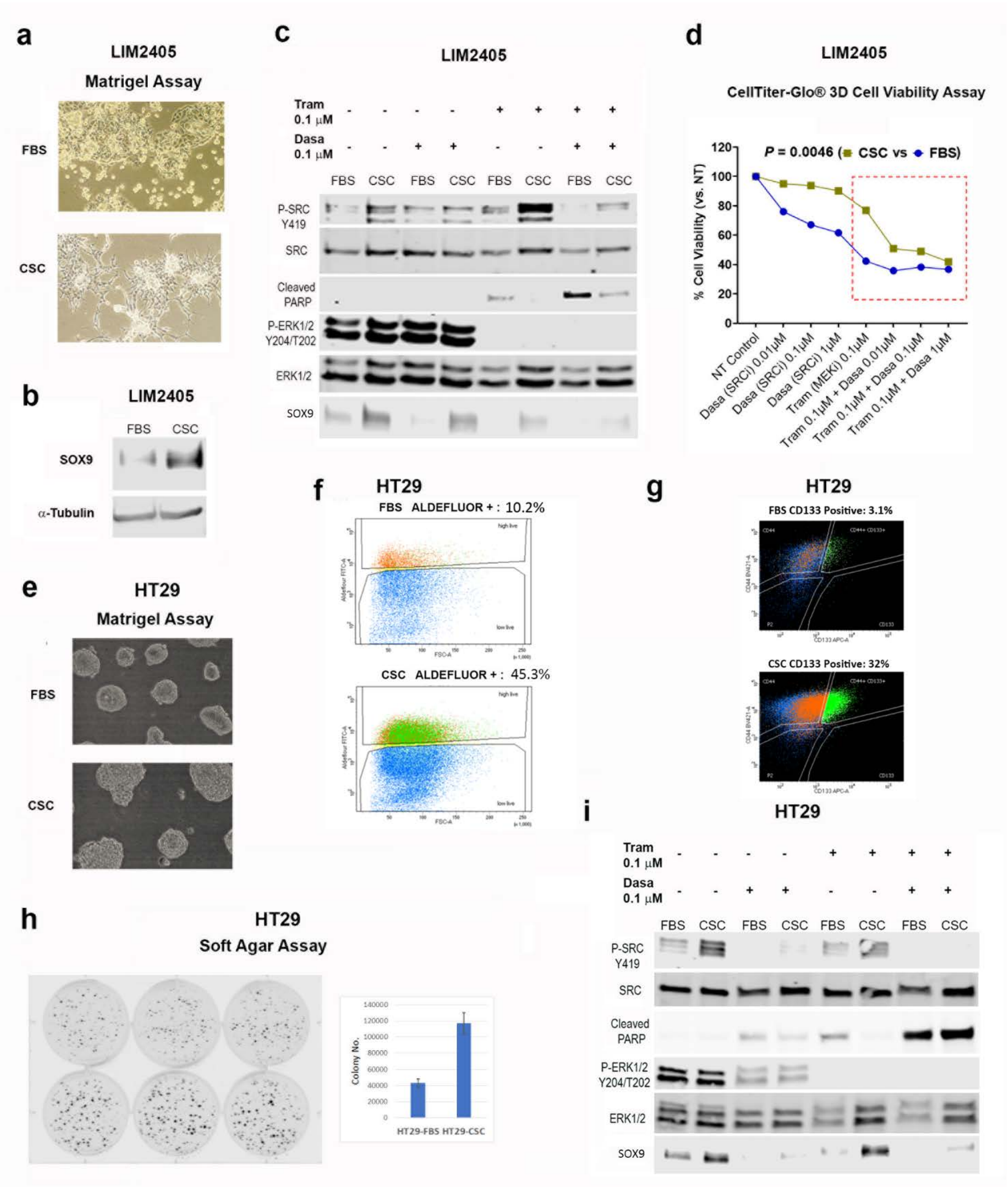

Fig S11. In vitro drug treatment of LIM2405 and HT29 cells with MEKi + SRCi in CSC vs. non-CSC media. The Matrigel assay. LIM2405 cells that were grown in Matrigel with the CSC (vs non-CSC (FBS)

media) display more mesenchymal-like morphology (**a**) and express higher SOX9, a CSC marker in CRC(9) as shown by Western analysis (**b**). LIM2405 cells grown in Matrigel with the CSC vs non-CSC (FBS) media were treated by trametinib (MEKi) -/+ dasatinib (SRCi) for 48 hr followed by Western blot analysis (**c**) and by CellTiter-Glo® 3D Cell Viability Assay (**d**). *P* value is for two-tailed Welch's t test when the cell viability was compared between CSC vs non-CSC (FBS) media. The *in vitro* cell line analysis shows that MEKi-mediated inhibition of cell growth was markedly decreased for CRC cells grown in the CSC *vs.* non-CSC media (FBS). Drug resistance was accompanied by increased SRC activation (*P*-SRC Y419), supporting the notion that MEKi may induce *enhanced* AR in CSC *mediated by SRC*. Notably, inhibition of SRC by dasatinib in combination with the MEKi in the CSC medium was shown to increase growth inhibition close to those induced by MEKi alone in the non-CSC (FBS) medium, as indicated by cleaved PARP, cell viability assay (**c,d**). HT29 cells that were grown in Matrigel with the CSC (vs non-CSC (FBS) media) display significantly larger colonies (**e**). Flow cytometry analysis shows that HT29 cells grown in Matrigel with the CSC vs non-CSC (FBS) media significantly increased percentages of cells gated by ALDH1 expression (10.2% to 45.3%) (**f**) and by CD133 expression (3.1% to 32%) (**g**), two common CSC-surface markers in CRC(10). (**h**) Soft agar assay shows that HT29 cells grown in Matrigel with the CSC vs non-CSC (FBS) media significantly increase the size and number of colonies in anchorage independent growth. Stained colonies were machine-counted by the Odyssey® DLx Imaging System (Li-COR). Two-tailed, paired t test was performed for comparison between CSC vs. non-CSC media. (**i**) HT29 cells grown in Matrigel with the CSC vs non-CSC (FBS) media were treated by trametinib (MEKi) -/+ dasatinib (SRCi) for 48 hr followed by Western blot analysis showing that MEKi + SRCi appeared to induce much enhanced apoptosis than MEKi only or SRCi only in either CSC or non-CSC (FBS) media. Of note, Matrigel cultures were grown in CSC vs non-CSC media for ~7 days followed by various drug treatment combinations for 2 days. In order to compare induction of apoptosis (by cleaved PARP/total PARP) and changes in cell signaling (by P-SRC/total SRC and P-ERK/total ERK) between CSC vs non-CSC media under the same experimental setting, we cut some of the blots according to anticipated

protein sizes (kDa) prior to hybridization with different antibodies for Western blot analysis (see Additional File 8 for source data of blots).

1. Huang F, Reeves K, Han X, Fairchild C, Platero S, Wong TW, et al. Identification of candidate molecular markers predicting sensitivity in solid tumors to dasatinib: rationale for patient selection. Cancer Res. 2007;67(5):2226-38.
2. Scott AJ, Song EK, Bagby S, Purkey A, McCarter M, Gajdos C, et al. Evaluation of the efficacy of dasatinib, a Src/Abl inhibitor, in colorectal cancer cell lines and explant mouse model. PLoS One. 2017;12(11):e0187173.
3. Medico E, Russo M, Picco G, Cancelliere C, Valtorta E, Corti G, et al. The molecular landscape of colorectal cancer cell lines unveils clinically actionable kinase targets. Nat Commun. 2015;6:7002.
4. Chaffer CL, Weinberg RA. A perspective on cancer cell metastasis. Science. 2011;331(6024):1559-64.
5. Vanharanta S, Massague J. Origins of metastatic traits. Cancer Cell. 2013;24(4):410-21.
6. Nieto MA, Huang RY, Jackson RA, Thiery JP. Emt: 2016. Cell. 2016;166(1):21-45.
7. Thiery JP, Lim CT. Tumor dissemination: an EMT affair. Cancer Cell. 2013;23(3):272-3.
8. Shibue T, Weinberg RA. EMT, CSCs, and drug resistance: the mechanistic link and clinical implications. Nat Rev Clin Oncol. 2017;14(10):611-29.
9. Aguilar-Medina M, Avendano-Felix M, Lizarraga-Verdugo E, Bermudez M, Romero-Quintana JG, Ramos-Payan R, et al. SOX9 Stem-Cell Factor: Clinical and Functional Relevance in Cancer. J Oncol. 2019;2019:6754040.
10. Dalerba P, Dylla SJ, Park IK, Liu R, Wang X, Cho RW, et al. Phenotypic characterization of human colorectal cancer stem cells. Proc Natl Acad Sci U S A. 2007;104(24):10158-63.
