# Supplementary material for: An integrative gene expression signature analysis identifies CMS4 KRAS-mutated colorectal cancers sensitive to combined MEK and SRC targeted therapy
Source: BMC Cancer. 2022 Mar 10;22:256. doi: 10.1186/s12885-022-09344-3 (PMC8908604; doi:10.1186/s12885-022-09344-3)
Supplement: Supplementary file 7 — Additional file7. 154 CRC cell lines_CMS_signaturescores.xls. [file 12885_2022_9344_MOESM7_ESM.docx]

**Original immunoblots for Fig S10 and S11**

**1. Original blots for Fig S10b**

**CSC**

**FBS**

NT control

Dasa (D) 0.05 μM

Dasa (D) 0.1 μM

Tram (D) 0.1 μM

D 0.05 μM + T 0.1 μM

D 0.1 μM + T 0.1 μM

NT control

Dasa (D) 0.05 μM

Dasa (D) 0.1 μM

Tram (D) 0.1 μM

D 0.05 μM + T 0.1 μM

D 0.1 μM + T 0.1 μM

**
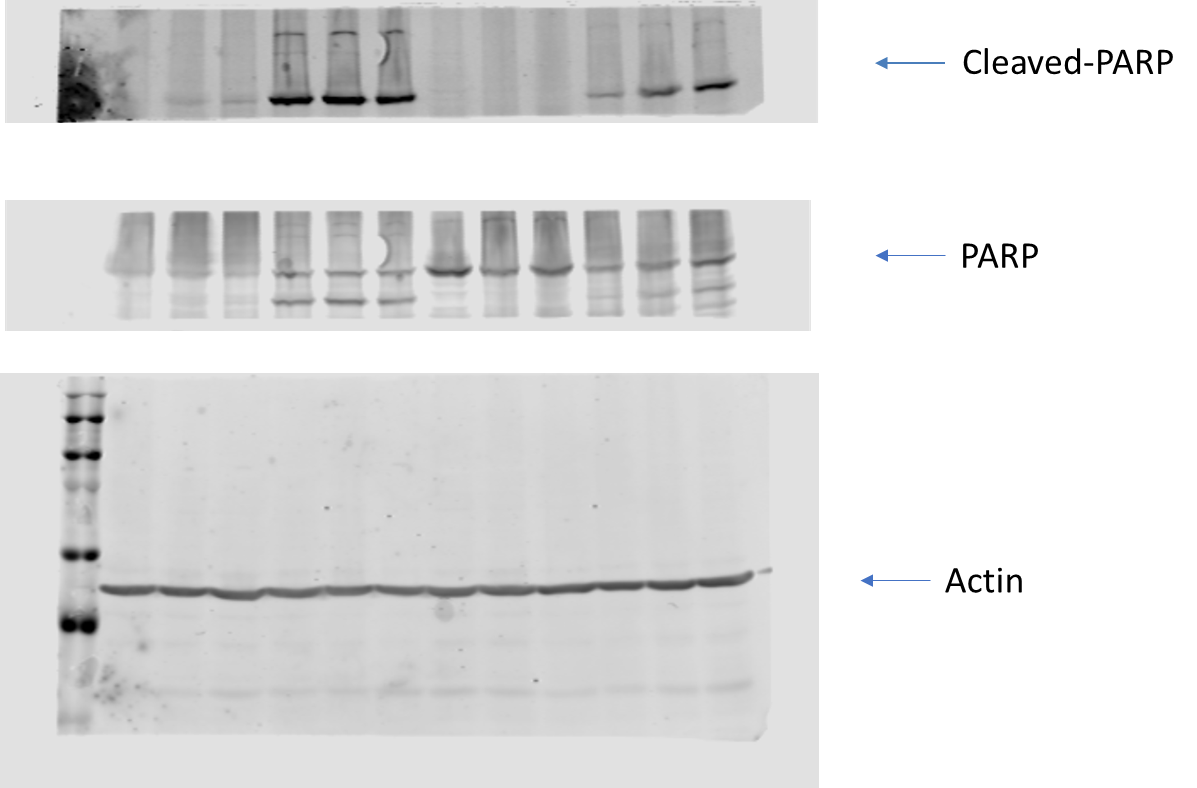
**

**2. Original blots for Fig S10d**


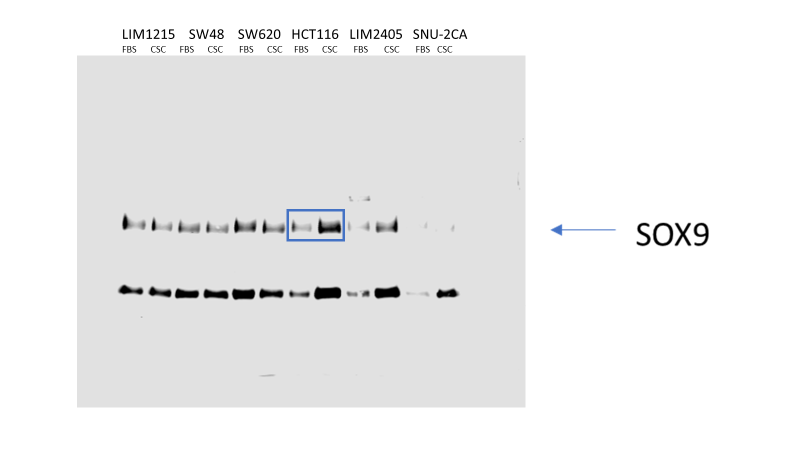


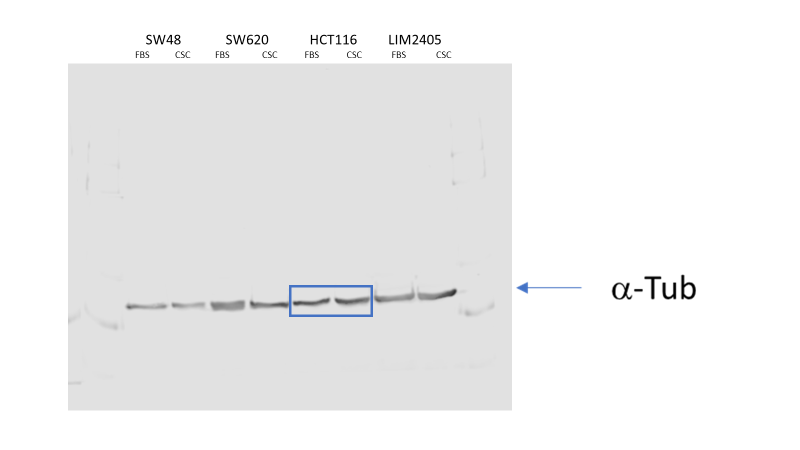


Note that only lanes 7 and 8 (top panel) and lanes 5 and 6 (bottom panel) on the respective blots were used. Both Gels were loaded with the same amount of proteins.

**3. Original blots for Fig S10g**

**
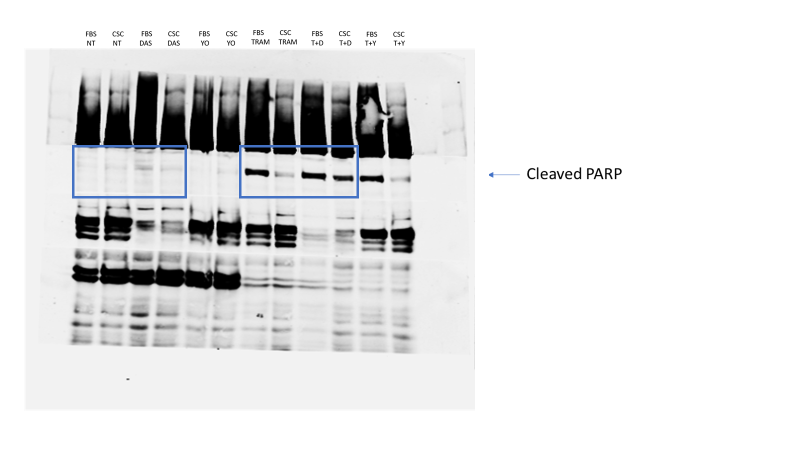
**

**­** **
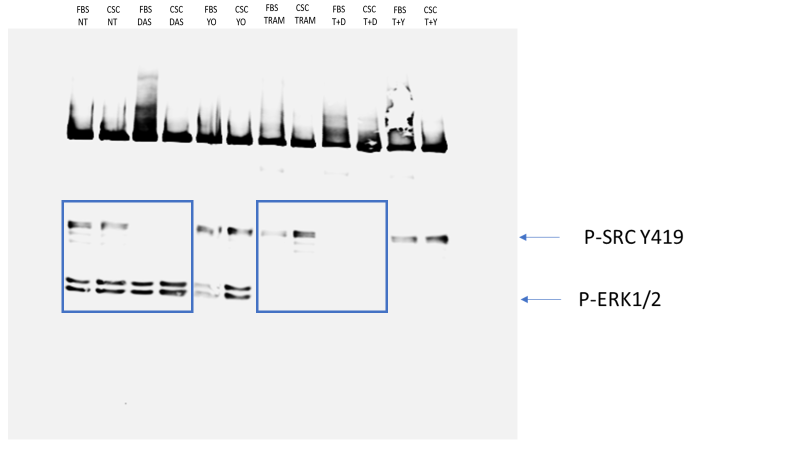
**


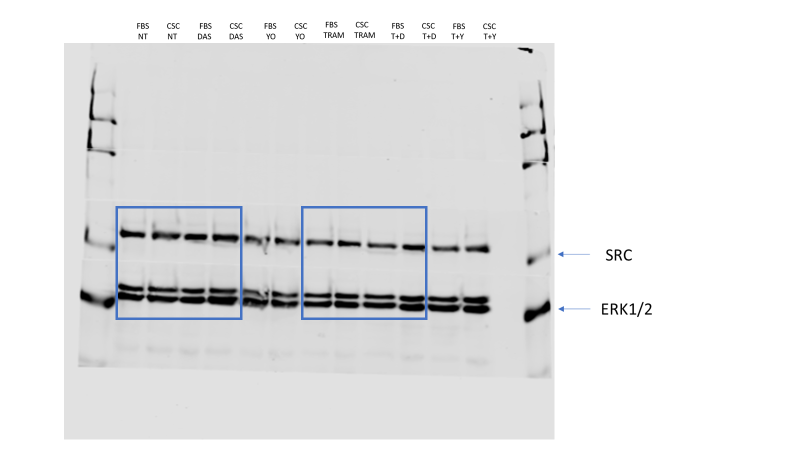


Note that for Figure S10g only lanes 1-4 and 7-10 were used. Lanes 5, 6, 11, and 12 were samples treated with a NOTCH inhibitor and trametinib combination. This data was irrelevant to this paper and thus omitted.


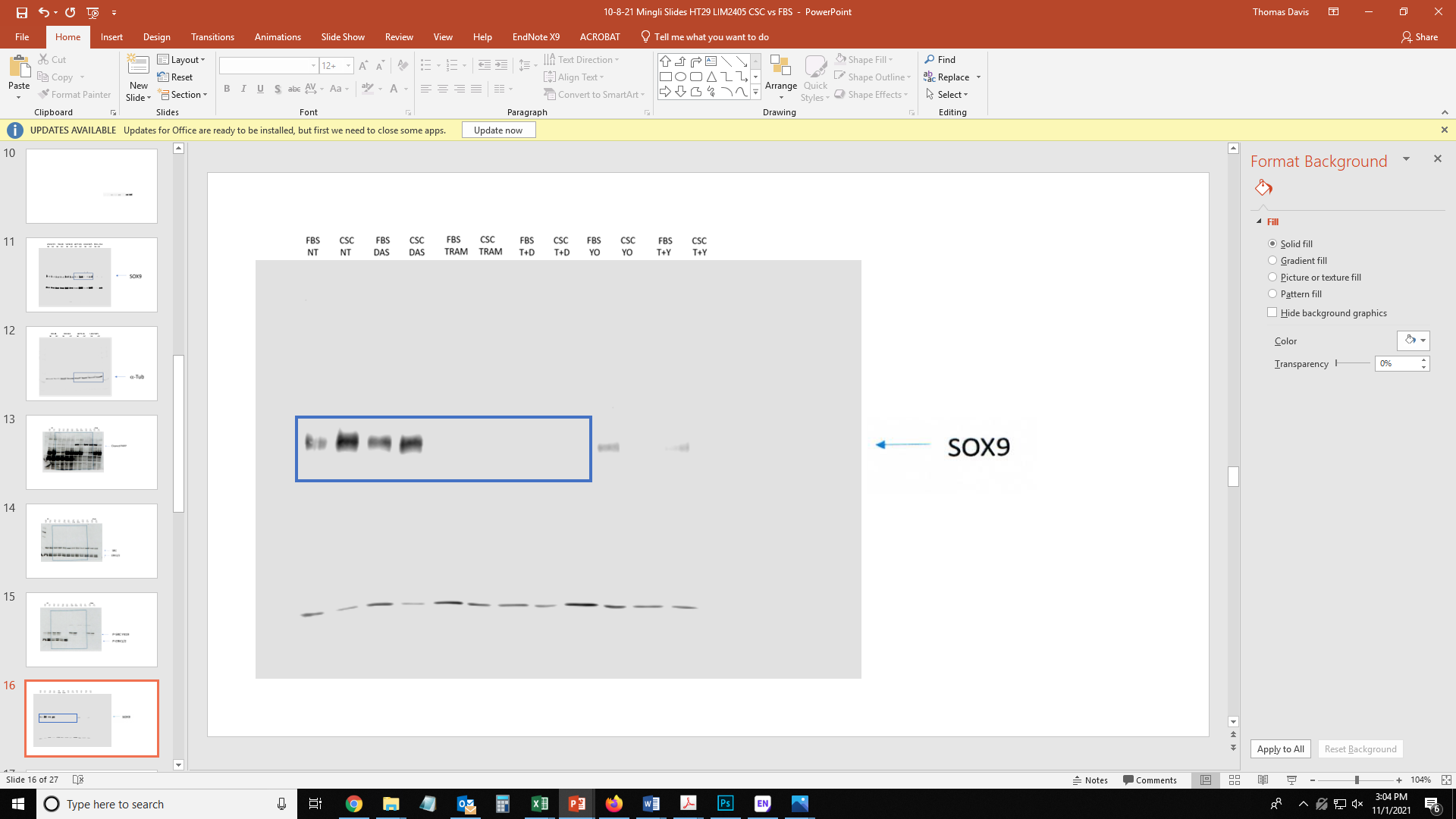


Note that for Figure S10g only lanes 1-8 were used from this blot. Lanes 9-12 were samples treated with a NOTCH inhibitor and trametinib combination. This data was irrelevant to this paper and thus omitted.

**4. Original blots for Fig S11b**


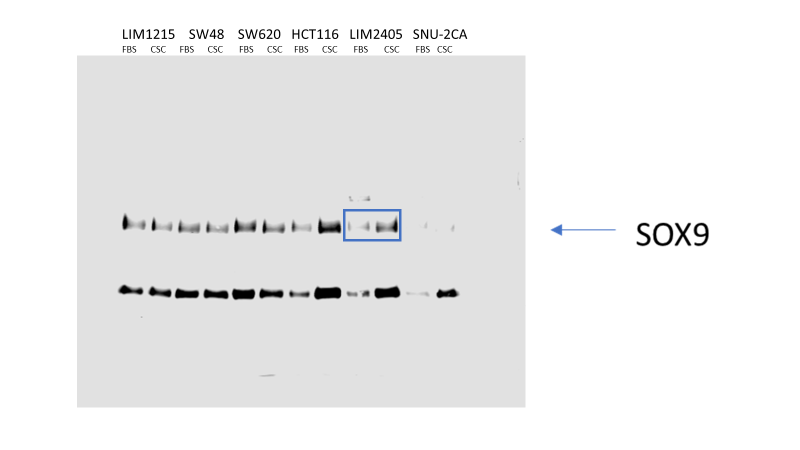


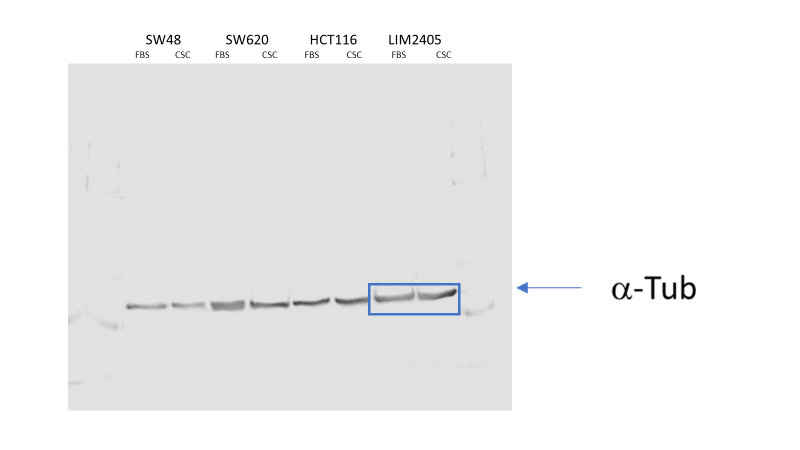


Note that only lanes 9 and 10 (top panel) and lanes 7 and 8 (bottom panel) on the respective blots were used. Both Gels were loaded with the same amount of proteins.

**5. Original blots for Fig S11c**


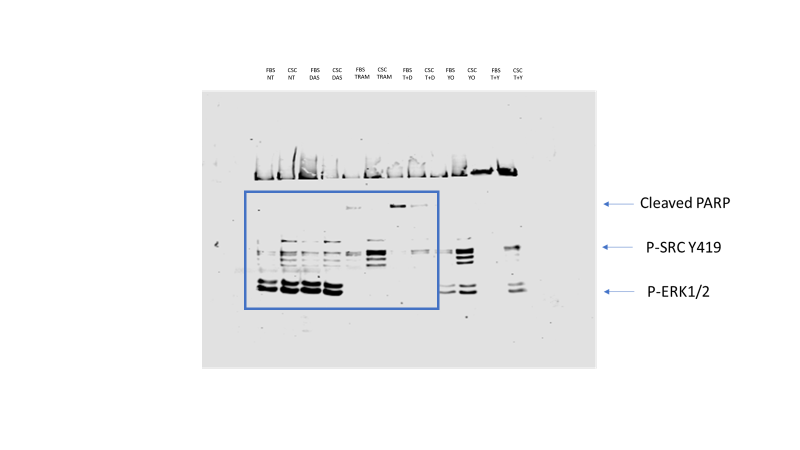


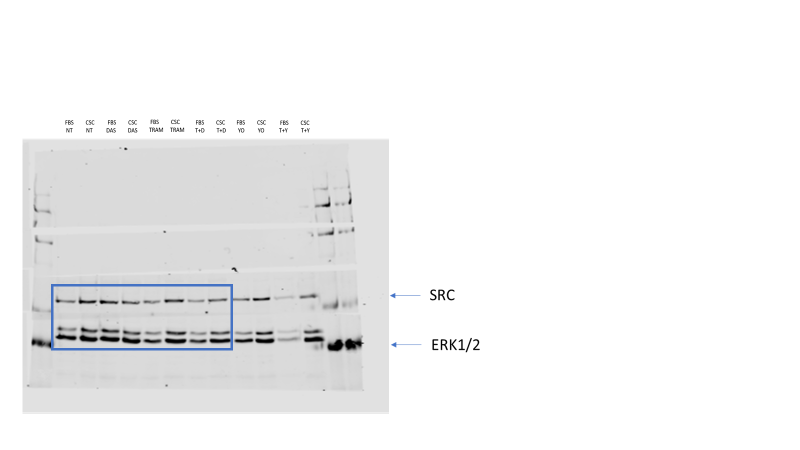


Note that for Figure S11c only lanes 1-8 were used from these blots for drug treatment with Trametinib and/or Dasatinib. Lanes 9-12 data were not used here because they were for other drug treatments.


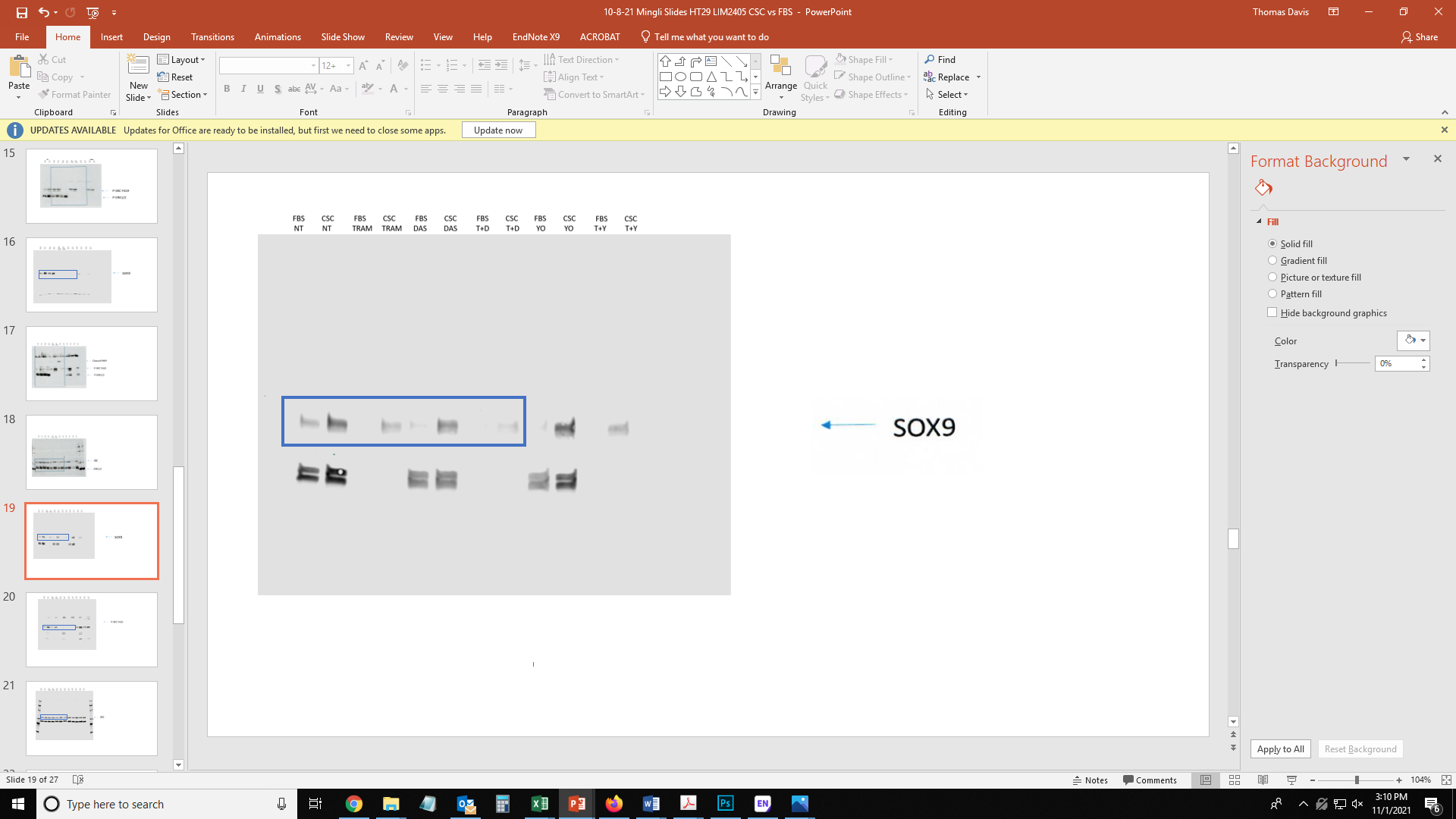


Only the first 8 lanes were used but had to be re-arranged. Lanes 5 and 6 were switched with lanes 3 and 4 to conform to our previous formatting. Lanes 9-12 data were not used here because they were for other drug treatments.

**6. Original blots for Fig S11i**


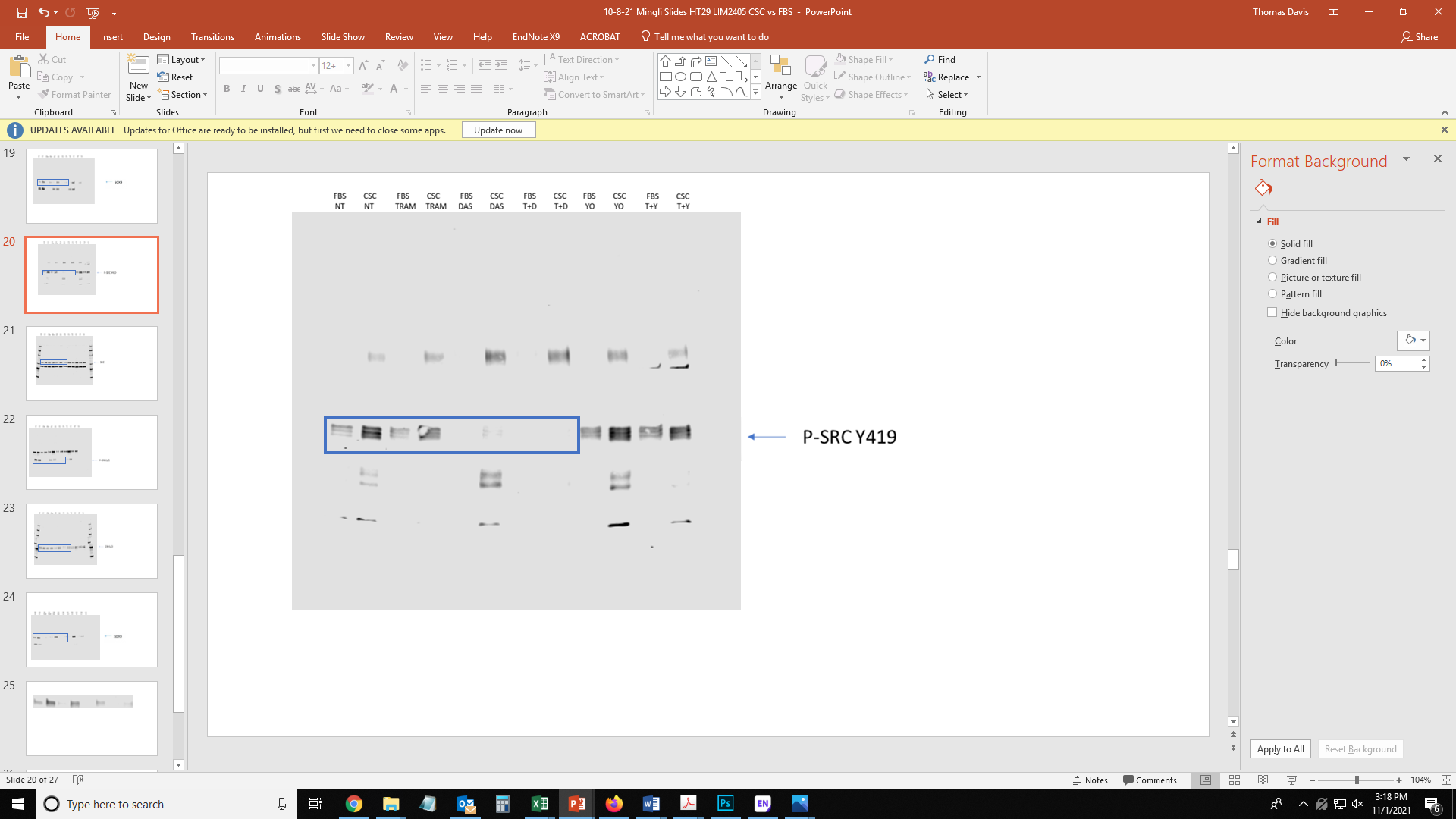


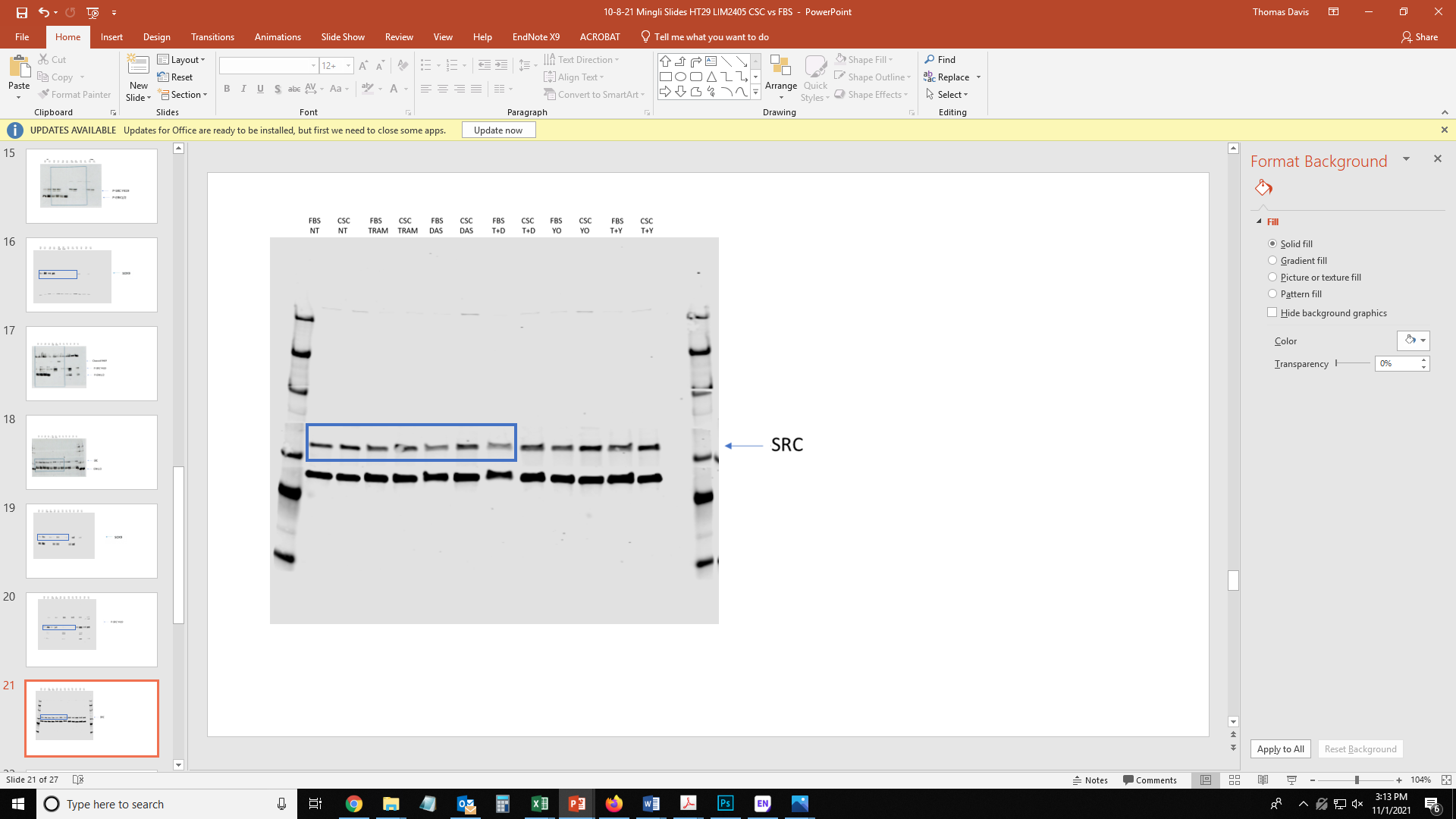


Only the first 8 lanes were used but had to be re-arranged. Lanes 5 and 6 were switched with lanes 3 and 4 to conform to our previous formatting. Lanes 9-12 data are not used here because they were for other drug treatments.


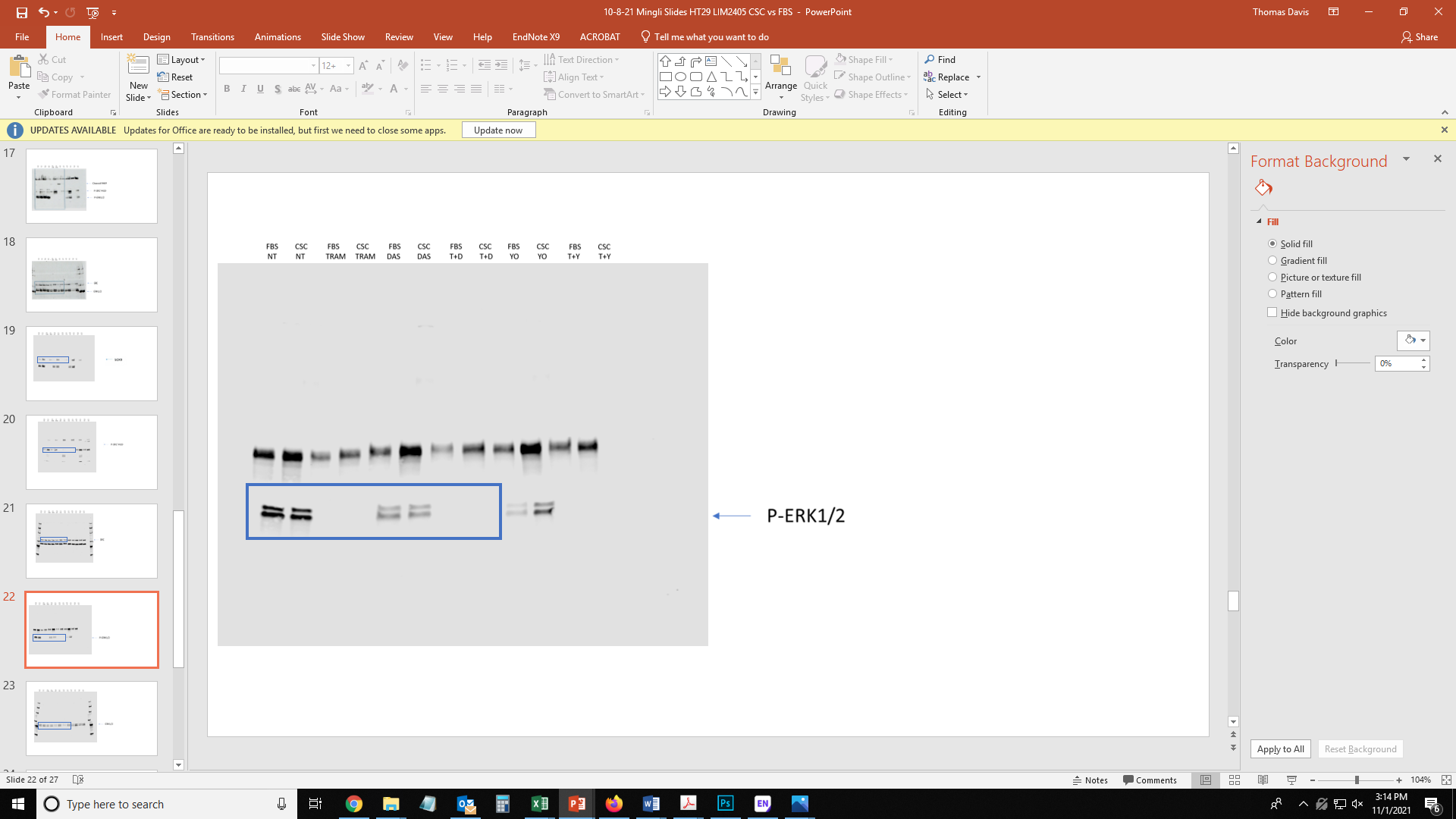


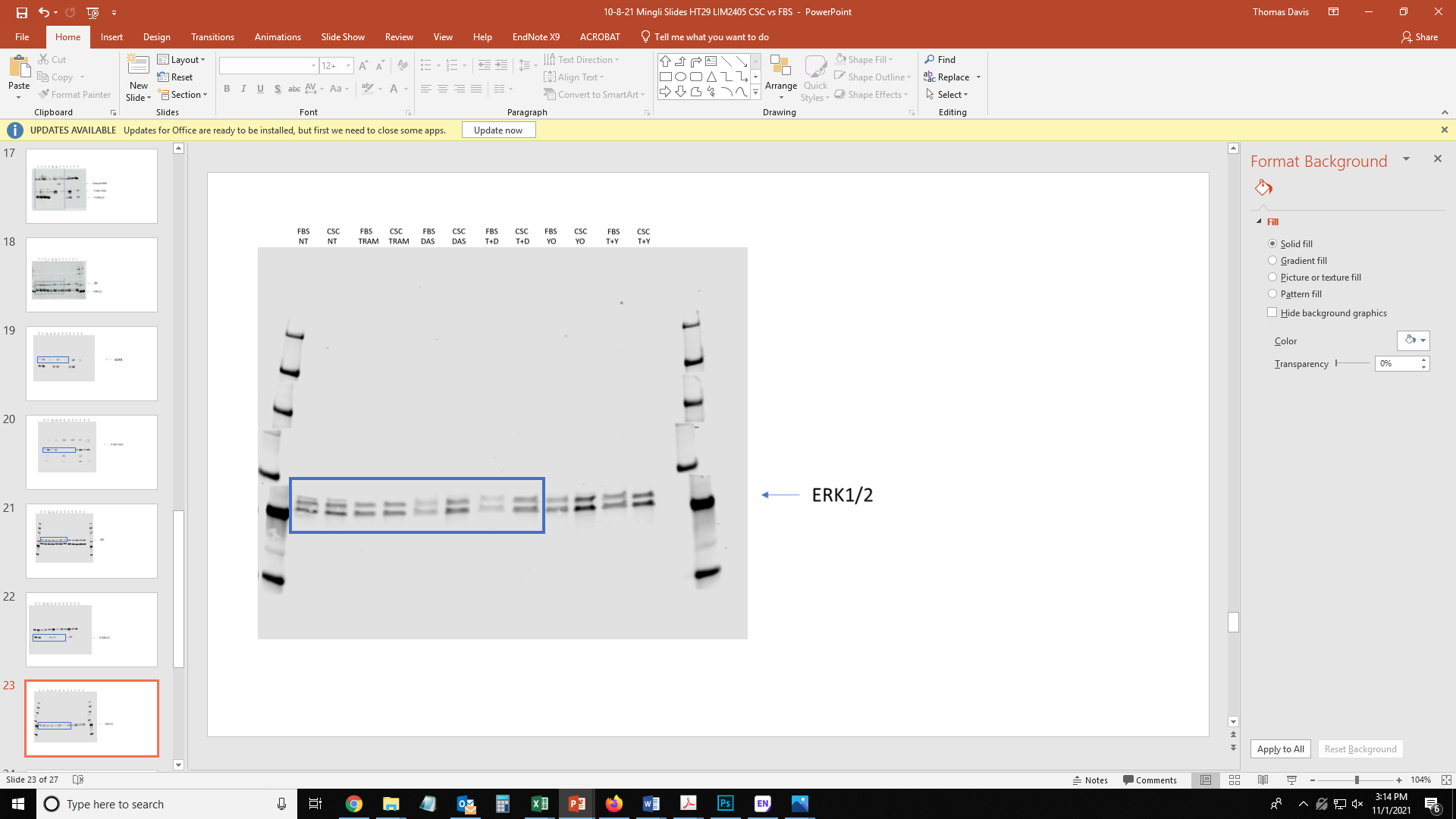


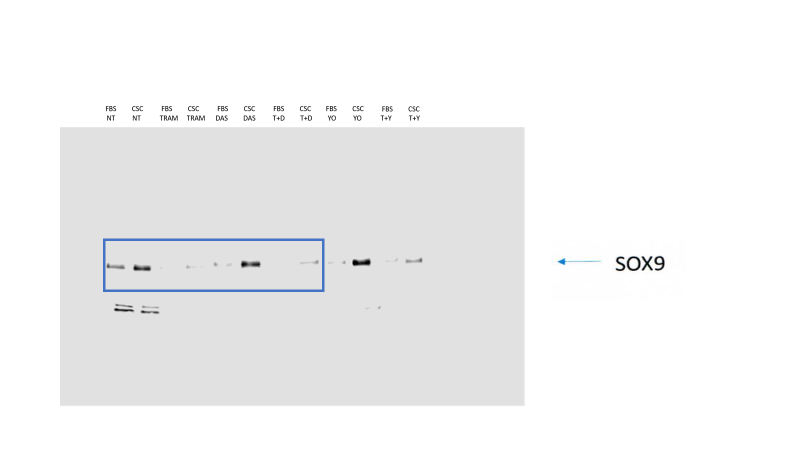


Only the first 8 lanes were used but had to be re-arranged. Lanes 5 and 6 were switched with lanes 3 and 4 to conform to our previous formatting. Lanes 9-12 data are not used here because they were for other drug treatments.


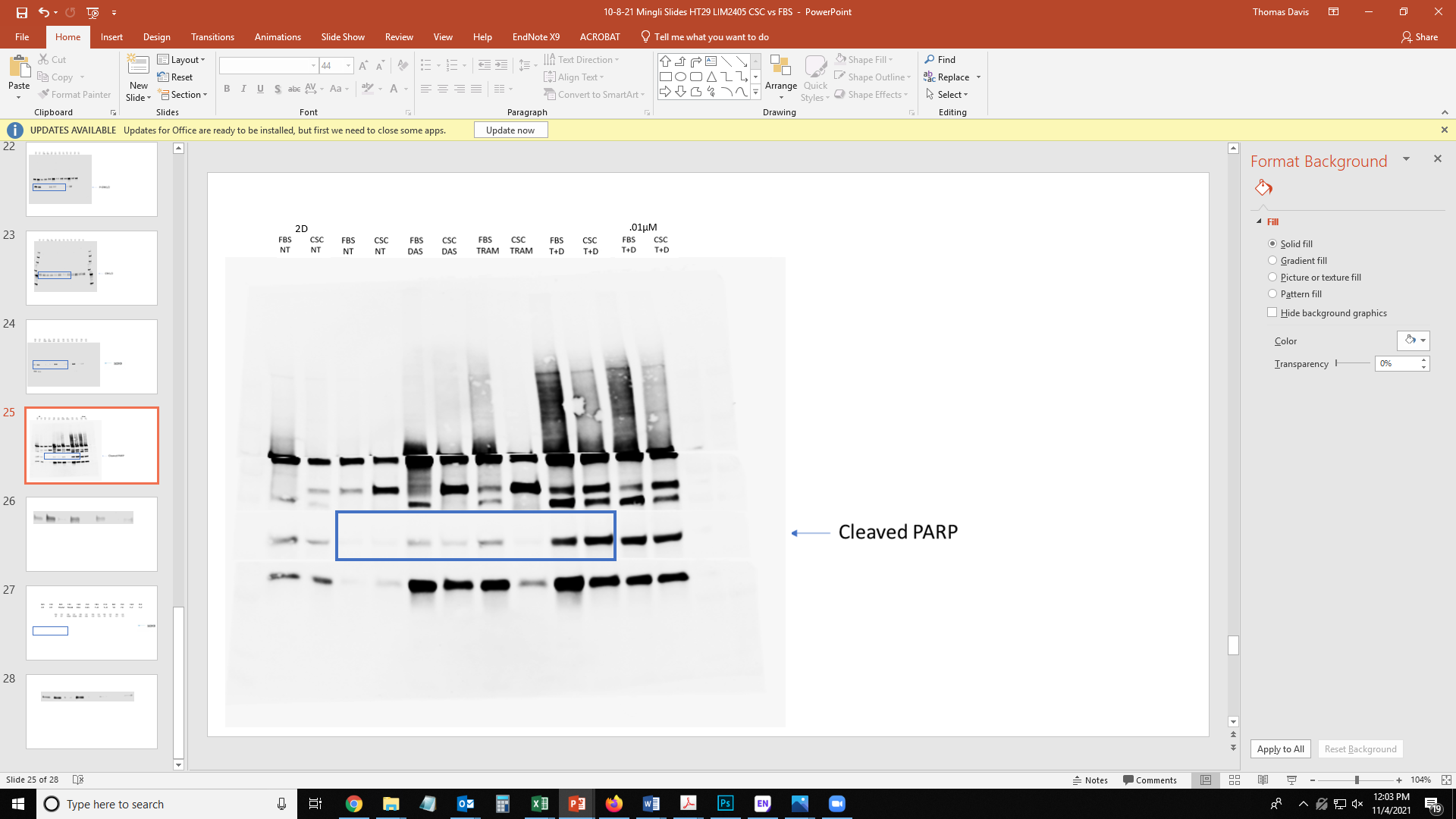


Lanes 3-10 were used. Lane 1 and 2 were from 2D grown cells and lanes 11 and 12 were done at a lower concentration, neither of which were relevant.
